# Device for measuring part adhesion in FFF process

**DOI:** 10.1016/j.ohx.2022.e00258

**Published:** 2022-01-07

**Authors:** Daniel Laumann, Dieter Spiehl, Edgar Dörsam

**Affiliations:** Technical University of Darmstadt, Department of Mechanical Engineering, Institute for Printing Science Technology, Magdalenenstraße 2, 64289 Darmstadt, Germany

**Keywords:** 3D-Printing, Fused filament fabrication, Adhesion

## Abstract

The adhesion of parts to the build surface plays a central role in the Fused Filament Fabrication (FFF) process. Without sufficient adhesion, the part will deform (so called warping) due to thermal shrinkage, so that no defined geometries can be created. Nevertheless, there is no established method to measure the adhesion of printed parts and therefore it is not possible to targeted improve it. This article presents a measurement method based on the DIN EN 28510-1 standard and a corresponding test device which makes it possible to identify the optimum build surface for a filament and also to improve the process parameters in a targeted manner. The test device combines a FFF printer with a measuring unit so that all common filaments can be tested close to the process up to a processing temperature of 400 °C in the nozzle and around 150 °C on the build platform. The test device uses only open-source parts and software and costs about 1700€.

## Graphical abstract

**Figure:**
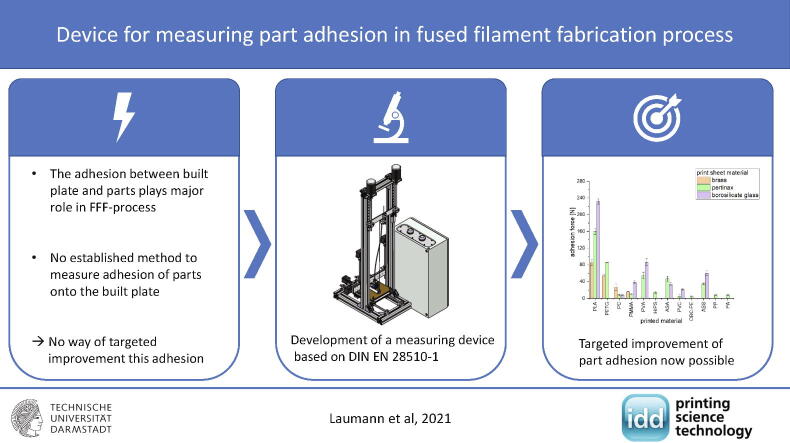

## Hardware in context

Due to the great variety of processable materials [1] and the low costs of the machines [2] Fused Filament Fabrication (FFF), also called material extrusion (MEX), is one of the most used additive manufacturing processes [3]. Not only private customers use this technique but also many industries like aerospace, automotive [4] or medicine. Printing with high performance materials allows the manufacturing of implants [5] or heat-sterilizable COVID-19 facemasks [6]. Other materials like special Polylactides can be used for the curing of bone diseases [7]. A common issue of MEX is the too low adhesion of the part to the build surface during fabrication [8]. Deformations of the fabricated parts caused by the too low adhesion are called warping [9]. They could be strong enough to detach the entire part from the build platform. In this case the manufacturing process fails due to the undefined position of the already fabricated part and the resulting undefined adding of material [10]. Only a sufficient adhesion between part and build surface can prevent warping during the printing process. Warping can also occur after removing the print from the build surface due to thermal stresses caused by a too fast cooling down. This type of warping is not addressed by the here described measurement device.

Despite the widespread usage of material extrusion there is no standard to determine which process parameters deliver the best adhesion nor to check if the printed material is compatible with the chosen build surface. Very few articles describe first researches in this area. M. Kujawa [11] developed a measurement method based on tensile tests to determine the adhesion forces of a printed part to the build surface. M. Spoerk [10], [12] used a testing device which shears printed strands off. This preliminary work was taken up and a testing device based on the standard DIN EN 28510–1 was developed. This norm is a well-known standard in the adhesives industry and defines a uniform procedure and geometry of specimens for the measurement of adhesion forces. It is also a uniform reference to judge the usability of measurement results. Additionally, the 90° -peeling test described in DIN EN 28510–1 simulates an adhesion failure similar to the failure which occurs under real conditions in MEX. In both cases the part begins to peel off at one point and the loss of contact between part and build surface is progressing from this point.

## Hardware description

**Fig. 1:**
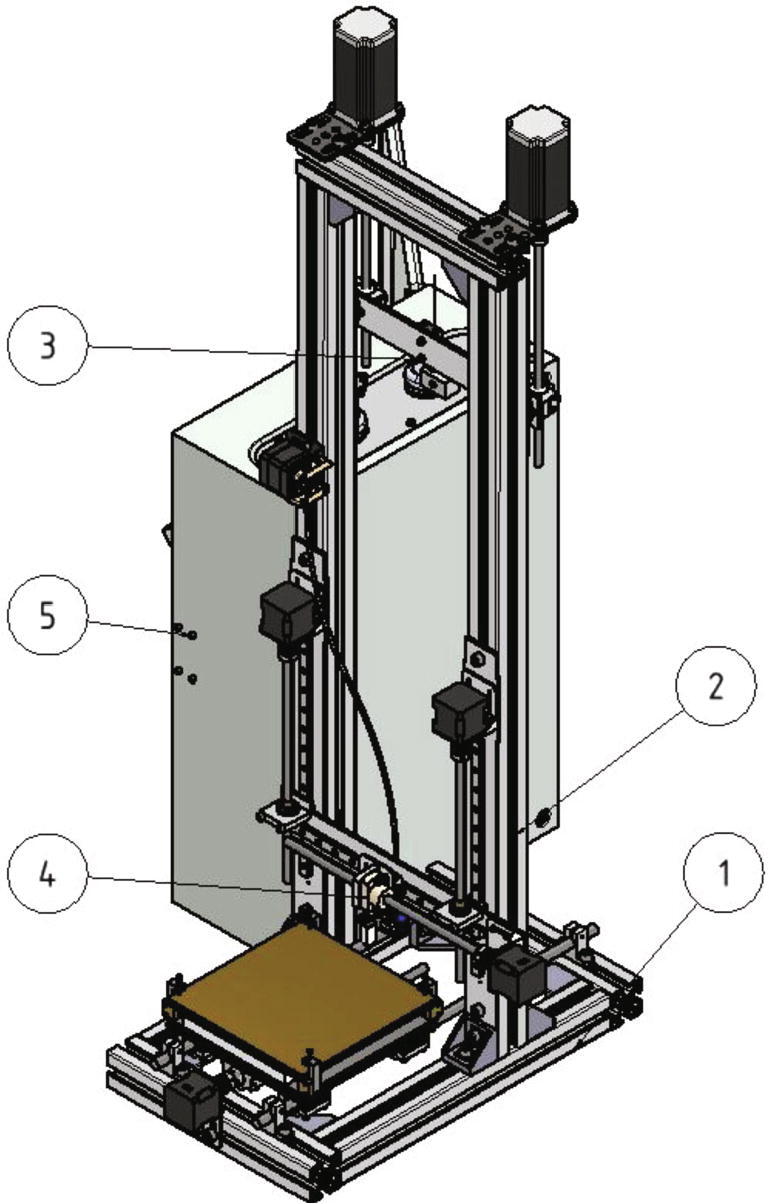
Overview of the measurement device: bottom frame (no. 1), top frame (no. 2), measurement unit (no. 3), MEX-system (no. 4), control cabinet with electronics (no. 5).

The measurement device described here is a MEX-system combined with a classic tensile testing machine. Due to the integration of the tensile testing machine into the 3D-printer, adhesion forces can be measured under nearly real process conditions. It is possible to measure the adhesion forces between the printed part and the print directly after finishing the print. Also, the process temperatures can be kept during the measurement cycle. Fig. 1 shows an overview of the measurement device and the position of its five main assemblies can be seen. In the following the surface which will be printed on is called build surface. It is mounted to a assembly called build platform (see Fig. 49). The MEX-system has a build volume of 150 mm × 200 mm × 200 mm and the whole device measures 700 mm × 610 mm × 1200 mm. The measurement device described here was designed to provide a customizable platform to test the adhesion of parts under real process conditions.

Special attention was paid to make the exchange of hardware parts as easy as possible. Extrusion head, build surface and load cell can easily be replaced: The extrusion head is clamped to the positioning system via a groove mount which is a common standard [13]. Therefore, a great variety of different extrusion heads can be attached to the positioning system without any modification of the mount necessary. The suggested E3Dv6 extrusion head is capable of printing all materials up to a nozzle temperature of 280 °C in standard configuration. It can be changed to a high temperature extrusion head to print materials with necessary nozzle temperatures up to 400 °C.

The build surface is clamped with four screws to the y-axis carriage and can therefore easily be replaced. To ensure a stable and repeatable z-axis calibration with all possible surfaces, a BL-Touch [14] was used as z-probe. The used heating element is capable to heat the build surface up to 150 °C which allows the printing of high temperature polymers like polyetheretherketone [5]. As a controller board a Duet 3 is used to control the MEX-system and the motors of the measurement unit. This board allows an easy adaption of the firmware to new hardware. Instead making changes in the source code of the firmware only a configuration file must be adapted. It is also possible to generate for each used hardware setup a specific configuration file. Due to the used CAN bus system it is also easy (in comparison to other available controller boards) to expand the system to further tasks or to automate the measurement process.

The measurement unit consists of a linear axis with a load cell like standard tensile testing machines. Connected to the load cell is a wire with a hook which allows to apply peeling forces to a printed specimen when the linear axis moves upwards. The values of the load cell are read by an Arduino Uno with an amplifier board. A self-written Python-script converts the values sent by the Arduino Uno into forces and saves them into a csv-file. The measuring range of the peeling forces can easily be adapted by replacing the load cell to another and adapt the conversion values in the Python-script.

The combination of a peeling test unit with a highly adaptable fused filament fabrication printer allows the device

• … to measure the adhesion of printed parts under real process conditions with a method similar to the observed failure mechanism
• … to test a wide variety of filaments and build surfaces
• … to be easily upgraded or reconfigured due to the fully open-source software, firmware and hardware
• … to suit other use cases like the measurement of interlayer adhesion or adhesion between different plastics in multi material printing

## Design files

The design file summary is shown in Table 1. All parts except the insulation and the measuring unit mechanical endstop mount are made out of aluminum. The insulation is made from calcium silicate and the measuring unit mechanical endstop mount is 3d-printed out of Polylactide.

**Table 1:** Overview of the design files with the file type, the license and the repository location.

**Part number**	**Design filename**	**Picture**	**File type**	**Open source license**	**Location of the file**
1	adapter_crossbar_ to_load_cell		STEP-File (.stp)	CC BY 4.0	Reserved https://doi.org/10.17632/c77trxsr58.1
2	adapter_load_cell		STEP-File (.stp)	CC BY 4.0	Reserved https://doi.org/10.17632/c77trxsr58.1
3	base_plate		STEP-File (.stp)	CC BY 4.0	Reserved https://doi.org/10.17632/c77trxsr58.1
4	build_platform_bottom_plate		STEP-File (.stp)	CC BY 4.0	Reserved https://doi.org/10.17632/c77trxsr58.1
5	build_platform_top_plate		STEP-File (.stp)	CC BY 4.0	Reserved https://doi.org/10.17632/c77trxsr58.1
6	connection_x_axis_lead_screw		STEP-File (.stp)	CC BY 4.0	Reserved https://doi.org/10.17632/c77trxsr58.1
7	crossbar		STEP-File (.stp)	CC BY 4.0	Reserved https://doi.org/10.17632/c77trxsr58.1
8	two extrusion_head_connectors		STEP-File (.stp)	CC BY 4.0	Reserved https://doi.org/10.17632/c77trxsr58.1
9	insulation		STEP-File (.stp)	CC BY 4.0	Reserved https://doi.org/10.17632/c77trxsr58.1
10	measuring_unit_mechanical_endstop_mount		STEP-File (.stp)	CC BY 4.0	Reserved https://doi.org/10.17632/c77trxsr58.1
11	x_axis_carriage_angle		STEP-File (.stp)	CC BY 4.0	Reserved https://doi.org/10.17632/c77trxsr58.1
12	four spacers		STEP-File (.stp)	CC BY 4.0	Reserved https://doi.org/10.17632/c77trxsr58.1
13	x_axis_plate		STEP-File (.stp)	CC BY 4.0	Reserved https://doi.org/10.17632/c77trxsr58.1
14	two z_axis_plates		STEP-File (.stp)	CC BY 4.0	Reserved https://doi.org/10.17632/c77trxsr58.1
15	hook		STEP-File (.stp)	CC BY 4.0	Reserved https://doi.org/10.17632/c77trxsr58.1
16	z_probe_connector		STEP-File (.stp)	CC BY 4.0	Reserved https://doi.org/10.17632/c77trxsr58.1
17	four build_surface_clamps		STEP-File (.stp)	CC BY 4.0	Reserved https://doi.org/10.17632/c77trxsr58.1
18	two connection_plates_z_axis		STEP-File (.stp)	CC BY 4.0	Reserved https://doi.org/10.17632/c77trxsr58.1
	Measuring-software ”adhesion measurement.py”	---	Python-File (.py)	CC BY 4.0	Reserved https://doi.org/10.17632/c77trxsr58.1
	measuring-software configuration file ”config.json”	---	JSON-File (.json)	CC BY 4.0	Reserved https://doi.org/10.17632/c77trxsr58.1
	Arduino-software ”adhesion measurement arduino.ino”	---	Arduino- File (.ino)	CC BY 4.0	Reserved https://doi.org/10.17632/c77trxsr58.1
	Duet3-configuration ”config.g”	---	Config-File (.g)	CC BY 4.0	Reserved https://doi.org/10.17632/c77trxsr58.1
	Duet3 Makro ”measurement. g”	---	Makro-file (.g)	CC BY 4.0	Reserved https://doi.org/10.17632/c77trxsr58.1
	3D-model of the specimen	---	STL-file (.stl)	CC BY 4.0	Reserved https://doi.org/10.17632/c77trxsr58.1
	presliced model of the specimen	---	G-Code-file (.gcode)	CC BY 4.0	Reserved https://doi.org/10.17632/c77trxsr58.1

### Design file summary

**adapter_crossbar_to_load_cell** is the connecting piece between the crossbar of the measurement unit and the load cell **adapter_load_cell** is the connecting piece between the wire with the hook and the load cell **build_platform_bottom_plate** is the lower edging for the insulation **build_platform_top_plate** is the upper edging of the insulation **connection_x_axis_lead_screw** is the mount of the high helix thread lead screw for the x-axis **crossbar** is the base plate for the measuring unit **extrusion_head_connector** is the connection between the extrusion head and the x-axis carriage **insulation** insulates the heated build platform downwards. Calcium silicate is dusting therefore the insulation is edged between two metal sheets **measuring_unit_mechanical_endstop_mount** is a MEX printed part to attach the mechanical endstop of the measuring unit to the frame **x_axis_carriage_angle** is a base part which connects extrusion head, slide, z-probe and lead screw with each other. It can be easily made out of a CNC Motor angle for NEMA 17 as listed in the bill of materials. **spacer** is a part which defines the correct spacing and alignment of the build platform **x_axis_plate** is a base plate which connects the z-axis of the MEX-system with all parts of the x-axis assembly **z_axis_plate** is the base plate of the z-axis assembly and connects the linear guide and the stepper motor of the z-axis together **z_probe_connector** is a part to attach the z-probe to the x-axis carriage **build_surface_clamp** is a part to clamp the build surface to the build platform **measuring-software** is a Python-script to start a measurement cycle, read meanwhile the values of the load cell and writes them to a .csv-File **measuring-software configuration file** is a JSON-file which allows to configure the measurement-software as described in Table 4. **Arduino-software** is the code which must be uploaded to the Arduino Uno. It sends the values read from the load cell to the measuring software via a serial connection. **Duet3-configuration** is a configuration file which provides all information (like dimensions of the print area, allowed process temperatures, etc.) to the Duet firmware **Duet3 Makro ”measurement cycle”** is a macro which is launched by the measurement software via a http-request and contains movement commands for the measuring unit. **3D-model of the specimen** is a printable STL-file of an adhesion test specimen. It is based on the description of DIN EN 28510-1. **presliced model of the specimen** is a presliced model of the specimen. It can only be printed with the extrusion head and the extruder used in this manual. The printing parameters are following: nozzle diameter 1.0 mm, filament diameter 1.75 mm, layer height and first layer height 0.5 mm, printing speed 10 mm/s, nozzle temperature 230 °C, build platform temperature 70 °C (Table 2).

## Bill of materials summary

**Table 2:** Bill of materials with all parts needed to be purchased.

**Designator**	**Component**	**Number**	**Cost per unit [€]**	**Total cost [€]**	**Source of materials**
**Frame**					
profile 45 × 45L B-Type slot 10 300 mm	019,612	3	3.24	9.72	Motedis
profile 45 × 45L B-Type slot 10 400 mm	019,612	2	4.32	8.64	Motedis
profile 45 × 45L B-Type slot 10 985 mm	019,612	2	10.64	21.28	Motedis
Bracket 45B-Type slot 10	S10BBR45	1 Bag	10.02	10.02	Motedis
*T*-nut B-Type slot 10 [M8	S10BHASNM8	1 Bag	13.78	13.78	Motedis
**Screws**					
Screw cylinder head with hexagon socket M3 × 6		40	0.13	6.29	Motedis
Screw cylinder head with hexagon socket M3 × 8		17	0.13	2.21	Motedis
Screw cylinder head with hexagon socket M3 × 10		24	0.13	3.84	Motedis
Screw cylinder head with hexagon socket M3 × 12		12	0.13	2.07	Motedis
Screw cylinder head with hexagon socket M3 × 16		10	0.13	1.3	Motedis
Screw cylinder head with hexagon socket M3 × 20		4	0.13	0.91	Motedis
Screw cylinder head with hexagon socket M3 × 45		1 Bag	8.07	8.07	Schraubenhandel24
Screw cylinder head with hexagon socket M3 × 50		1 Bag	6.90	6.9	Schraubenhandel24
Screw cylinder head with hexagon socket M4 × 6		4	0.13	0.91	Motedis
Screw cylinder head with hexagon socket M4 × 8		10	0.13	1.78	Motedis
Screw cylinder head with hexagon socket M4 × 10		8	0.13	0.91	Motedis
Screw cylinder head with hexagon socket M4 × 20		2	0.13	0.61	Motedis
Screw cylinder head with hexagon socket M4 × 40	7984-2-4 × 40/100	1 Bag	11.94	11.94	Schraubenhandel24
Screw cylinder head with hexagon socket M5 × 16		10	0.13	1.82	Motedis
Screw cylinder head with hexagon socket M5 × 40	4,050,571,128,449	4	0.09	0.36	Landefeld
Screw cylinder head with hexagon socket M6 × 8		2	0.13	0.61	Motedis
Screw cylinder head with hexagon socket M8 × 16		42	0.13	6.59	Motedis
countersunk screw with hexagon socket M3 × 6		4	0.13	0.92	Motedis
countersunk screw with hexagon socket M4 × 35		18	0.13	3.02	Motedis
grub screw M3 × 4	2-0913-3 × 4	12	0.08	0.96	v2a-schrauben-billiger
grub screw M4 × 6		4	0.13	0.92	Motedis
**Nuts**					
Nut DIN 934 M3		4 Bags	0.03	1.13	Motedis
Nut DIN 934 M4		1 Bag	0.03	1.13	Motedis
Nut DIN 934 M5		1 Bag	0.03	0.38	Motedis
**Distance bolts**					
Distance bolt DIN N0373 M3 × 10 SW5,5		1 Bag	1.50	1.50	Motedis
bolt DIN N0373 M4X15 SW7		1 Bag	2.00	2.00	Motedis
**Washer**					
Washer DIN 125 - for M3		1 Bag	0.75	0.75	Motedis
**Linear guides**					
Linear guide rail Miniature MR12M-N, L = 300 mm		5	24.39	145.11	Motedis
Guide Carriage Miniature MR12MN-SS-V0-N		3	19.59	58.76	Motedis
Guide Carriage Miniature MR12ML-SS-V0-N		2	22.02	44.03	Motedis
Shaft supports SH12/SK12 SH12/SK12	SH12/SK12	4	1.77	6.13	Motedis
Precision shaft 12 mm h6 - steel - hardened and ground 500 mm		2	3.15	6.30	Motedis
Linear bearing 12 mm SCJ12UU adjustable clearance	SC-AJ12	4	4.34	20.96	Motedis
**High helix threaded spindles**					
drylin® lead screw, dryspin® high helix thread, right-hand thread, 1.4301 (304) stainless steel l = 300 mm	DST-LS-10X25-R-ES	2	34.36	68.72	Igus
dryspin® lead screw nut, high helix thread, JSRM	DST-JSRM-2220DS10X25	1	15.45	15.45	Igus
dryspin® flange lead screw nut with flat, high helix thread, JFRM	DST-JFRM-252525DS10X25	1	22.85	22.85	Igus
**Trapezoidal spindles**					
Trapezoidal lead screw nut 8 × 1,5 R red bronze with aluminium housing		3	18.43	55.30	Motedis
Trapezoidal threaded spindle RPTS right TR 8 × 1,5 - L = 300 mm		2	3.15	6.30	Motedis
Trapezoidal threaded spindle 300X8 mm brass + lead screw	RBS10527	2	7.45	14.90	Roboter-Bausatz
**Motors**					
Wantai NEMA 17 stepper motor 40 mm 1.7A 42BYGHW609X1	RBS11814	5	13.15	65.75	Roboter-Bausatz
NEMA 23 stepper motor WT57STH115-4204A	RBS11137	2	31.95	63.90	Roboter-Bausatz
Shafts clutch flexible Mot D20L25, 5 / 8 mm		2	5.16	12.59	Motedis
Shafts clutch flexible Mot D20L25, 5 / 10 mm		2	5.16	12.59	Motedis
Shafts clutch rigid Mot D20L35, 8 / 8 mm		2	5.16	12.59	Motedis
CNC Motor angle for NEMA 17 steel	RBS10382	5	1.60	6.40	Roboter-Bausatz
Plate engine mount NEMA 23, t = 3 mm, Laser cut		2	1.95	3.59	Motedis
Plate engine mount NEMA 17, Laser cut, t = 3 mm		1	1.43	1.43	Motedis
**Electronics**					
Control Cabinet Rittal AX 1038.000	2251416–62	1	86.11	86.11	Conrad
Wantai Netzteil 24 V 14.6 A 350Wf¨ur 3D-Drucker / CNCMaschinen	RBS11135	1	37.95	37.95	Roboter-Shop
installation terminal block	730681–62	3	1.67	5.01	Conrad
Bridge	774915–62	2	0.46	0.92	Conrad
Mean Well MDR-60–12 12 V Top hat rail power supply	1297378–62	1	21	21	
DELIXI Solid State Relay CDG1-1DA / 480 V AC 40 A SSR	RBS13094	1	13.95	13.95	Roboter-Shop
door contact	700918–62	1	16.97	16.97	Conrad
Top hat rail	545703–62	1	10.92	10.92	Conrad
RJ11 cable CAN-Bus	2335606–62	1	7.13	7.13	Conrad
WAGO 221–415-1 221 connecting clamp	1188438–62	2	0.92	1.84	Conrad
Wire blue 2.5 mm^2^ 5 *m*	606297–62	5	1.83	9.15	Conrad
Wire black 2.5 mm^2^ 5 *m*	606297–62	5	1.83	9.15	Conrad
Wire ground 2.5 mm^2^ 5 *m*	606297–62	5	1.83	9.15	Conrad
Wire red 2.5 mm^2^ 5 *m*	606297–62	5	1.83	9.15	Conrad
Wire white 2.5 mm^2^ 5 *m*	606297–62	5	1.83	9.15	Conrad
Wire brown 2.5 mm^2^ 5 *m*	606297–62	5	1.83	9.15	Conrad
Wire green 2.5 mm^2^ 5 *m*	606297–62	5	1.83	9.15	Conrad
cable gland M20	532212–62	2	0.55	1.10	Conrad
RS PRO Heizmatte, Rechteckig 100 W + 300 °C, 150 × 200 mm, 240 V AC	245–635	1	66.70	66.70	RS-online
**Controller boards**					
Duet 3 Mainboard 6HC	Duet3MB6HC	1	259.90	259.90	Dold Mechatronik
Duet 3 Expansion 3HC	Duet3EB3HC	1	119.90	119.90	Dold Mechatronik
UNO *R*3 MEGA328P ATMEGA16U2 Board Arduino kompatibel	RBS10911	1	8.45	8.45	Roboter-Shop
**Sensors**					
Load Cell / Wheatstone Amplifier Shield (2ch)	RBC-Onl-38	1	22.42	22.42	robotshop
Antclabs BL-Touch Smart V3.1	RBS11287	1	39.95	39.95	Roboter-Shop
load cell DYMH-106	EAN 9399415451073	1	35.49	35.49	Amazon
Thermistor NTC 3950 100kΩ	RBS10126	1	0.95	0.95	Roboter-Shop
mechanical Endstop with cable	RBS11170	1	4.45	4.45	Roboter-Shop
**3D-printer parts**					
E3D V6 Full Kit 1.75 mm Universal Bowden 12 V	RBS13364	1	67.95	67.95	Roboter-Shop
metal bowden extruder	RBS11189	1	15.26	15.26	Roboter-Shop
Bowden Tube 1 *m*	RBS12425	1	1.21	1.21	Roboter-Shop
**Miscellaneous**					
Kapton tape	RBS10602	1	2.65	2.65	Roboter-Shop
Laibungsplatten 500 × 250 mm, 15 mm Stärke vorgrundiert	KP02.LP01. 15.0001–00	1	7.81	7.81	kalziumsilikatplatten.com

## Build instructions

### Frame

At first the bottom part of the frame is built. Following items are needed:

• two pieces of 300 mm long 45 × 45L profiles
• two pieces of 400 mm long 45 × 45L profiles
• eight *T*-nuts slot 10
• eight screws M8 × 16
• four brackets 45B-Type slot 10

**Fig. 2:**
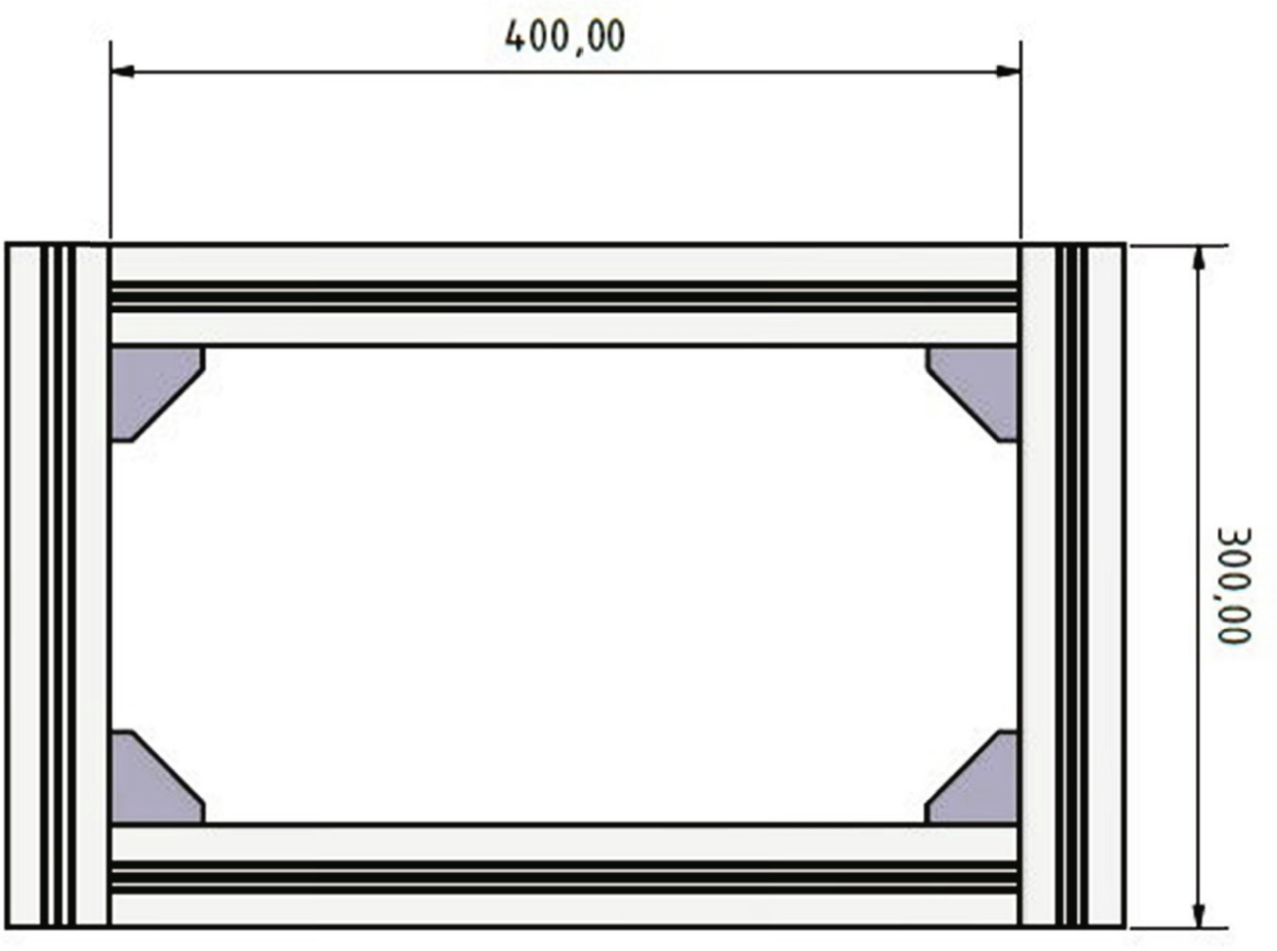
Front view of the bottom part of the frame.

The profiles form a rectangle and the 300 mm long profiles are mounted flush on the front side of the 400 mm long profiles. Fig. 2 shows the mounted bottom part of the frame. To connect the profiles with each other 90-degree angle brackets, screws and *T*-nuts are used.

**Fig. 3:**
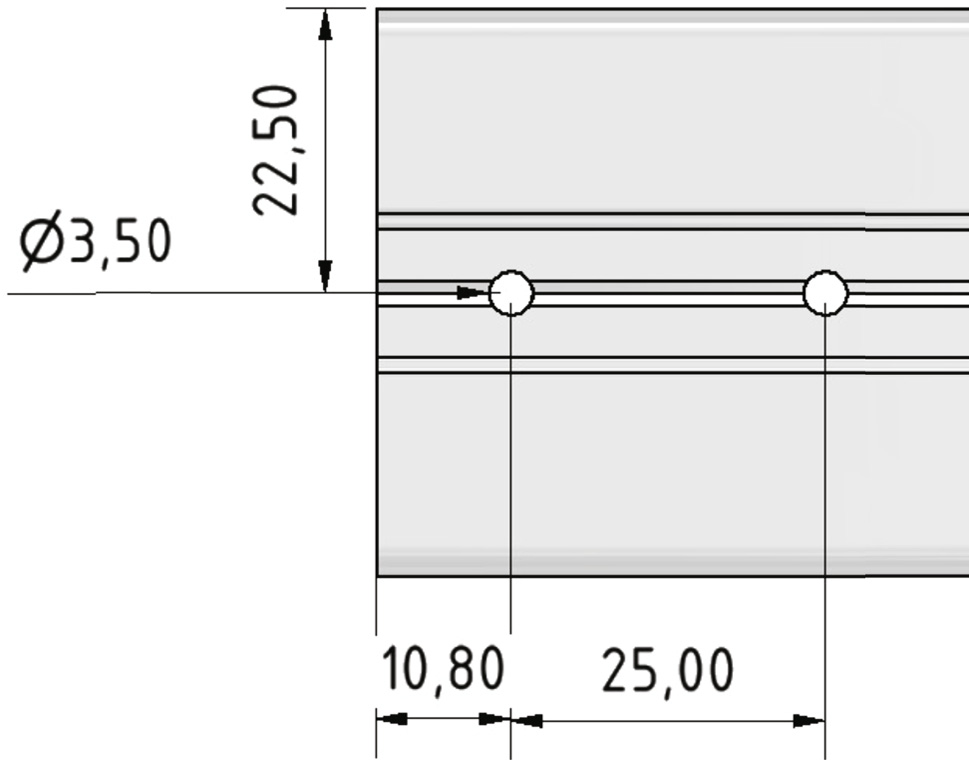
Position and dimension from two of the twelve holes drilled into the 985 mm long profiles. All following holes are drilled in 25 mm distance to the previous one.

Now twelve holes must be drilled through the 985 mm long profiles which will later be used for mounting the linear guiding rails. Fig. 3 shows the right position and dimension.

The next step is mounting the upper part of the frame which requires the items listed below:

• one piece of 300 mm long 45 × 45L profile
• two pieces of 985 mm long 45 × 45L profiles
• six brackets 45B-Type slot 10
• twelve *T*-nuts slot 10
• twelve screws M8 × 16

**Fig. 4:**
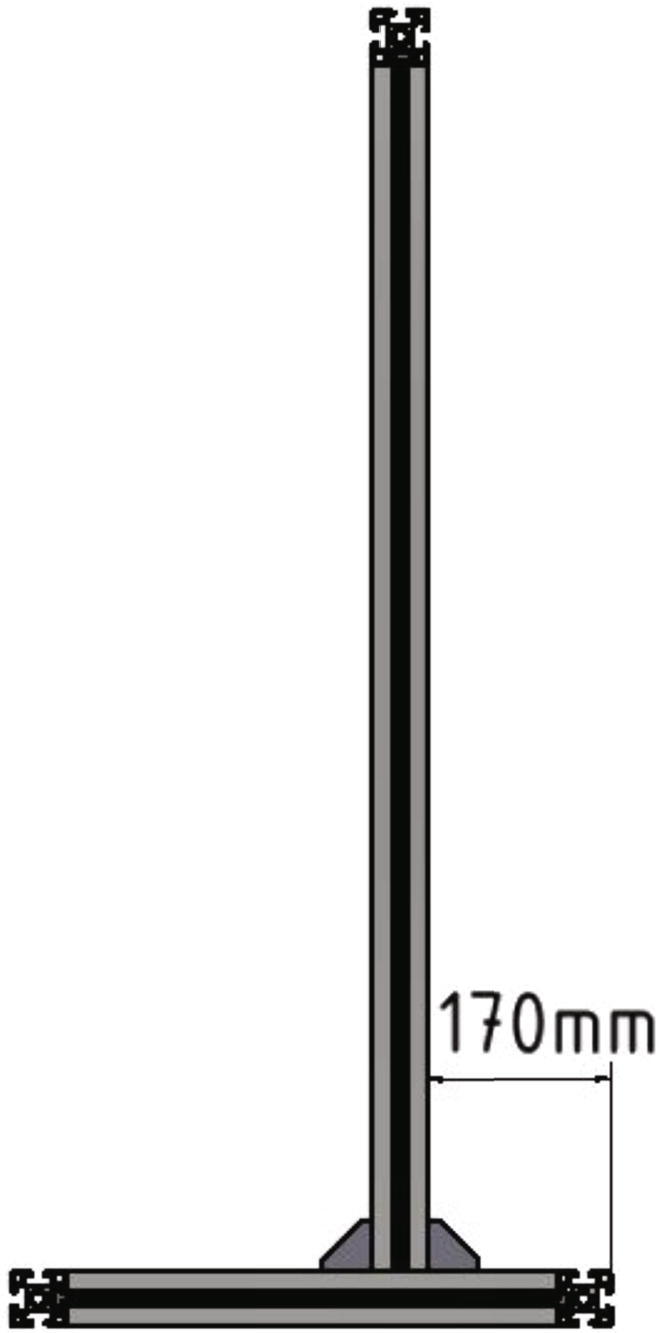
Front view of the mounted upper frame.

The 985 mm long 45 × 45L profiles are placed on the 400 mm long profiles of the bottom part of the frame with a distance of 170 mm from the outer edge (see also Fig. 4). Mind that the holes in the two 985 mm profiles must be at the top. On the top front side of the two 985 mm profiles the remaining 300 mm profile is mounted. All profiles are connected with 90-degree angle brackets, *T*-nuts and screws.

**Fig. 5:**
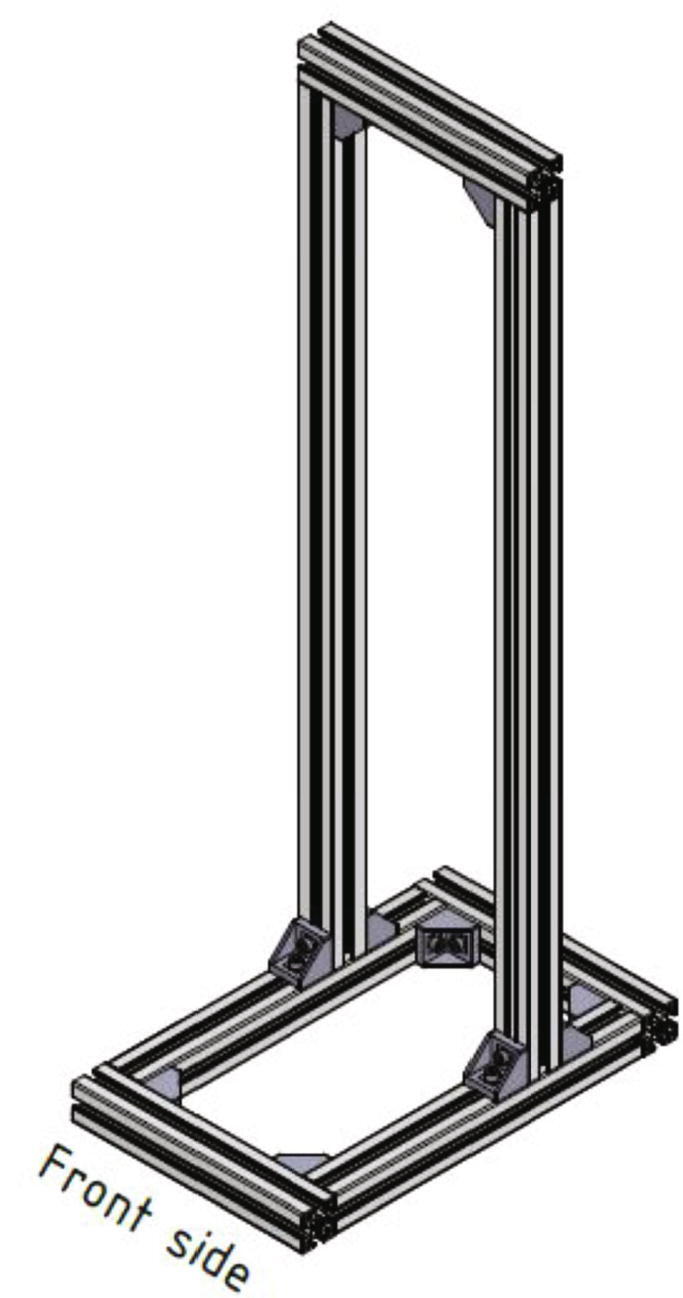
Angle view of the completed frame. The 300 mm long profile with the greater distance to the 985 mm long profiles is defined as the front side.

Fig. 5 shows the frame after connecting all profiles together.

At last the two linear guidings will be screwed to the frame. For this the following parts are needed:

• two linear guide rails Miniature MR12M-N, L = 300 mm
• 23 screws M3 × 45
• 23 nuts M3

**Fig. 6:**
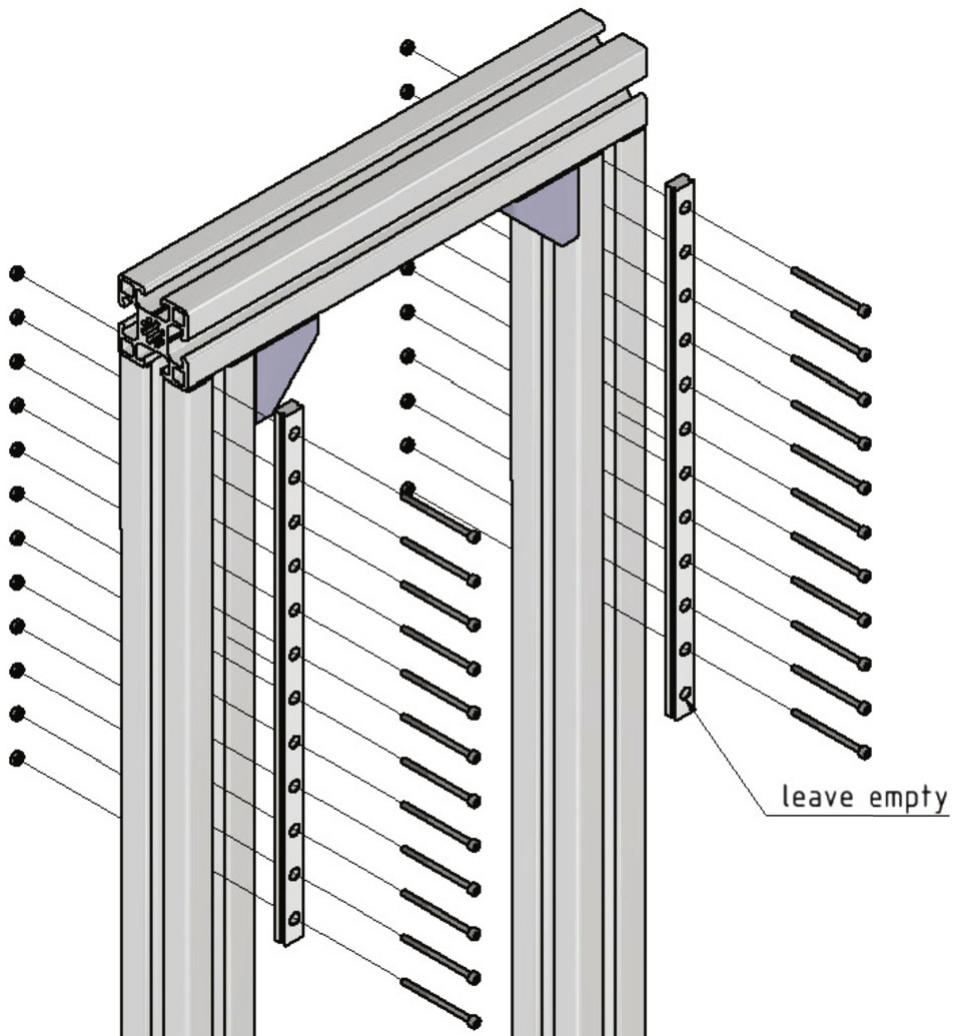
Mounting of the linear guides. The marked hole stays empty in this step.

The linear guide rails are screwed onto the 985 mm profiles on the backside of the frame. For this the screws are inserted through the holes in the profile and secured with nuts. The hole at the lower end of the right linear guide stays empty. It will later be used to mount an endstop. Fig. 6 shows how to connect the linear guides with the profiles.

The frame is designed for maximum rigidity with regard to the forces acting in the measuring unit. Since the measuring cell only applies forces along the vertical axis, the test unit can be held in a simple gantry design. In addition, the use of profiles allows quick assembly and adaptation to further measuring tasks.

### Measuring unit

In this section the measurement unit will be mounted and connected to the frame. At first two slides for the linear guides are getting screwed to the crossbar. The items needed for this step are listed below:

• one crossbar (Part no. 7)
• two slides MR12MN-SS
• 8 screws M3 × 10

**Fig. 7:**
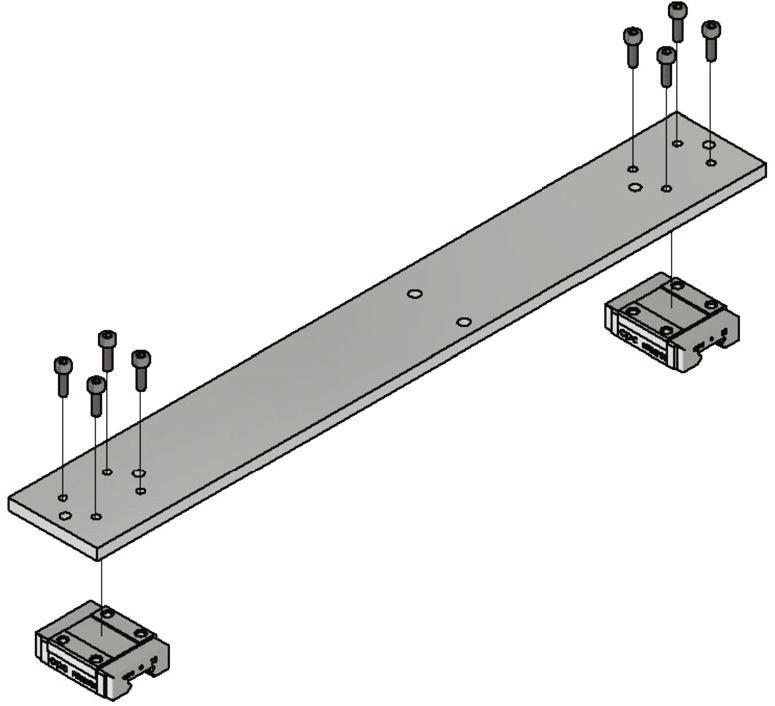
View of the mounted linear guide sledges.

The slides are screwed to the crossbar as shown in Fig. 7.

Now the lead screws for the trapezoidal spindles getting mounted. The required items are:

• two Trapezoidal lead screw nuts 8 × 1,5 R red bronze with aluminum housing
• four screws M4 × 40
• four nuts M4

**Fig. 8:**
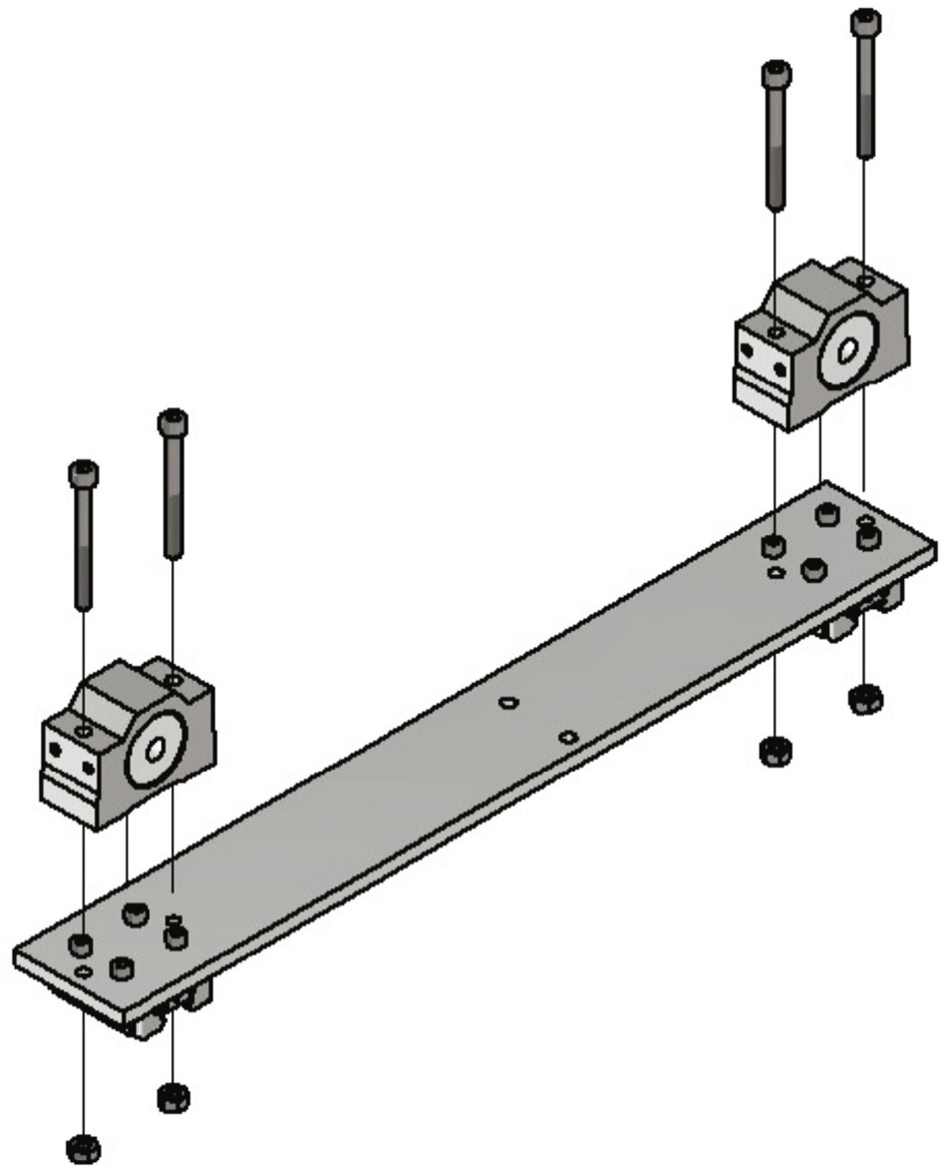
Mounting lead screws.

The M4 × 40 screws are put through the crossbar and the aluminum housing of the lead screws and secured with M4 nuts. Fig. 8 shows this step.

Now the load cell will be mounted to the crossbar. Following parts are needed:

• one load cell
• one adapter crossbar to load cell (Part no. 1)
• one adapter load cell (Part no. 2)
• two screws M4 × 20
• two nuts M4

**Fig. 9:**
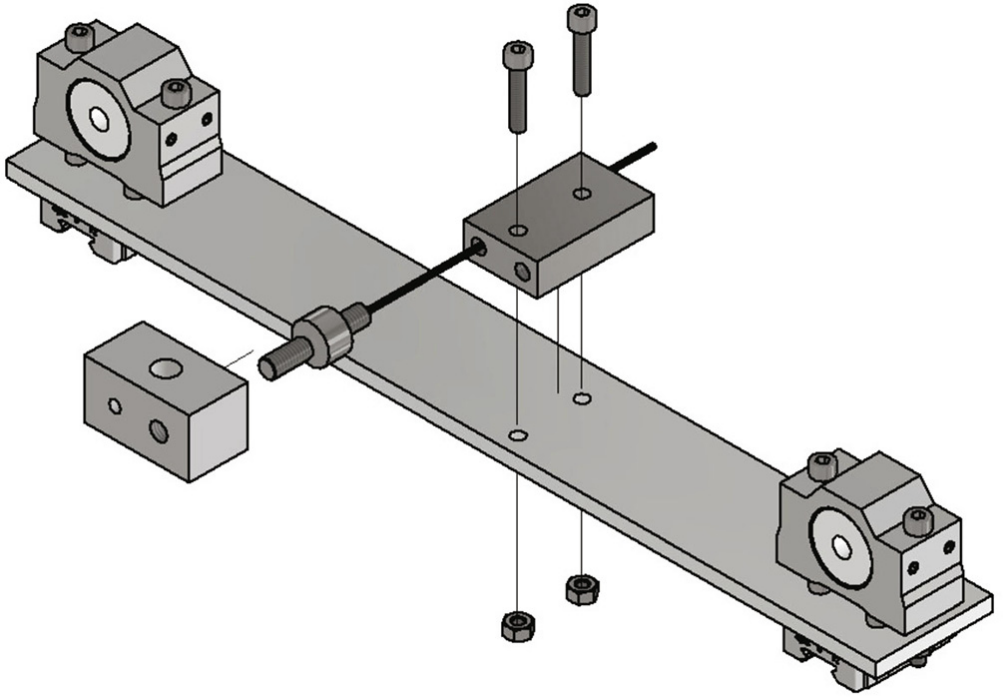
Mounting crossbar.

The adapter crossbar to load cell is connected to the crossbar with two M4 × 20 screws and two nuts M4. Next the wire of the load cell is put through the M6-thread of the adapter crossbar to load cell and second the load cell is screwed in. After that the adapter load cell is screwed to the second thread of the load cell (see Fig. 9).

Now the NEMA 23 stepper motors will be attached to the frame. For this the parts listed below are required:

• two NEMA 23 stepper motors
• two plate engine mounts NEMA23
• four *T*-nuts slot 10
• four screws M8 × 16
• eight screws M5 × 16
• eight nuts M5

**Fig. 10:**
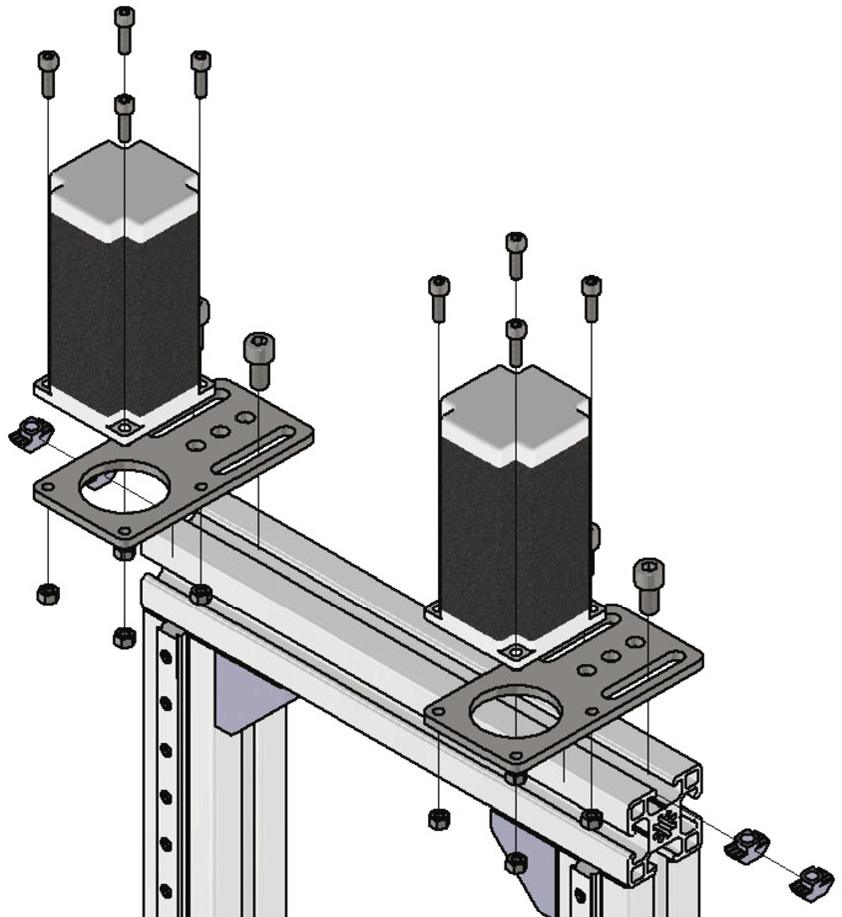
Mounting NEMA 23 stepper motors.

The two motor plates are attached on the top side of the frame facing towards the linear guide rail with two *T*-nuts and M8 × 16 screws each. They should not be tightened because later on the motors must be aligned to the crossbar. After mounting the motor plates to the frame, the NEMA 23 stepper motors are connected to the motor plates with four M5 × 16 screws and four nuts M5 each. Fig. 10 shows how the motors should be attached.

Next the trapezoidal spindles will be attached to the NEMA 23 stepper motors. Following parts are necessary for this step:

• two trapezoidal threaded spindle T8 × 1,5 300 mm
• two shafts clutch rigid Mot D20L35, 8 / 8 mm with screws

**Fig. 11:**
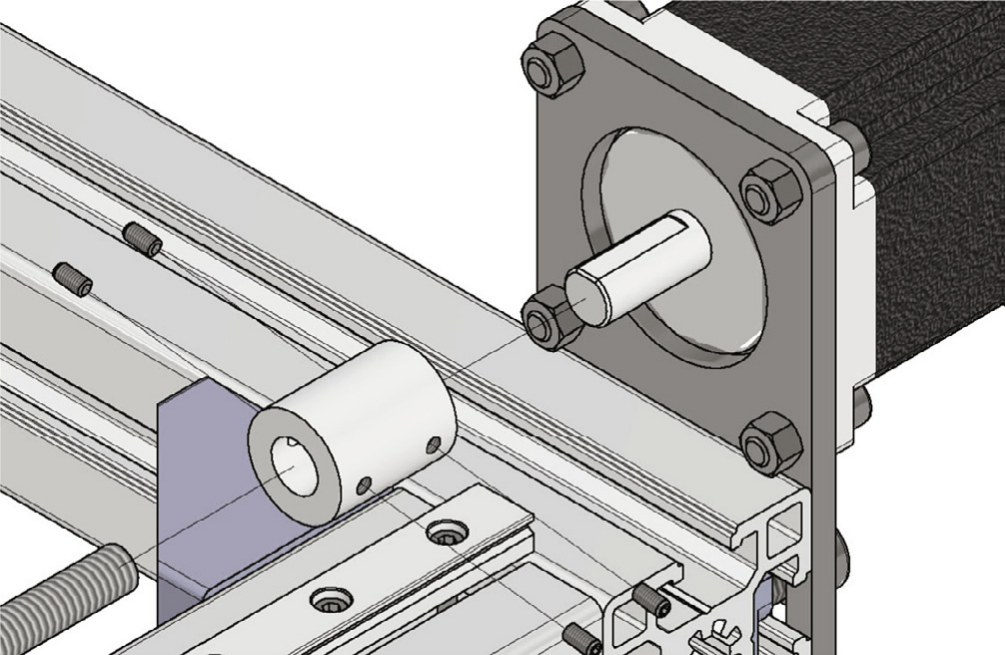
Mounting spindle.

Put the shafts clutch on the motor shafts and secure each of them with two screws. After that put the spindles in the mounted clutch and secure each of them also with two screws. Fig. 11 shows the mounting of one of the spindles.

**Fig. 12:**
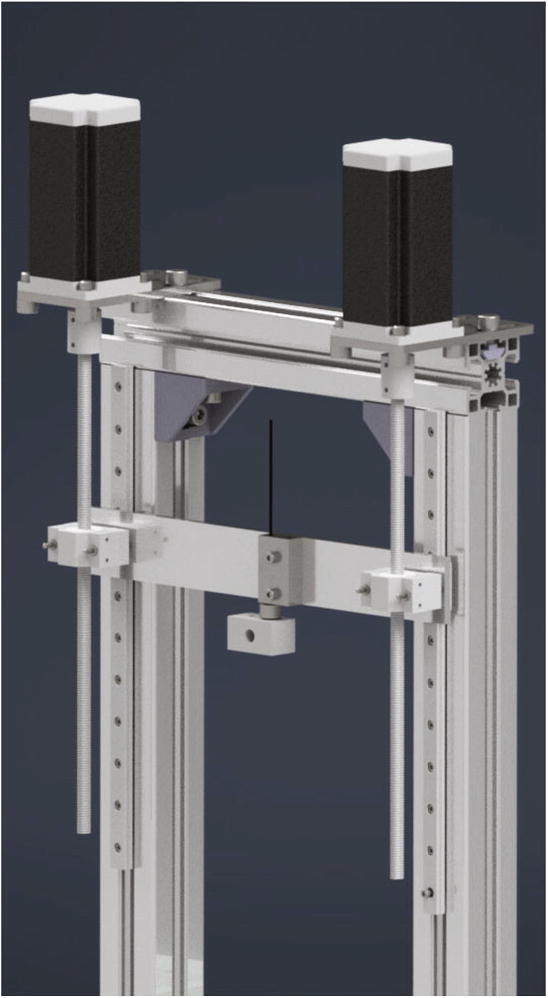
View of the crossbar connected to the spindles and linear guides.

The mounted crossbar gets now pushed onto linear guides while the spindles are screwed into the lead screw nuts. For this the motor plates must be aligned correct to the lead screw nuts. After mounting the crossbar correct to the linear guides, the screws holding the motor plates can be tightened. If the motors and the lead screw nuts are correctly aligned, the crossbar should move without effort from one end of the linear guides to the other (see Fig. 12).

After mounting the crossbar, the hook will be attached to the load cell. For this step the following is needed:

• wire 1.5 *m*
• one hook (Part no. 15)

The wire is knotted to the adapter-load-cell-to-hook and the hook. Due to the DIN EN 28510-1 standard the distance between hook and adapter load cell (part 2) must be at least 600 mm. Now the mechanical endstop of the measuring unit will be placed. For this the below listed parts are required:

• one mechanical endstop
• one measuring unit mechanical endstop mount (Part No. 10)
• one screw M3 × 8
• two nuts M3
• one screw M3 × 50

**Fig. 13:**
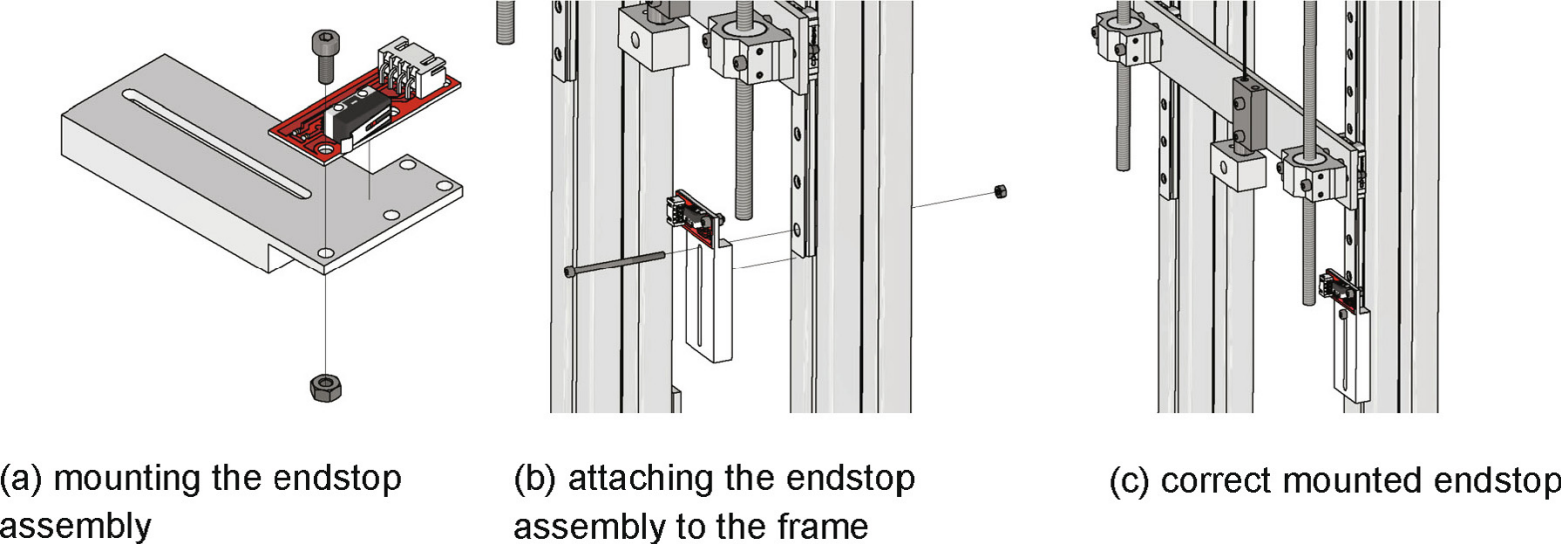
Mounting the mechanical endstop of the measuring unit.

At first the endstop is attached to the measuring unit mechanical endstop mount with a M3 × 8 screw and a nut M3 (see Fig. 13a). The measuring unit mechanical endstop mount is pushed on the right linear guide and placed at the lower end (see also Fig. 6b). Next it is mounted with a screw M3 × 50 and a nut M3. Fig. 13c) shows the correct mounted endstop.

The measuring unit uses a widespread type of load cells which are available for many measuring ranges. Due to the simple attachment of the load cell it can easily be replaced by an appropriate one if the observed forces are exceeding the measuring range. It is also possible to mount other types of force sensors like s-type load cells or capacitive sensor. The NEMA 23 stepper motors provide a torque up to 3 Nm so that the achievable forces are only limited by the clamp connection of spindle and motor shaft.

### MEX-system

In this section it will be explained how to build the MEX-system of the test device. It is constructed as a cartesian printer with a moving printing bed. At first the shafts which guide the build platform assembly will be attached. This requires following parts:

• four shaft supports SH12/SK12
• eight *T*-nuts slot 10
• eight screws M8 × 16

**Fig. 14:**
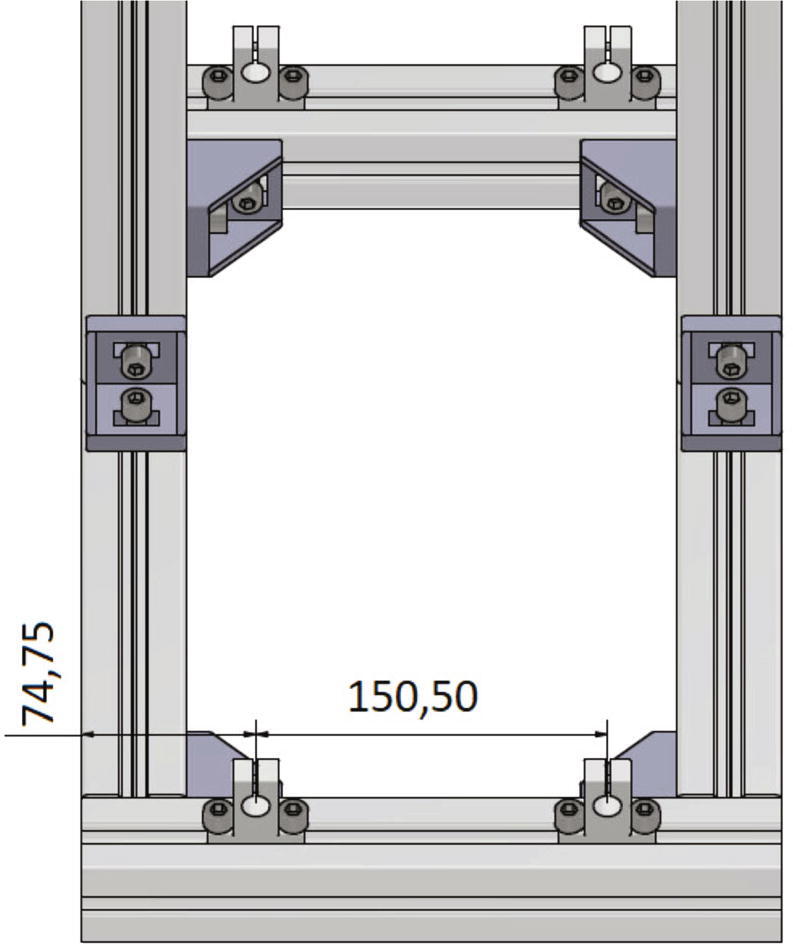
Front view of the correct mounted shaft supports for the y-axis shafts.

The shaft supports are placed at the bottom part of the frame on top of the 300 mm profiles and secured with *T*-nuts and M8 × 16 screws. The screws should not be tightened so that the opposing shaft supports can be aligned with each other. Fig. 14 shows the correct distances between the shaft supports.

In the next step the base plate will be mounted. The below listed items are necessary:

• one base plate (part no. 3)
• four linear bearings 12 mm SC-AJ12 SCJ12UU adjustable clearance
• one trapezoidal lead screw nut 8 × 1,5 R red bronze with aluminum housing
• one lead screw nut high helix thread DST-JSRM-2220DS10X25
• 18 countersunk screw M4 × 35
• 18 nuts M4

**Fig. 15:**
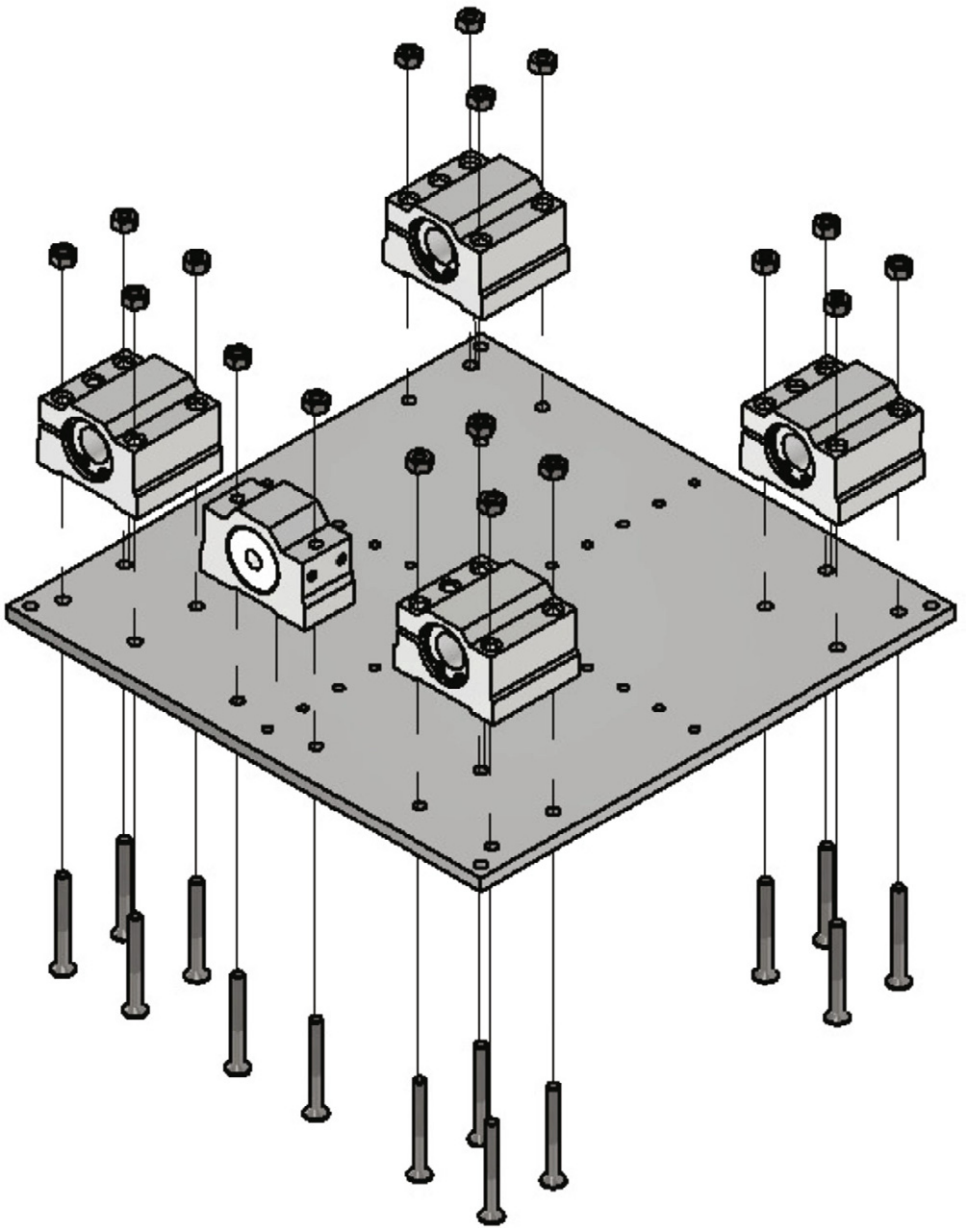
Mounting base plate.

At first the trapezoidal led screw nut must be removed from its housing and be replaced with the high helix threaded lead screw nut. Next in each corner of the base plate a linear bearing is mounted with four countersunk M4 × 35 screws and four nuts M4. The linear bearings must be aligned to each other. At one edge of the base plate the aluminum housing with the high helix lead screw nut is mounted with two countersunk screws M4 × 35 and corresponding nuts. Fig. 15 shows these steps.

**Fig. 16:**
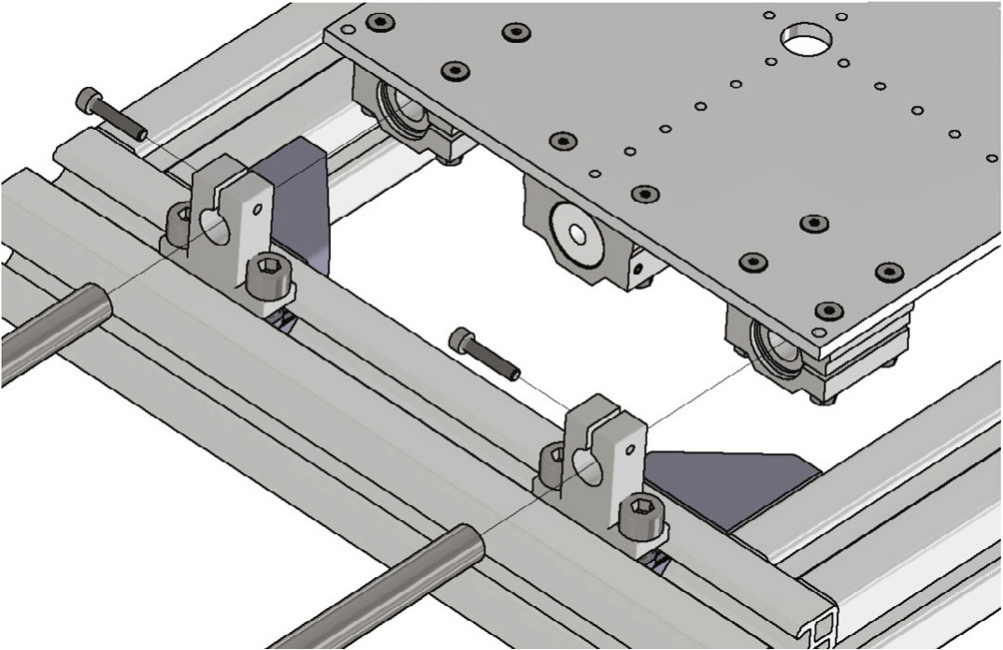
Mounting of the base plate to the frame.

The next step is attaching the base plate to the frame. This requires the two shafts with 12 mm diameter and 500 mm length. The shafts are put through the y-axis shaft supports and the linear bearings (see Fig. 16). The lead screw must be facing to the front side. After mounting the shafts, the base plate has to glide easily from one end of the shafts to the other. Is this fulfilled all screws holding the y-axis shaft mounts can be tightened.

**Fig. 17:**
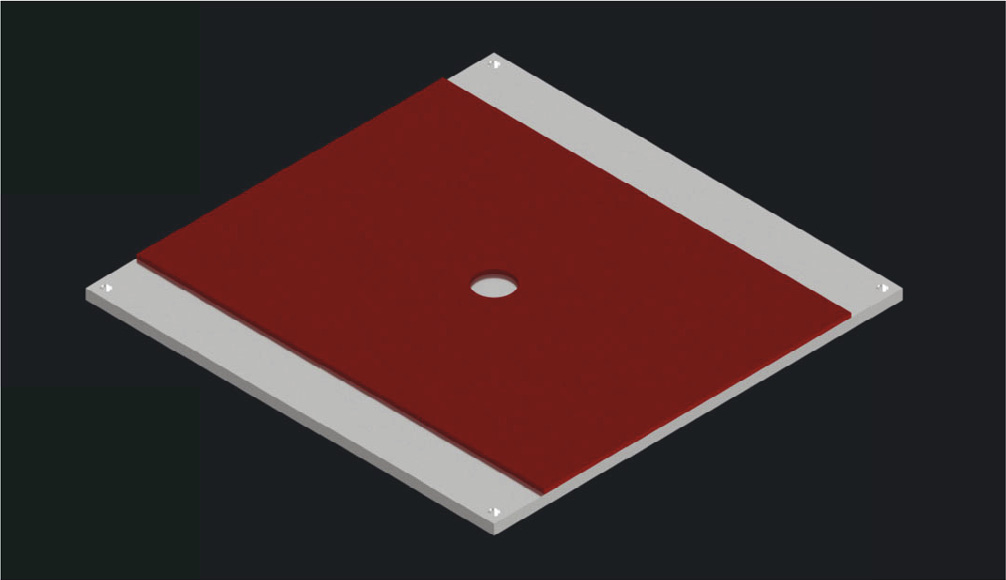
View of the silicon heat pad glued to the upper plate of the build platform.

Now the silicon heat pad with the article name “RS PRO Heizmatte” gets glued to the build platform top plate (part no. 5). The suggested heat pad is already coated with glue. Only the protective foil has to be removed and the heat pad can be pressed to the surface of the plate. It should be positioned as shown in Fig. 17.

Next the build platform assembly will be mounted. Following parts are necessary:

• build platform top plate (part no. 5) with the attached silicon heat pad
• one insulation (part no. 9)
• one thermistor
• one build platform bottom plate (part no. 4)
• four spacers (part no. 12)
• four build surface clamps (part no. 17)
• eight screws M3 × 16
• four screws M4 × 40
• eight nuts M4
• Kapton adhesive

**Fig. 18:**
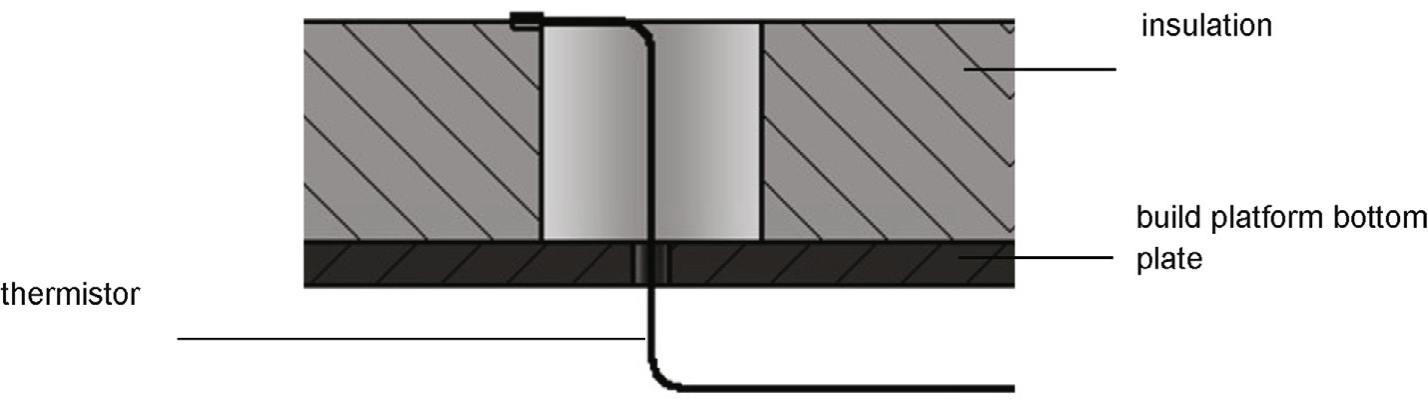
Placing of the thermistor regarding to the insulation and the build platform bottom plate.

The insulation (part no. 9) is put on top of the build platform bottom plate (part no.4) without further attachment. Next the wires of the thermistor are put through the holes in the middle of the insulation and the build platform bottom plate as shown in Fig. 18. The thermistor is secured with Kapton-adhesive.

**Fig. 19:**
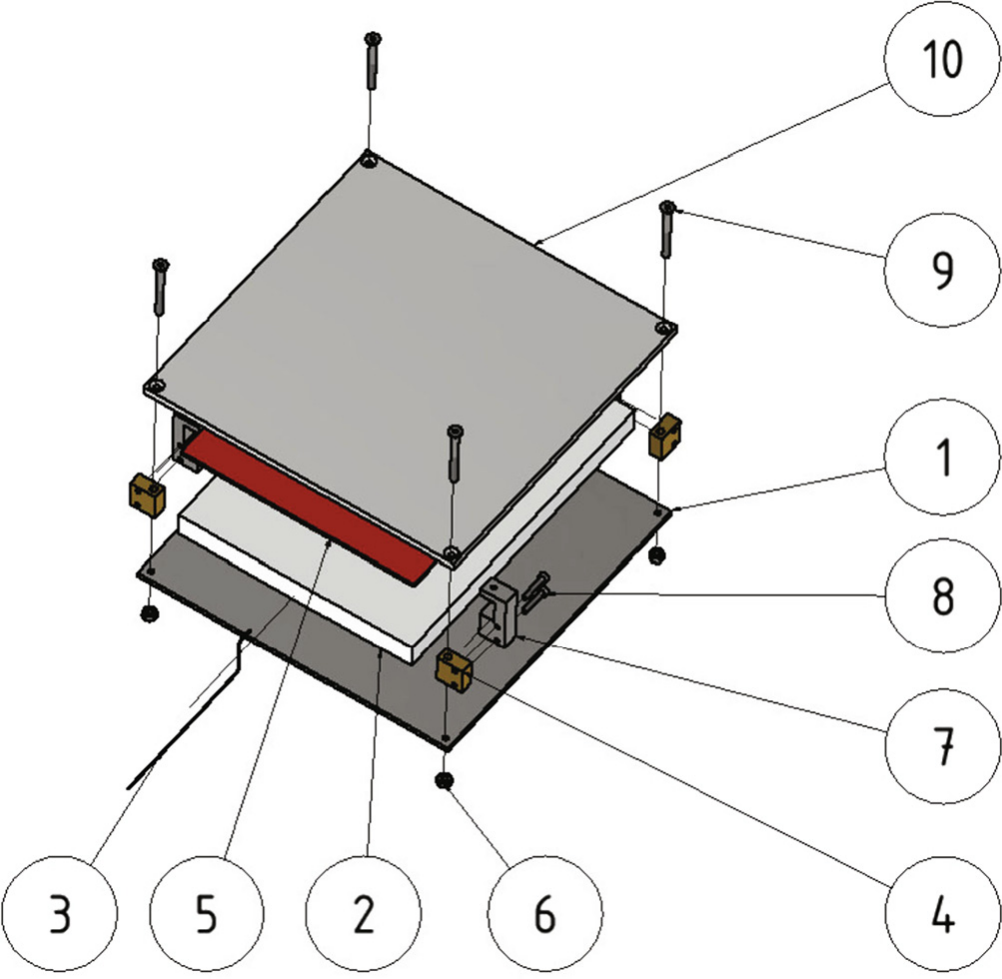
Explosion view of the build platform assembly. 1: build platform bottom plate; 2: insulation; 3: thermistor; 4: spacer; 5: silicon heat pad; 6: M4 nut; 7: build surface clamp; 8: screw M3 × 16; 9: screw M4 × 40; 10: build platform top plate.

On top of the insulation the silicon heat pad with the attached build platform top plate is placed. An explosion view of the assembly is shown in Fig. 19.

**Fig. 20:**
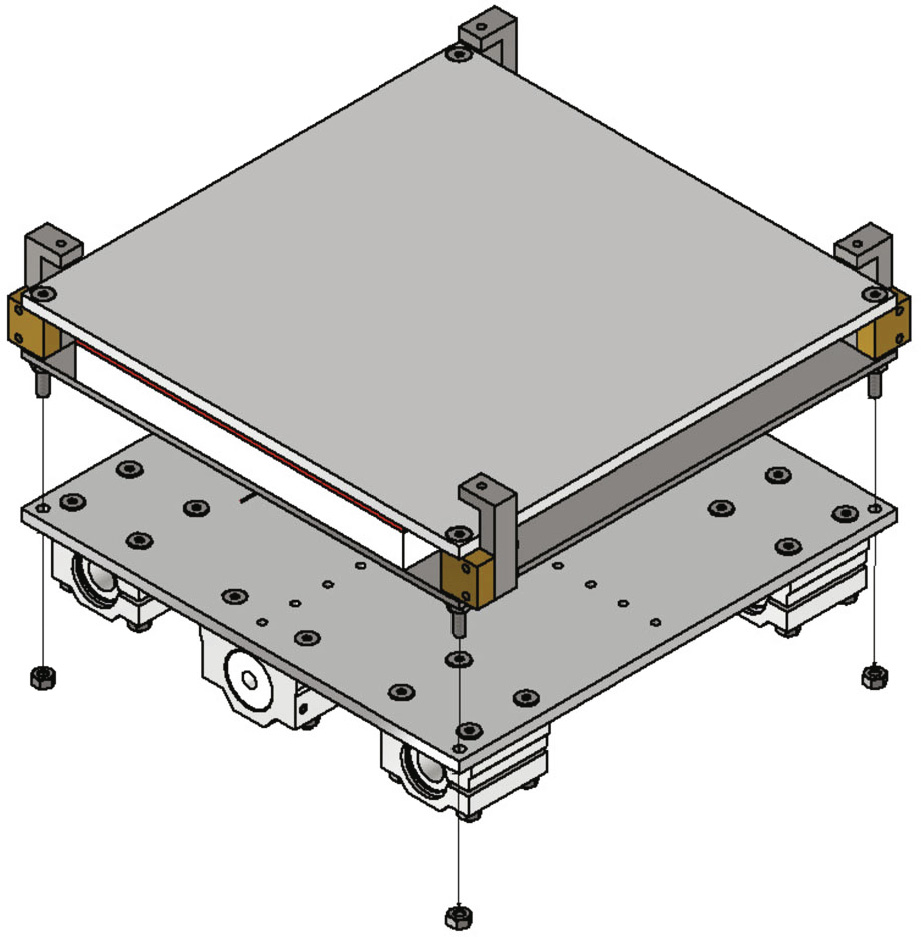
View of the build platform assembly.

The spacers (part no. 12) and the build surface clamps (part no. 17) are screwed together with two screws M3 × 16 as shown in Fig. 19 and then placed in the space between build platform top plate and build platform bottom plate. Correct alignment of the build surface clamps is important and must be done as shown in Fig. 20. Each of the spacers is secured with a M4 × 40 screw and a M4 nut. To mount the build platform assembly to the base plate the thermistor wires are put through the center hole of the base plate at first. Then the assembly gets screwed to the base plate with four nuts M4 as shown in Fig. 20.

Now the NEMA 17 stepper motor for the y-axis will be mounted. For this the below listed items will be required:

• one plate engine mount NEMA 17
• one NEMA 17 stepper motor
• one shafts clutch flexible 5/10 mm
• one high helix thread lead screw nut DS10X25 300 mm long
• four grub screws M4 × 6
• four screws M3 × 10
• two *T*-nuts slot 10
• two screws M8 × 16

**Fig. 21:**
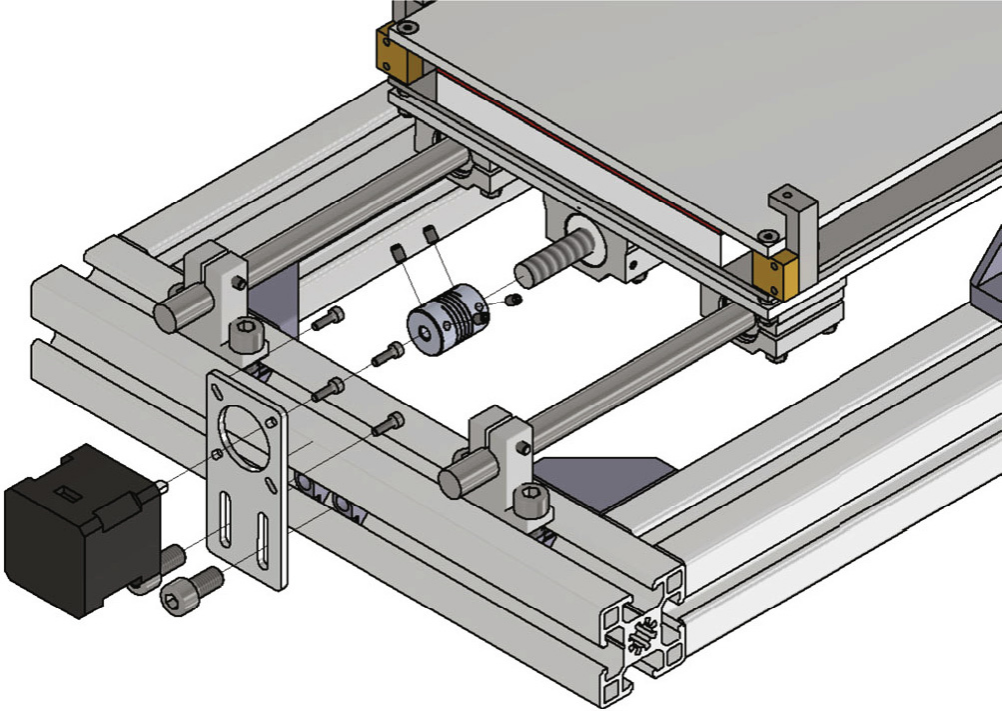
Installation of the y-axis stepper motor and spindle.

The plate engine mount NEMA 17 is attached to the front side of the testing device with two *T*-nuts and two screws M8 × 16. The screws must not be tightened. After mounting the plate engine mount, the NEMA 17 motor gets screwed to it with four screws M3 × 10. Now the shafts clutch is clamped to the motor shaft with two grub screws. The high helix lead screw gets screwed through the lead screw nut at the bottom of the base plate and must be connected to the NEMA 17 motor via the shafts clutch. After clamping the stepper motor and the lead screw together all screws can be tightened. Fig. 21 shows the mounting of the stepper motor and lead screw.

Now the gantry-system for the x- and z-axis will be mounted. The z-axis consists of two identical assemblies which are built simultaneously as described in the following. The following components are needed:

• two CNC Motor angles for NEMA 17
• two z axis plates (part no. 14)
• four screws M4 × 6
• two linear guide rails Miniature MR12M-N, L = 300 mm
• two guide carriages MR12ML-SS-V0-N
• 24 screws M3 × 6

**Fig. 22:**
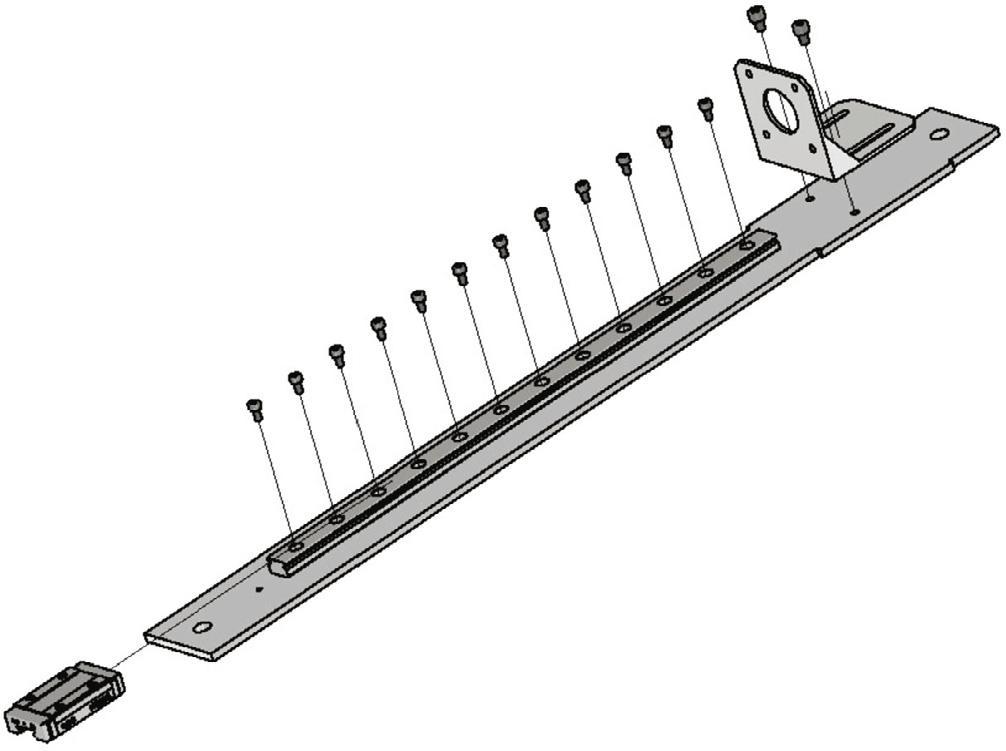
Mounting of the z-axis linear guide and the motor angle.

The NEMA 17 motor angle is screwed to the z axis plate as shown in Fig. 22. Next the linear guide rails with the carriages are screwed to the z-axis plate and should be aligned parallel to the edge of the plate.

In the following step the NEMA 17 stepper motors will be mounted to the NEMA 17 motor angles. The following parts are necessary:

• two NEMA 17 stepper motors
• two NEMA 17 shafts clutch flexible 5/8 mm
• eight screws M3 × 10
• eight grub screws M3 × 4

**Fig. 23:**
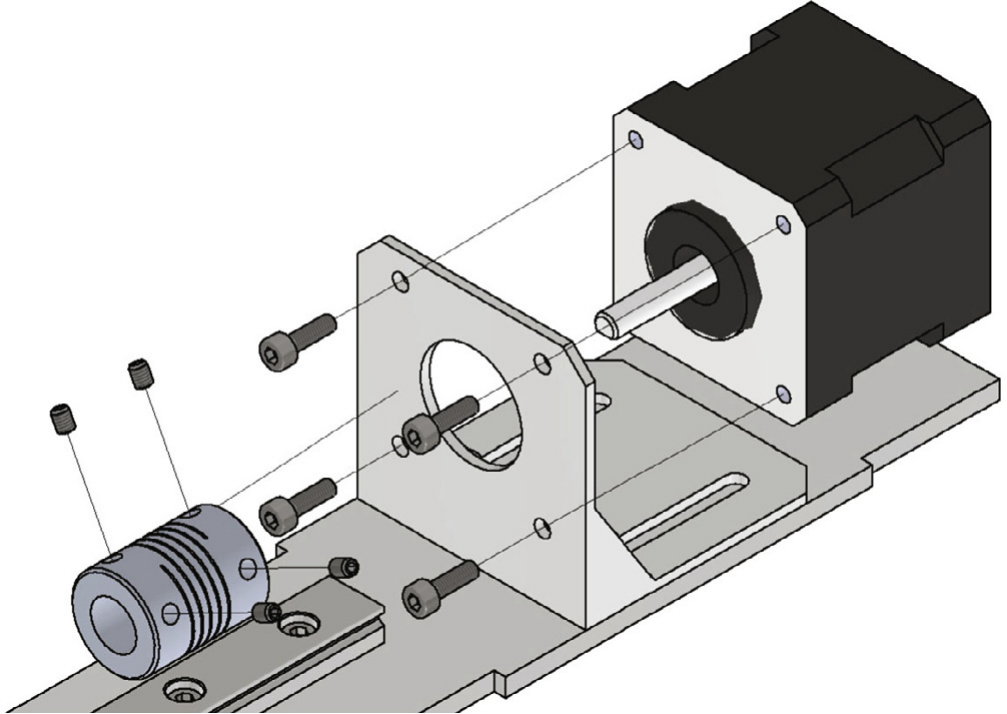
Mounting of the z-axis stepper motor with shafts clutches for the trapezoidal spindles.

The stepper motor gets attached to the motor angle with the M3 × 10 screws and the shafts clutches are clamped at the motor shaft with the grub screws (see Fig. 23).

Next the x-axis is assembled. The following items are required:

• one x axis plate (part no. 13)
• one Linear guide rail Miniature MR12M-N, L = 300 mm
• one guide carriage MR12MN-SS-V0-N
• one CNC Motor angle for NEMA 17
• 14 screws M3 × 6
• four washer M3

**Fig. 24:**
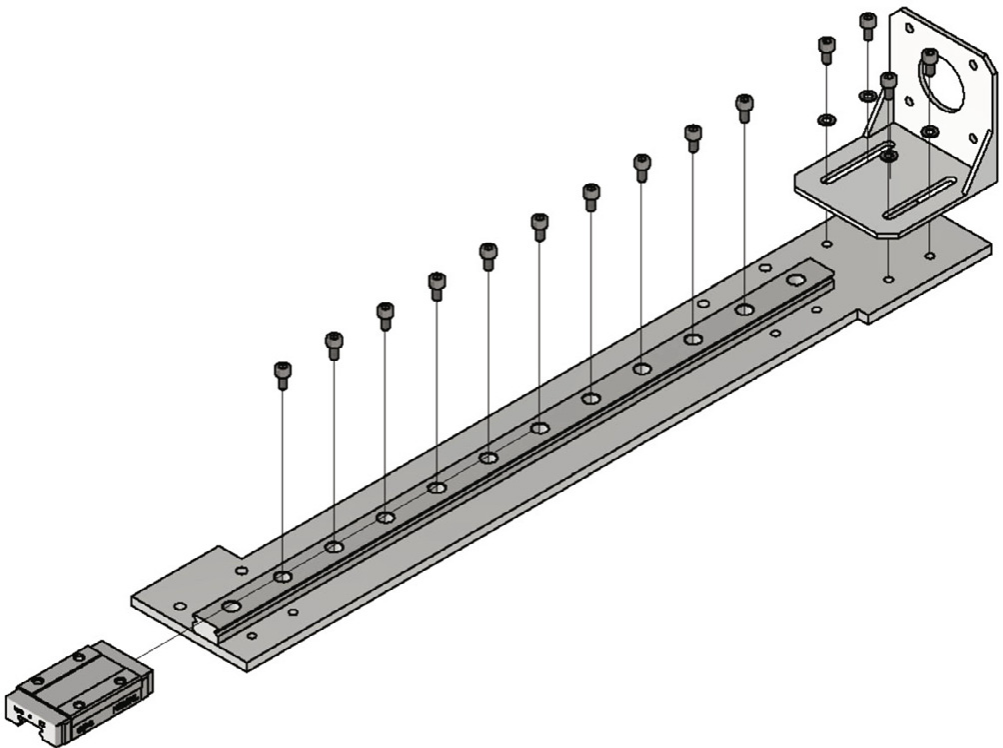
Mounting of the x-axis linear guide rail with carriage and the NEMA 17 motor angle.

The linear guide rail with carriage is mounted to the x axis plate with the M3 × 6 screws and must be aligned parallel to the edge of the x axis plate. Then the motor angle is attached to the x axis plate with the M3 × 6 screws and the washers as shown in Fig. 24.

Now the NEMA 17 stepper motor and the shafts clutch for the x-axis high helix lead screw will be installed. The below listed parts are necessary:

• one NEMA 17 stepper motor
• one shafts clutch 5/10 mm flexible
• four screws M3 × 10
• four grub screws M3 × 4

**Fig. 25:**
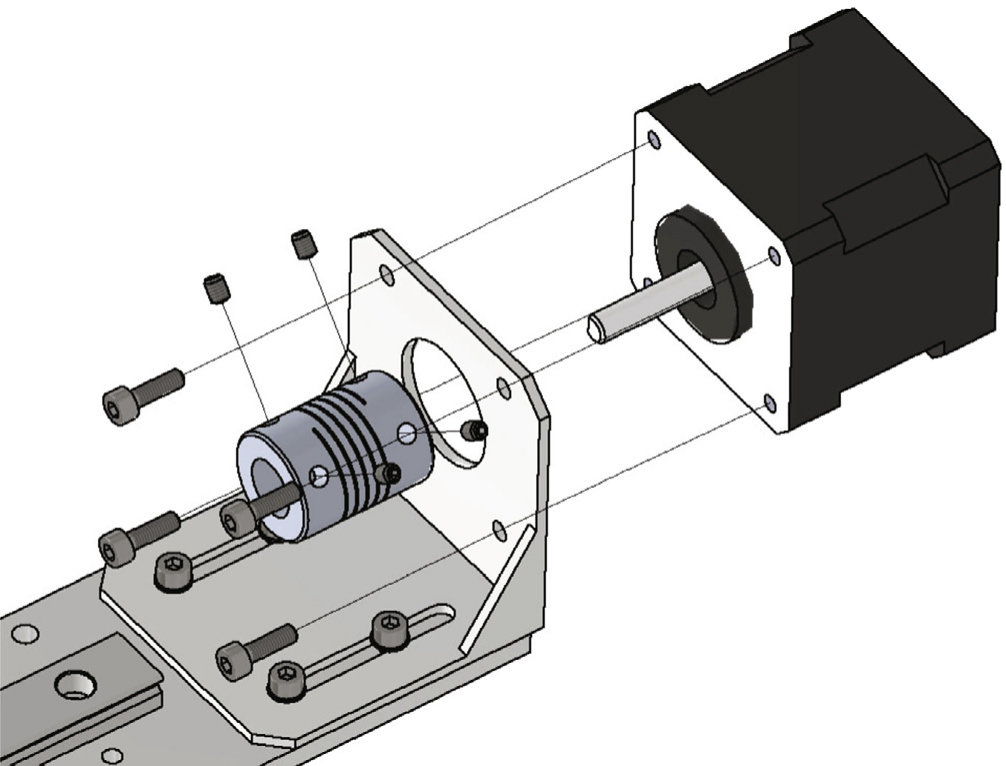
Mounting of the x-axis stepper motor and the shafts clutch.

The motor is attached with four screws M3 × 10 to the motor angle. At the motor shaft the clutch for the x-axis high helix lead screw is mounted with four grub screws. Fig. 25 shows the mounted parts.

The next step is the assembly of the x-axis carriage. All the listed items are needed:

• one x-axis carriage angle (part no.11)
• one connection × axis lead screw (part no. 6)
• one extrusion head connector (part no. 8)
• two screws M3 × 16
• two screws M3 × 6
• four countersunk screws M3 × 6

**Fig. 26:**
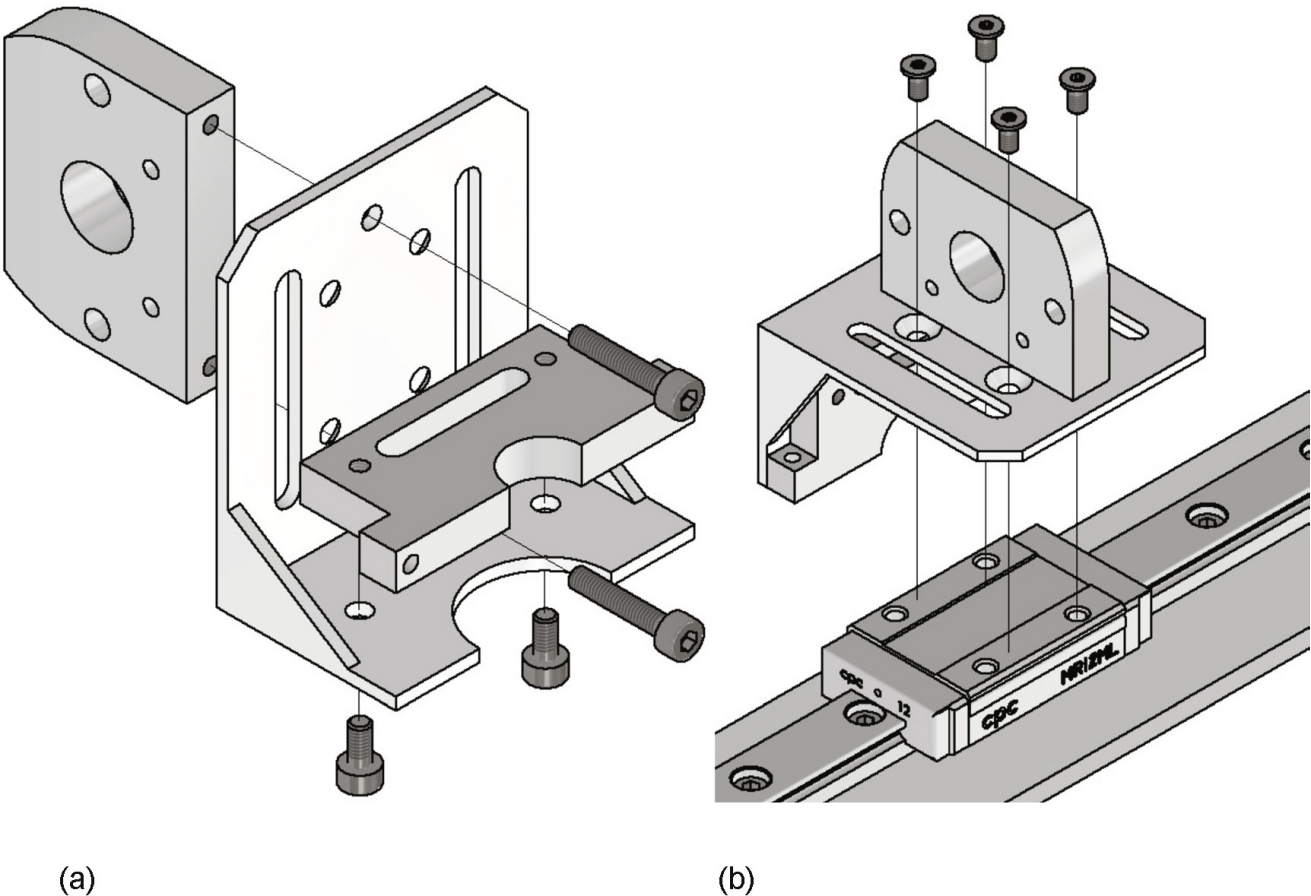
a) Mounting of the connection × axis lead screw and the extrusion head connector b) Mounting of the first part of the x-axis carriage to the x-axis assembly.

The connection x axis lead screw is mounted with two screws M3 × 16 to the x-axis carriage angle and the extrusion head connector is attached via two screws M3 × 6 to it as shown in Fig. 26a). Then the assembly is screwed to the carriage of the x-axis with four countersunk screws M3 × 6 (see 26b). Important is that the side of the x-axis carriage with the extrusion head connector must be under the x-axis guide rail.

Following the lead screw for the high helix spindle and the corresponding nut of the x-axis will be mounted with the following parts:

• one lead screw nut DST-JFRM-252525DS10X25
• one high helix thread lead screw DS10X25-R-ES 300 mm long
• two screws M5 × 16

**Fig. 27:**
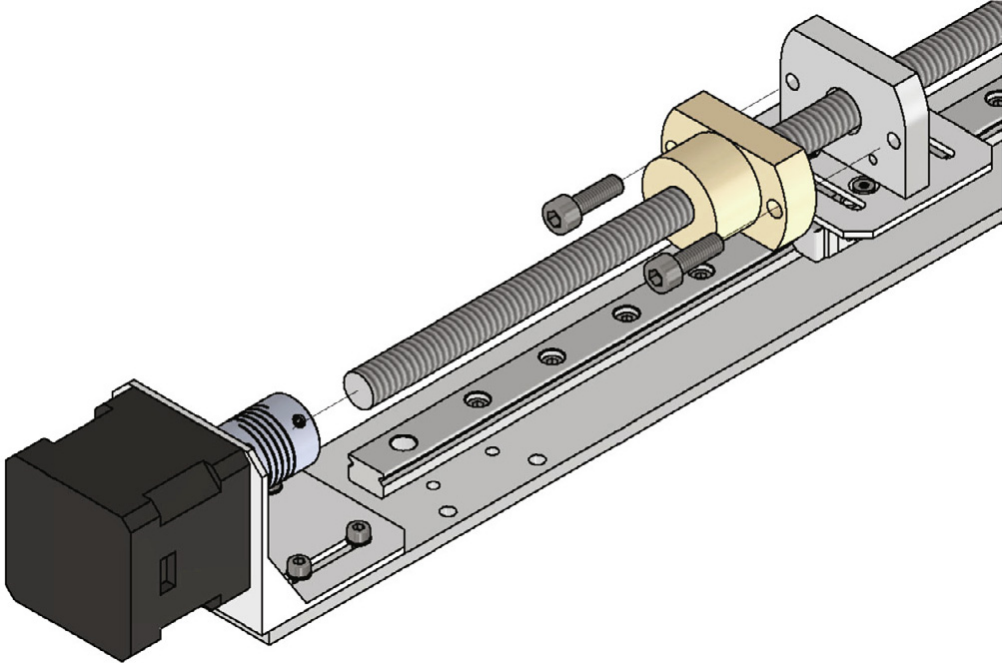
Mounting high helix thread lead screw and the corresponding nut.

The lead screw nut is attached with to screws M5 × 16 to the connection x axis lead screw facing towards the NEMA 17 stepper motor. Next the high helix lead screw is put through the nut and connected to the NEMA 17 stepper motor via the shafts clutch and two grub screws (see Fig. 27).

Now the lead screws for the z-axis spindles will be mounted. The following parts are necessary:

• two times Trapezoidal threaded spindle 300 X 8 mm brass + lead screw RBS10527
• two connection plate z axis (part no.18)
• four screws M5 × 40
• eight screws M3 × 12

**Fig. 28:**
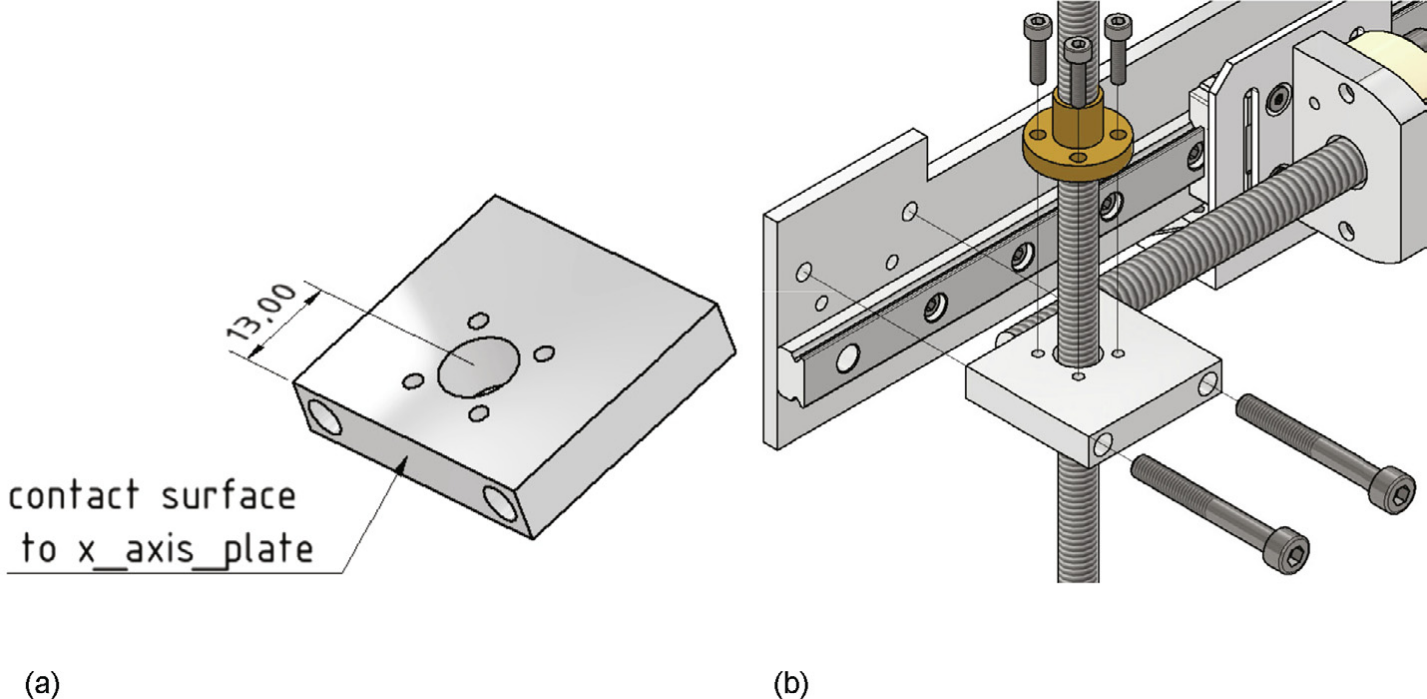
a) Correct alignment of the connection plate z-axis b) Mounted lead screws for the z-axis.

The connection plates z axis are attached to the x axis plate with two M5 × 40 screws each. Since the drilling for the z-axis spindle is not centric, care must be taken to ensure correct alignment. The distance between the center of the bore and the x-axis-plate must be 13 mm (see Fig. 28a). Now the lead screw nuts with the spindles already screwed in can be attached with four M3 × 12 screws each as shown in Fig. 28b).

Now the x-axis gets mounted to the two z-axis assemblies and the trapezoidal spindles will be clamped to the stepper motors. This requires following parts:

• eight screws M3 × 8

**Fig. 29:**
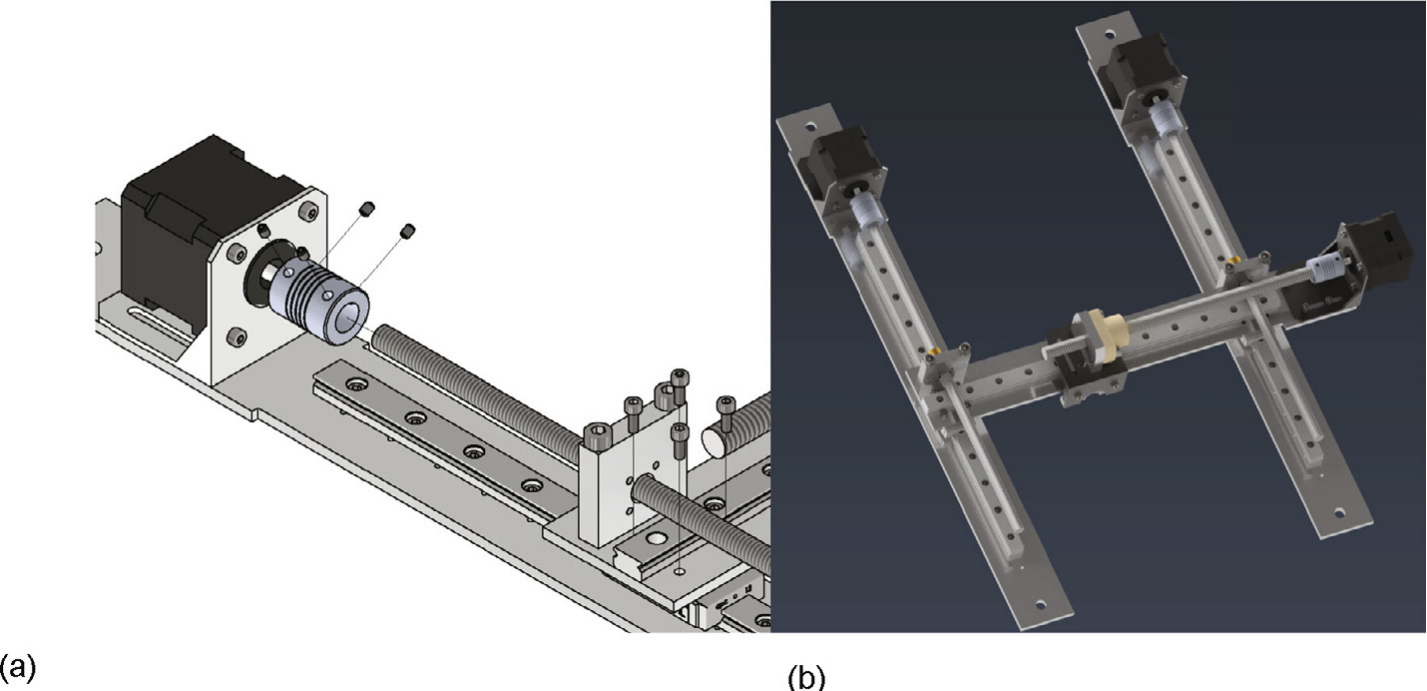
a) Mounting of the assemblies of the x- and the z-axis b) Fully mounted gantry system.

The x axis plate is screwed to the z-axis carriages with four screws M3 × 8 each as shown in Fig. 29a). In the next step the z-axis spindles will be installed. They get clamped to the z-axis NEMA 17 stepper motors with the clutch and the grub screws (see also Fig. 29a)). In the following the assembled x- and z- axis are called gantry system (see also Fig. 29b).

The gantry system gets now screwed to the frame. For this, following parts are required:

• four *T*-nuts slot 10
• four screws M8 × 16

**Fig. 30:**
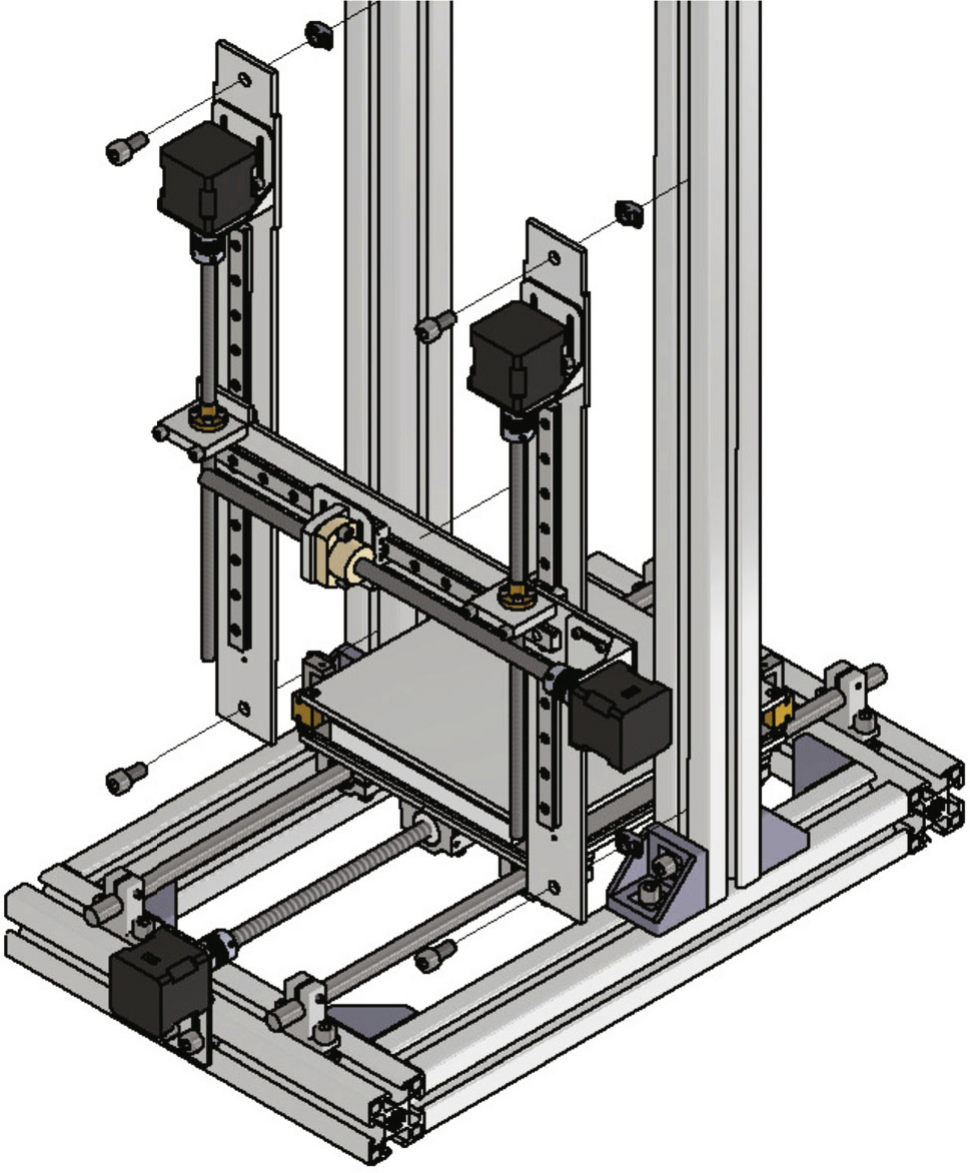
Mounting of the gantry system to the frame.

The gantry system is attached to the front-side of the frame with four screws M8 × 16 and four *T*-nuts placed in each corner as shown in Fig. 30. The bottom edge of the z axis plate should touch the bracket 45 B-Type slot 10 which connects the bottom and the upper part of the frame.

Next the extrusion head will be mounted to the x-axis carriage. At this point, an E3Dv6 extrusion head is installed, although any other extrusion head with a groove mount can also be used. The assembly of the extrusion head is not explained in this manual and reference is made to the documentation of the respective manufacturer. The below listed parts are necessary:

• one extrusion head connector (part no. 8)
• one extrusion head with groove mount e.g. E3D v6 - extrusion head
• two screws M3 × 12

**Fig. 31:**
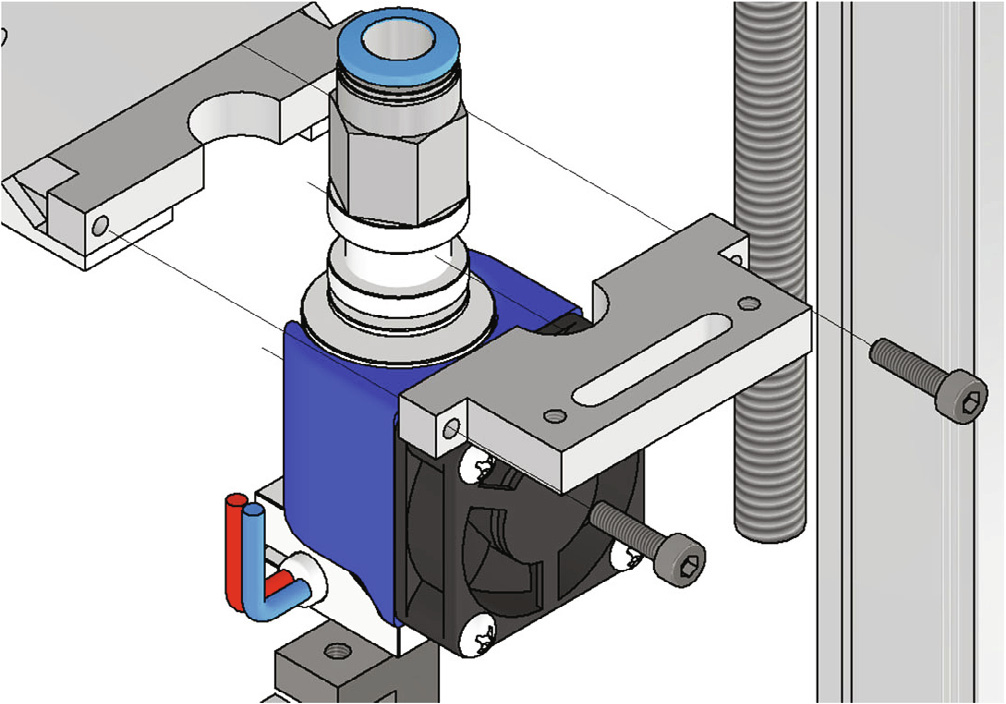
Mounting of the extrusion head to the x-axis carriage.

The extrusion head is clamped between the two extrusion head connectors which are screwed together with the two M3 × 12 screws (see Fig. 31).

Next the bed leveling sensor is mounted to the x-axis carriage. Following items are necessary:

• one z probe connector
• one BL-Touch
• two screws M3 × 12
• two screws M3 × 6

**Fig. 32:**
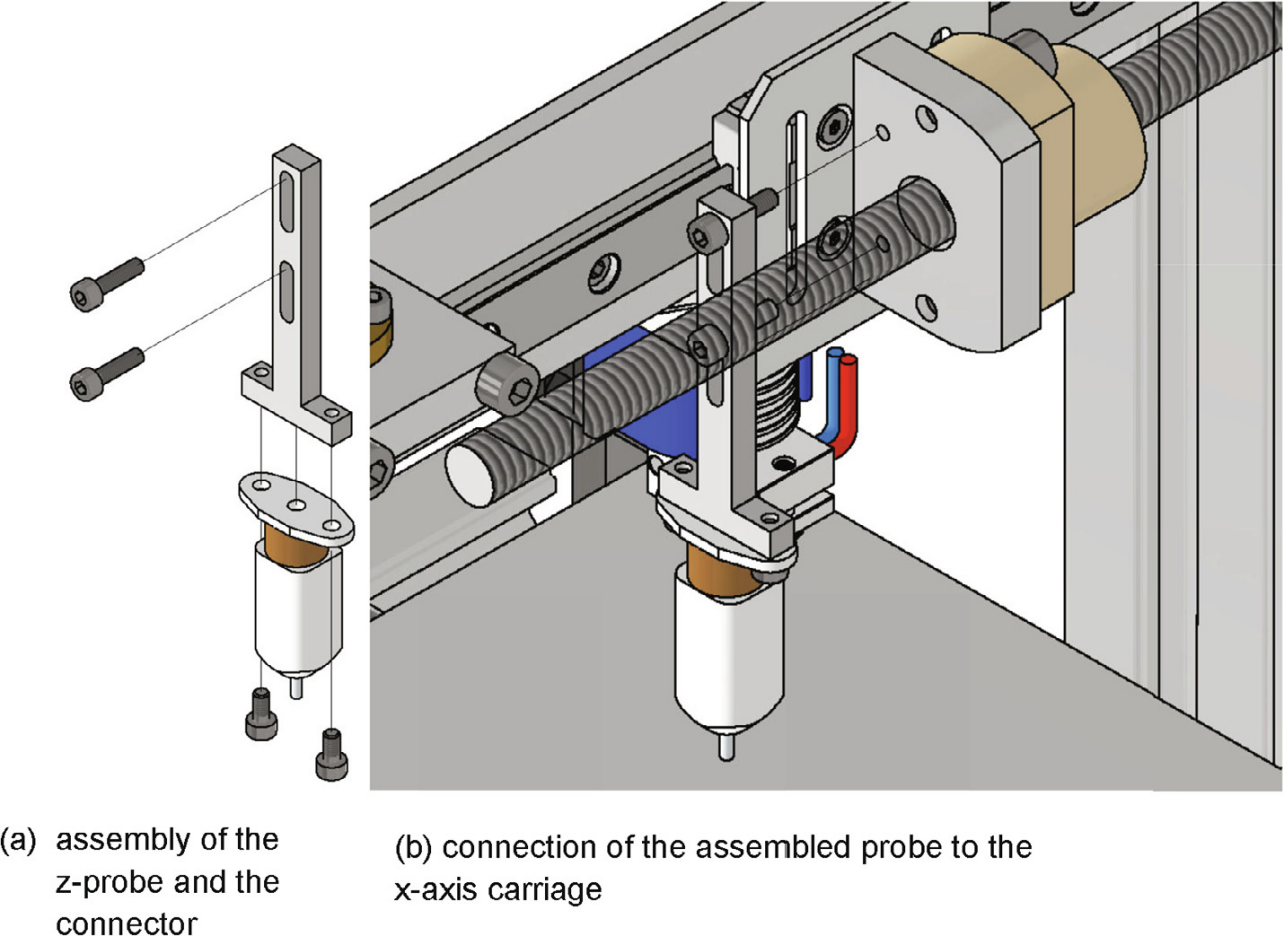
Mounting of the z-probe to the x-axis carriage.

The z-probe is attached to the z probe connector with two screws M3 × 6. This in turn is mounted to the x-axis carriage with two screws M3 × 12 as shown in Fig. 32. The mounting height will be adjusted later when the BL-Touch is getting calibrated.

With the setup presented here, any Bowden-extruder can be used. The assembly of the extruder is not explained here and reference is made to the documentation of the respective manufacturer. The extruder used here is described in more detail in [15]. For mounting the extruder from [15] to the frame following parts are necessary:

• one fully assembled extruder with supplied mounting material
• one NEMA 17 stepper motor
• one CNC motor angle for NEMA 17
• two *T*-nuts slot 10
• two screws M8 × 16
• one Bowden-tube 500 mm

**Fig. 33:**
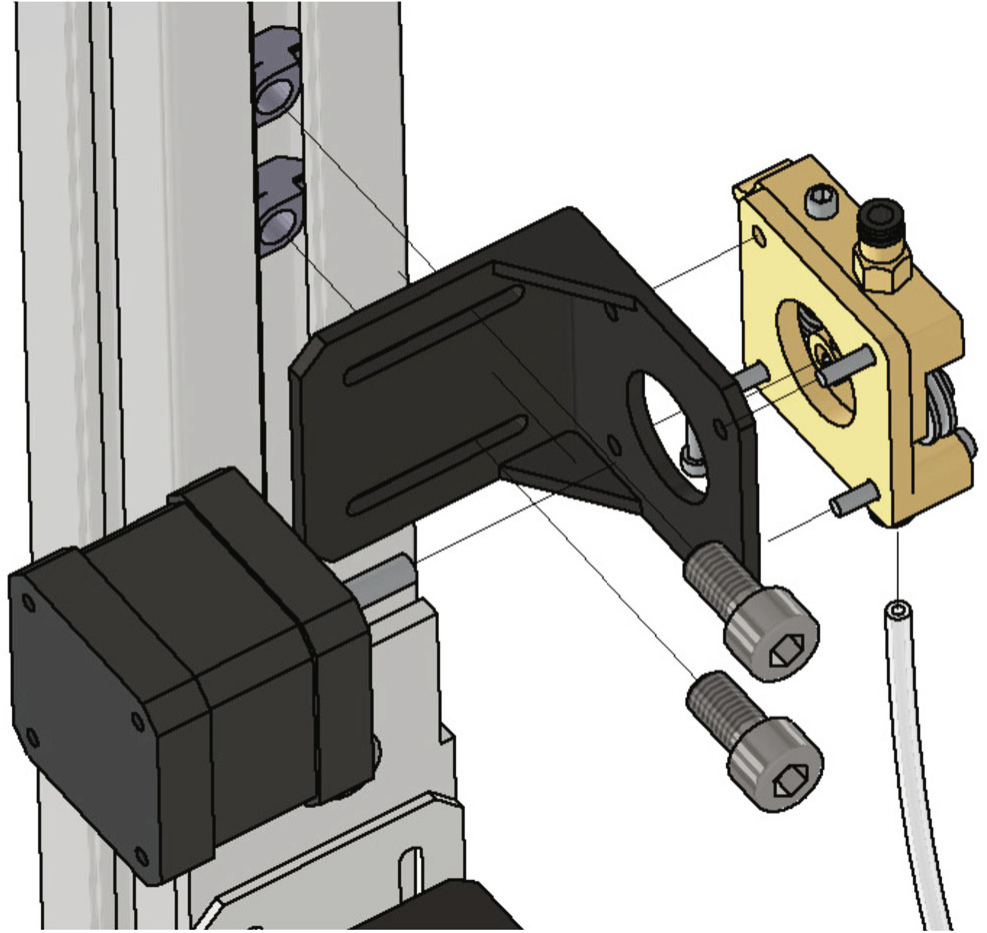
Mounting of the extruder to the frame.

The extruder gets attached to the frame with a motor angle, two *T*-nuts and two screws M8 × 16 and the Bowden-tube is inserted into the quick coupling of the extruder and the extrusion head respectively as shown in Fig. 33.

The MEX-system is based on a standard gantry-system used in common 3D-printers like Prusa MK3. There are many reasons for choosing this setup: On the one hand is the design very simple and easy to build. Other systems like CoreXY-printers [16], [17] or H-bots need usually long belts which must be redirected several times. In a CoreXY-setup, the belts also run on several levels, resulting in a complicated setup. The advantages of such a setup are higher printing speed. However, this only plays a subordinate role in the present measurement setup, since the primary aim is to print test specimens with small dimensions. The time savings due to higher printing speeds are only marginal here. In all common CoreXY-systems, the build platform also moves up or down along the z-axis. During the peeling tests, a tensile force is applied by the measuring unit in exactly the same direction. The mechanics would therefore have to be designed for large loads. Otherwise, in the case of very high adhesion, the print bed could be displaced instead of the specimen being detached. So, it was decided to move the x-axis up and down.

A Cartesian design, on the other hand, allows each axis to be constructed and controlled almost independently of the others. The use of steep thread spindles can further reduce the complexity of the design. Here, components such as idler pulleys or belt tensioners are eliminated. In addition, steep threaded spindles offer better positioning accuracy than toothed belts and are maintenance free. With a Cartesian setup, the print bed can simply move in the y-direction instead of the z-direction. As a result, the load direction during the peel tests is perpendicular to the direction of movement. In principle, this means that no displacement of the print bed is possible due to tensile forces applied by the measuring unit. Furthermore, adaptation to higher forces can be carried out very easily, since only larger guide shafts would have to be installed.

Another advantage of this system is the possibility to install other hardware very easy. The extrusion head can be replaced by any other extrusion head with a groove mount which is a widespread standard. For this, only the screws holding the extrusion head connectors together must be loosened. Also, the extruder can be replaced by loosen two screws and the bowden tube.

The change of the build surface is also designed to be very quick. It is clamped with a screw in each corner. By using a BL-touch, a height map of each surface can be created to ensure that the distance between nozzle and print bed is always constant.

### Electronic

**Fig. 34:**
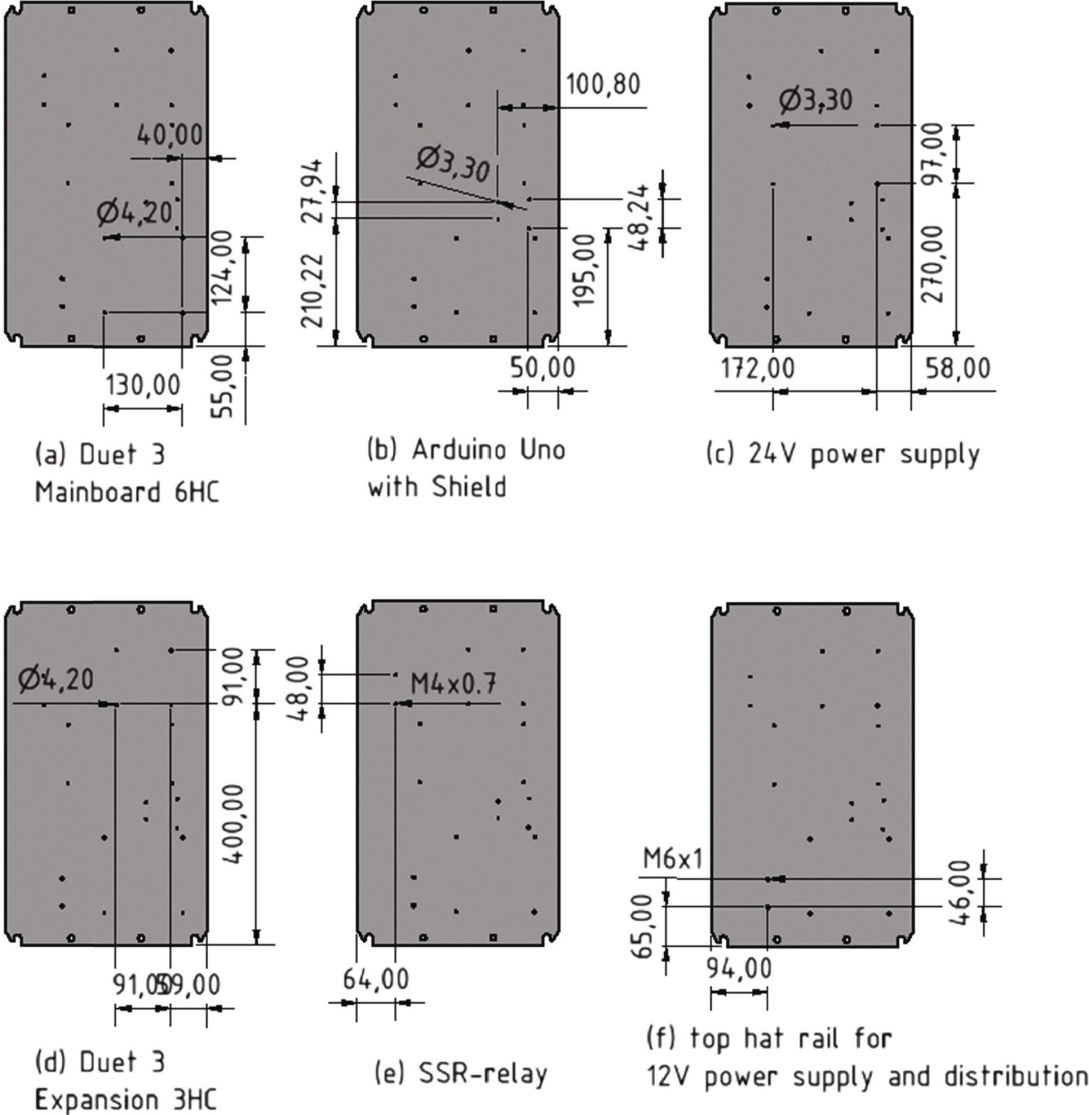
Position and diameter of the holes and threads in the electronics base plate needed for the different boards and power supplies.

The installation of the electronics requires the handling of 230 V lines, which is why only sufficiently qualified persons should undertake the wiring. Several boards and power supply units must be mounted into a control cabinet. In this work a control cabinet type ”Rittal AX 1038.000″ [18] was used. A base plate is included in the scope of delivery of this control cabinet. Following this base plate is called electronics base plate. This allows the mounting of the electronics outside the control cabinet. The first step is to drill the holes and threads, as shown in Fig. 34, for attaching the electronics.

Next the boards will be screwed to the electronics base plate. For this step following parts are required:

• Duet 3 Mainboard 6HC
• Duet 3 Expansion 3HC
• eight spacer bolts M4
• eight screws M4 × 8
• eight nuts M4

**Fig. 35:**
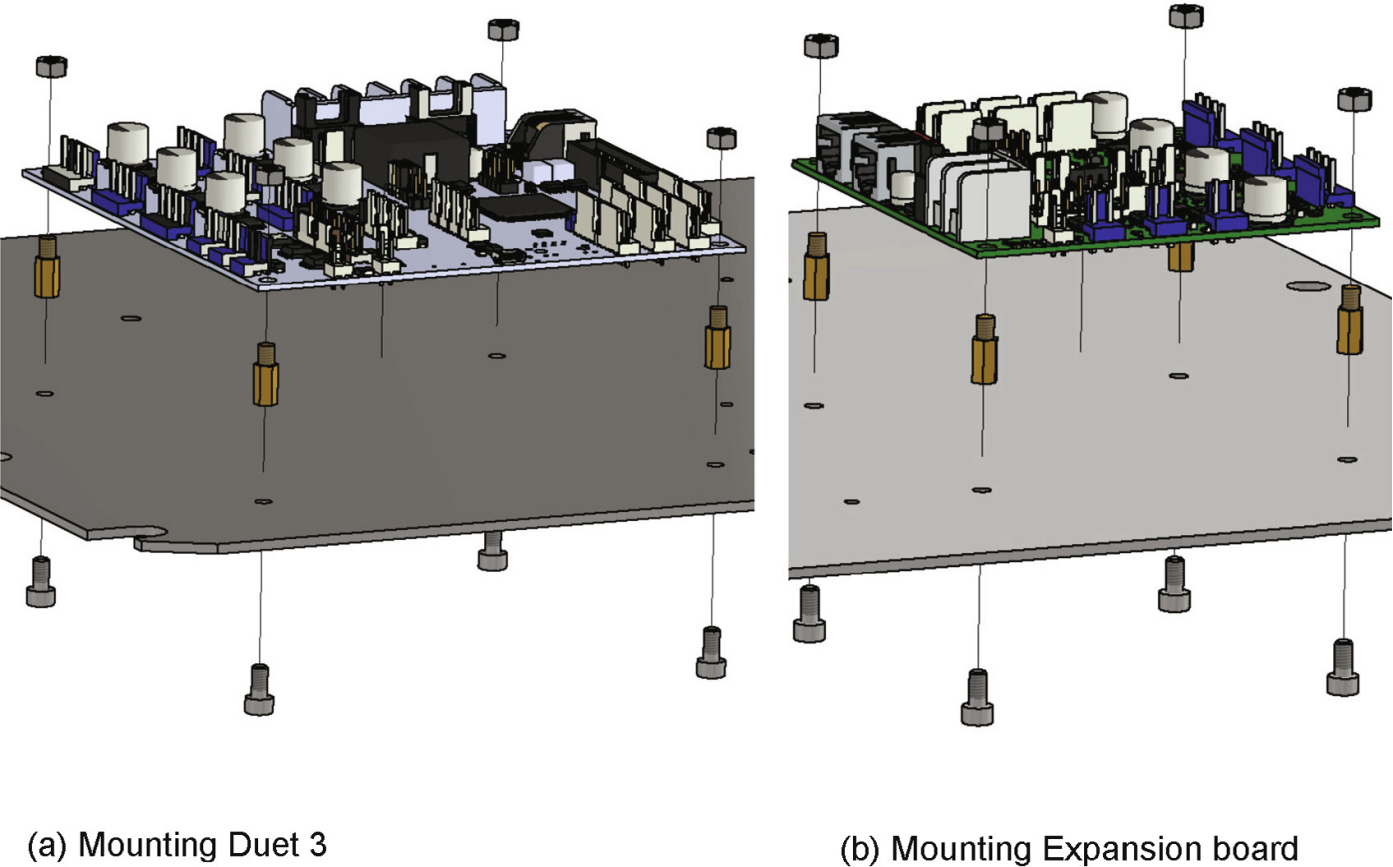
Mounting of the controller boards to the electronics base plate.

The spacer bolts are attached to the base plate with the M4 × 8 screws while the boards are set on top of the spacer bolts and secured with four nuts M4 each (see Fig. 35). Which holes are used can be seen in Fig. 34.

After the Duet 3 boards the Arduino Uno and the load cell shield are getting mounted to the electronics base plate. For this the below listed items are needed:

• Arduino Uno
• load cell shield
• four spacer bolts M3
• four screws M3 × 8
• three nuts M3

**Fig. 36:**
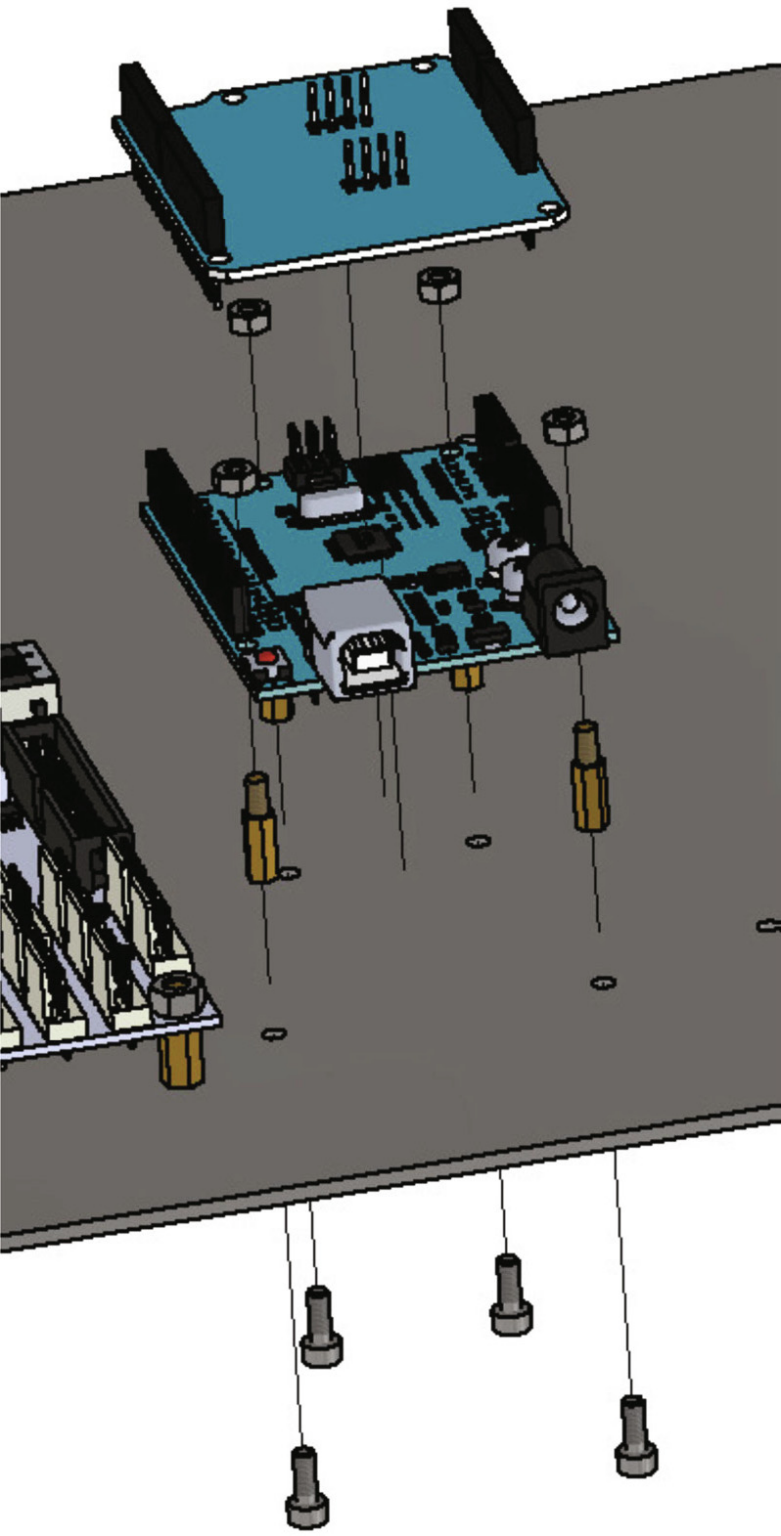
Mounting the Arduino Uno with load cell shield to the electronics base plate.

The Arduino Uno is mounted the same way as the Duet 3 boards in the step before (see Fig. 36, Fig. 34). After attaching the Arduino Uno to the electronics base plate, the load cell shield is plugged in.

After the Arduino Uno the SSR-relay will be attached to the electronics base plate with following parts:

• one DELIXI Solid State Relay CDG1-1DA / 480V AC 40 A SSR
• two screws M4 × 8

**Fig. 37:**
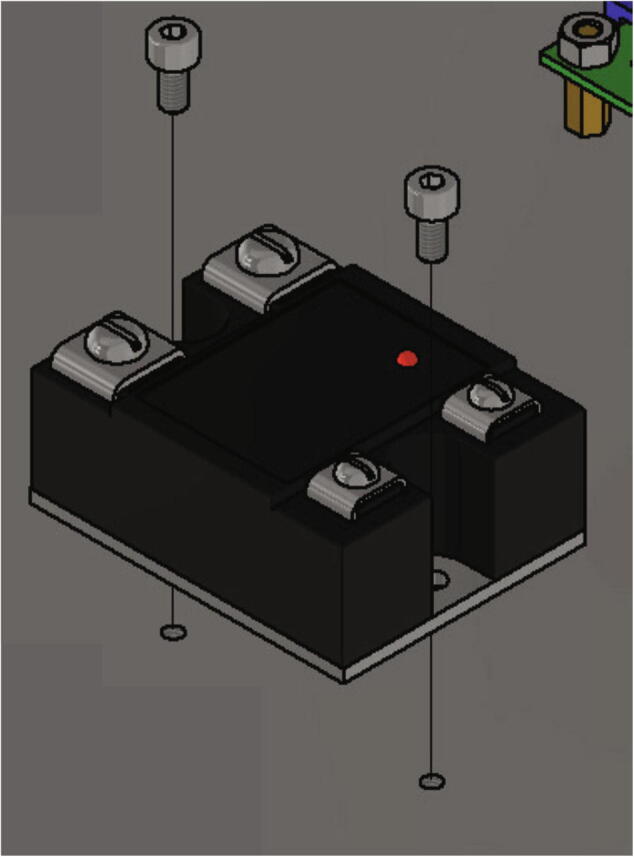
Mounting of the SSR-relay.

The SSR-relay is screwed to the electronics base plate with two screws M4 × 8 as shown in Fig. 37, Fig. 34.

Next the top hat rail will be mounted to the electronics base plate:

• one top hat rail with length 120 mm
• two screws M6 × 8

**Fig. 38:**
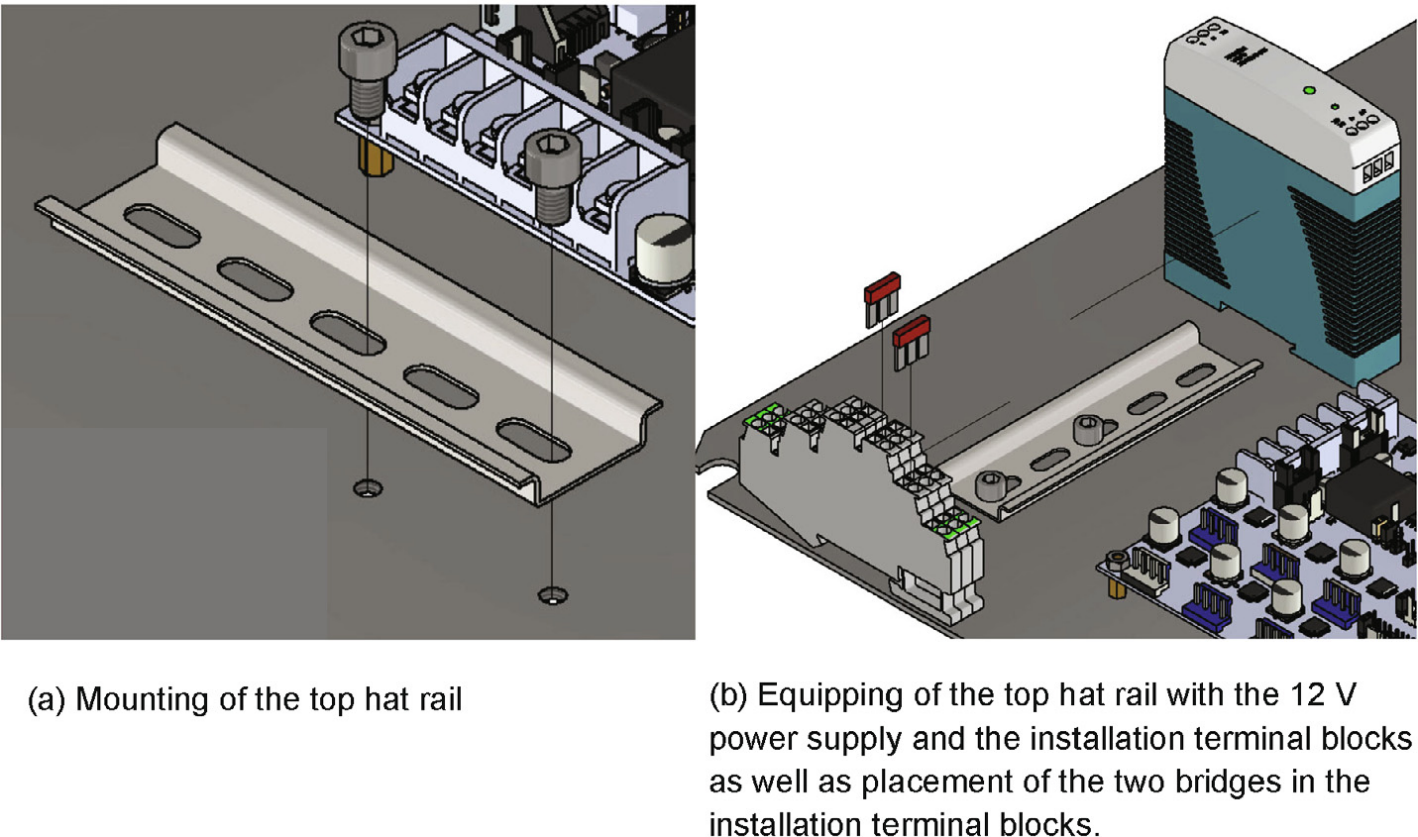
Mounting of the 12 V power supply unit with the corresponding top hat rail to the electronics base plate.

The top hat rail is screwed to the electronics base plate with two screws M6 × 8 as shown in Fig. 38a) and 34.

Now the top hat rail is equipped with following parts:

• one power supply 12 V
• three installation terminal blocks
• two bridges

The power supply 12 V and the three installation terminal blocks are pushed onto the top hat rail and the bridges are set into the installation terminal blocks as shown in Fig. 38b). Following the three installation terminal blocks with the mounted bridges are called distributor. It is used to distribute 230 V voltage to the power supply units and the heated build platform.

The 24 V power supply unit is mounted next with following parts:

• one power supply 24 V type “Wantai Netzteil 24 V 14.6 A 350 W”
• four screws M3 × 8

**Fig. 39:**
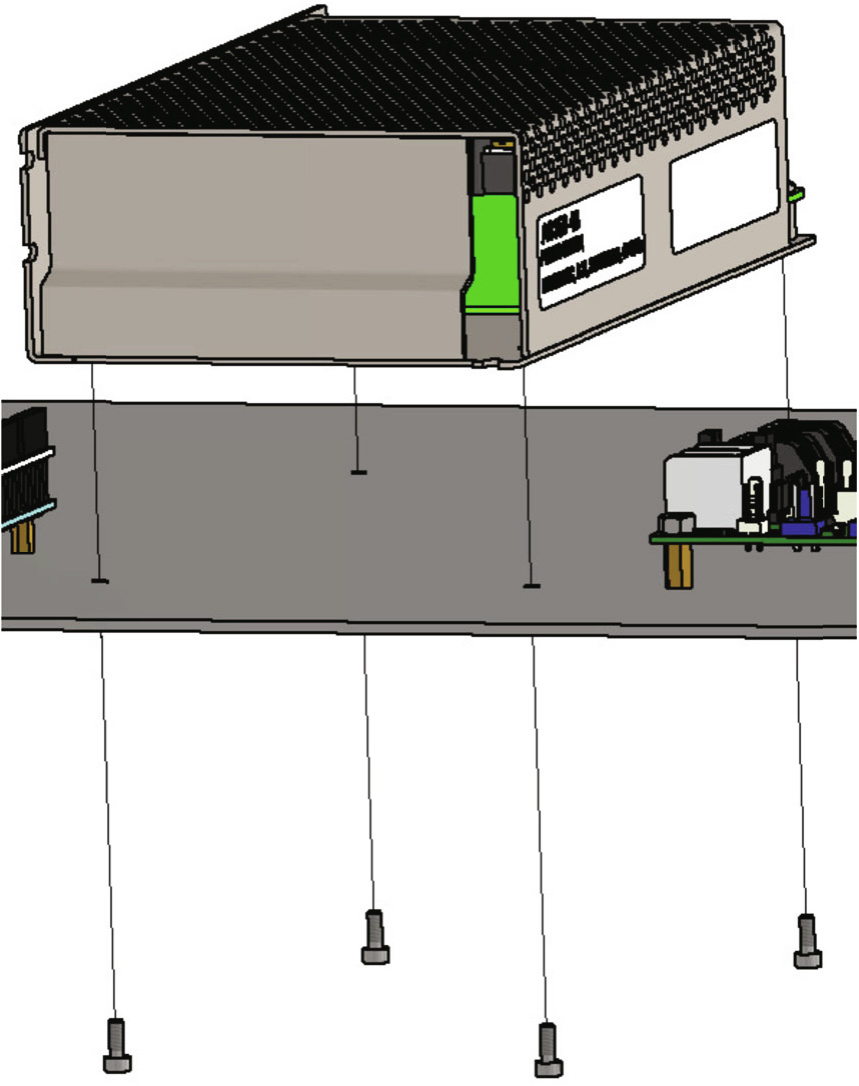
Mounting of the 24 V power supply unit.

The 24 V power supply is screwed to the electronics base plate with four screws M3 × 8 (see Fig. 39, Fig. 34).

**Fig. 40:**
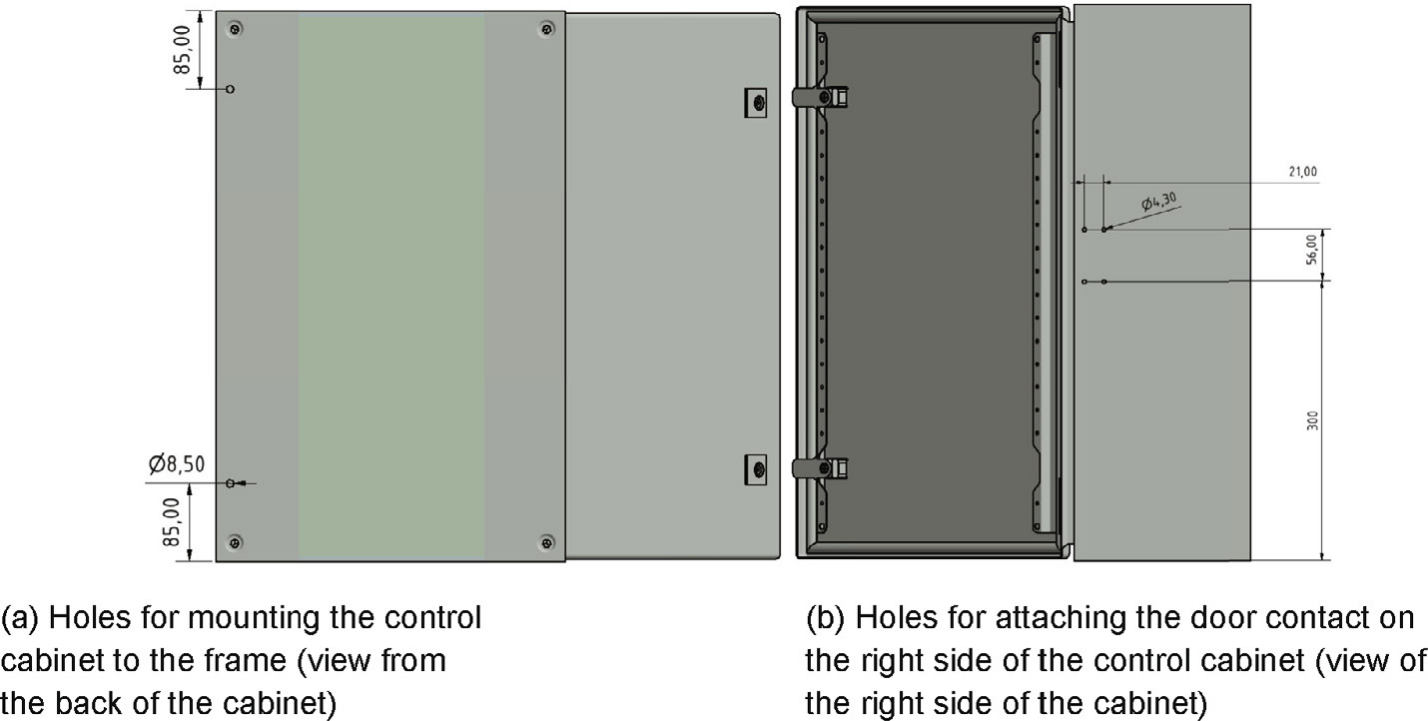
Placement and diameter of the drilled holes in the control cabinet.

**Fig. 41:**
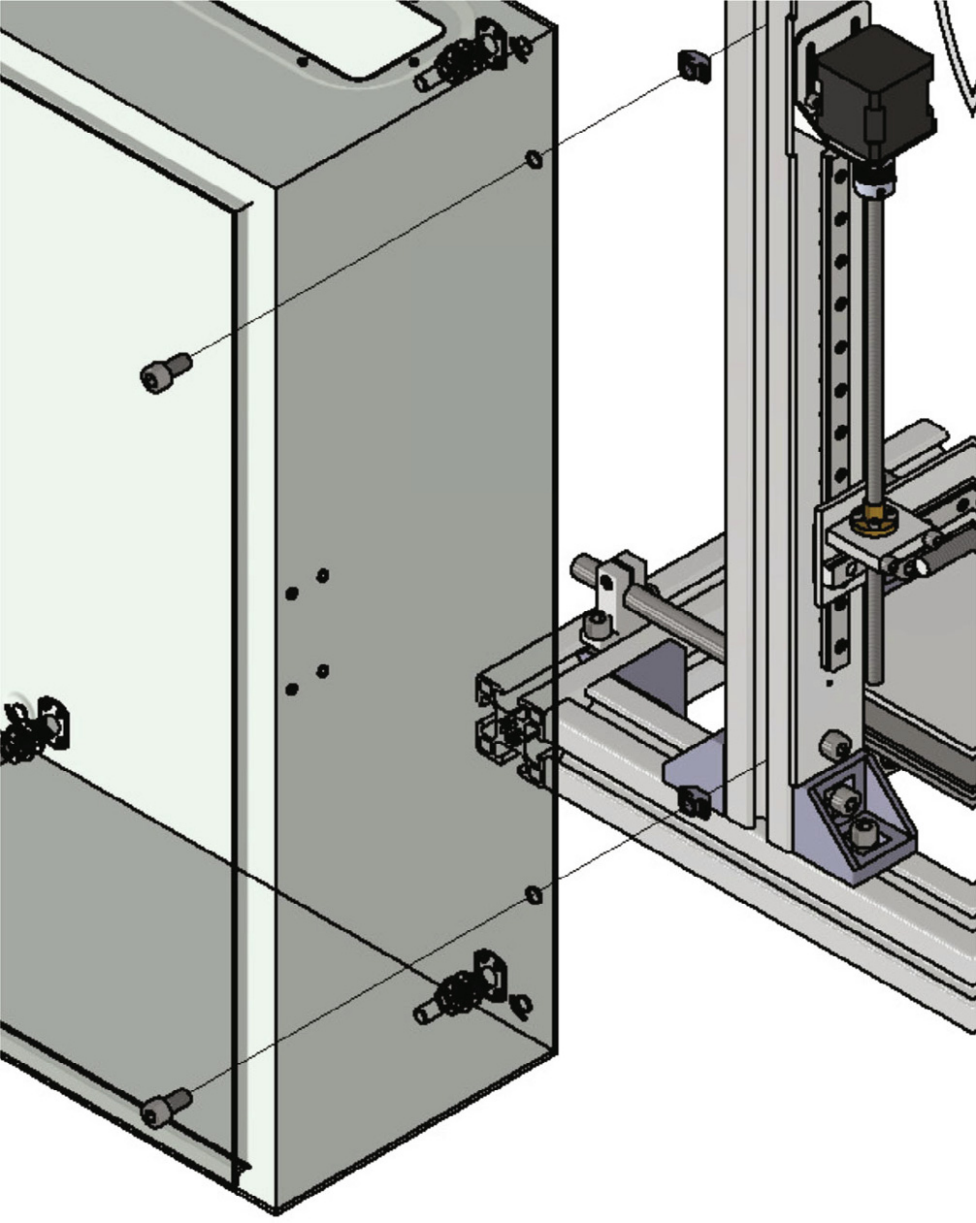
Mounting of the control cabinet to the left side of the frame.

The next step is drilling holes into the control cabinet which will be used for mounting it to the frame and for attaching the door contact. Diameter and placement of the holes are shown in Fig. 40 and the correct orientation of the control cabinet is shown in Fig. 41.

The next step is mounting the control cabinet (electronics base plate must be removed) to the left side of the frame with following parts:

• one control cabinet
• two screws M8 × 16
• two *T*-nut slot 10

The screws M8 × 16 are put through the holes drilled during in the step before and screwed into the *T*-nuts as shown in Fig. 41.

**Fig. 42:**
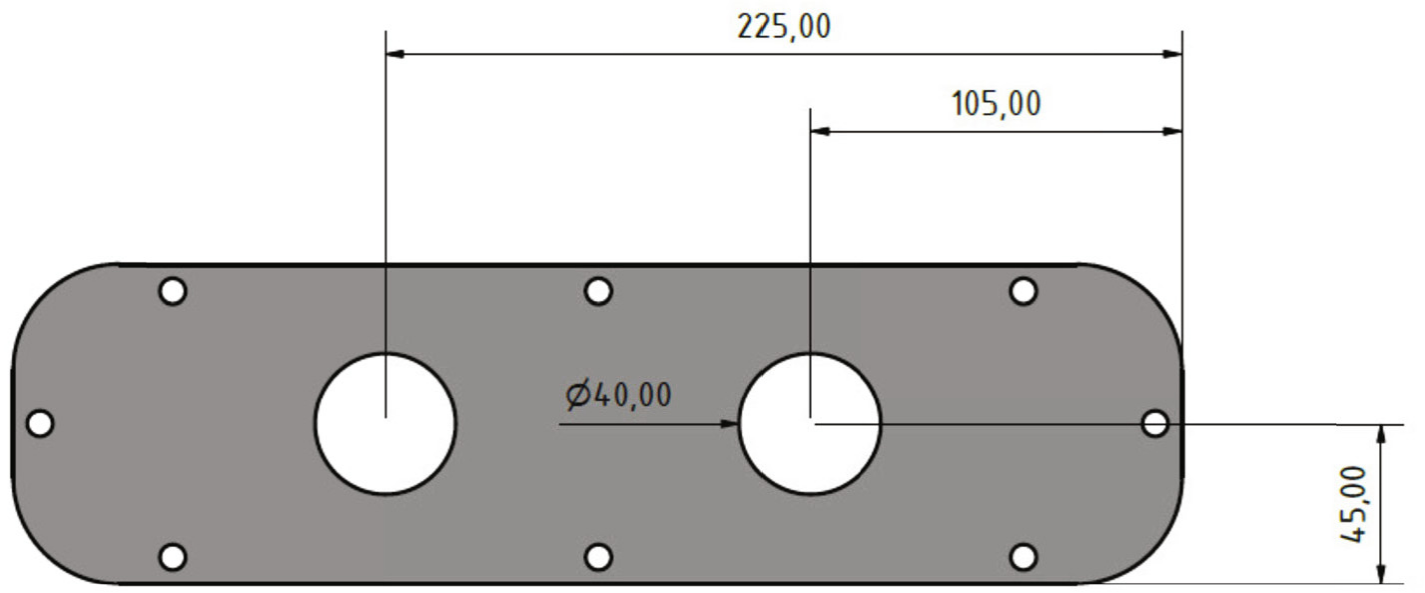
Position and diameter of the holes in the top plate.

Now two holes must be drilled into the top plate of the cabinet as shown in Fig. 42.

**Fig. 43:**
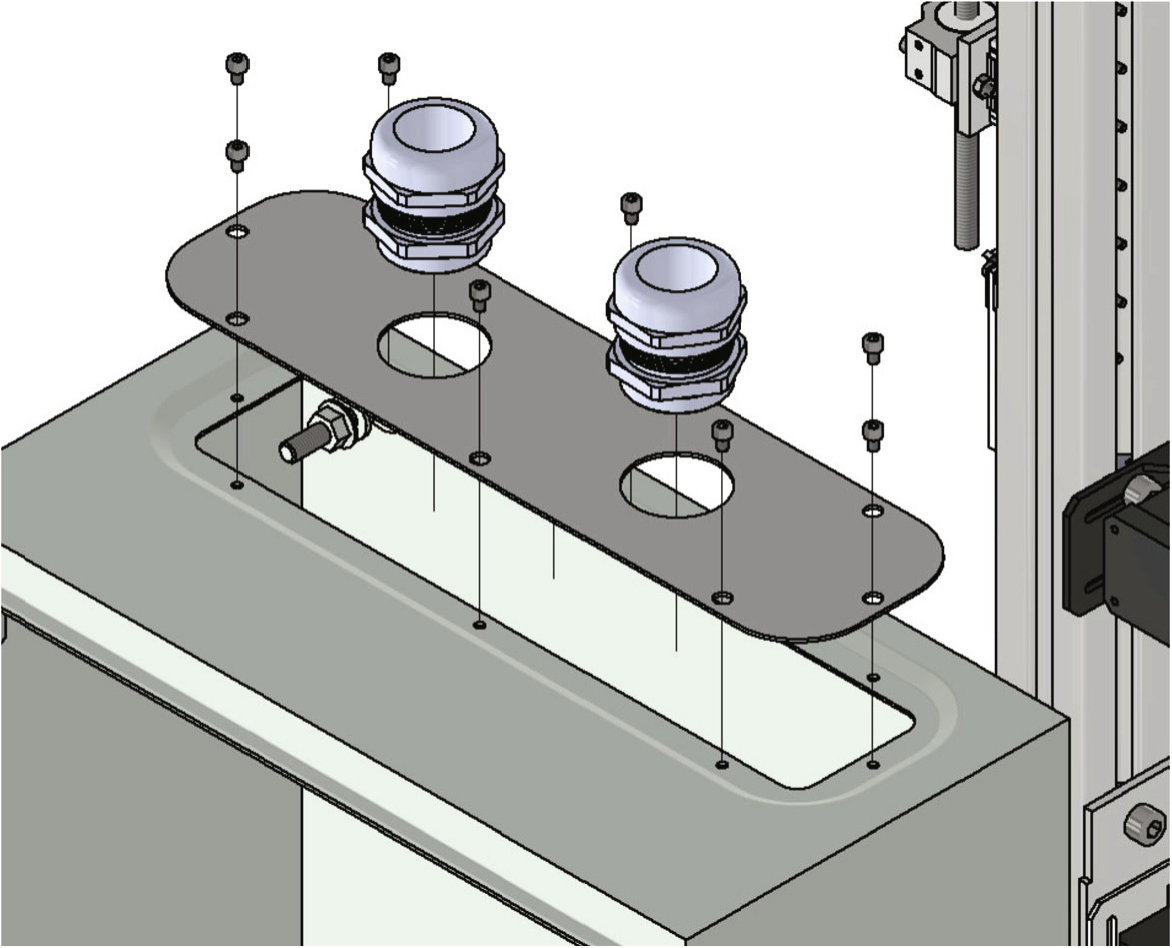
Mounting the top plate with two cable glands.

The top plate is attached to the top side of the control cabinet and two cable glands are put through the holes with 40 mm diameter (see Fig. 43). In this step the screws included in the scope of delivery of the control cabinet are used.

**Fig. 44:**
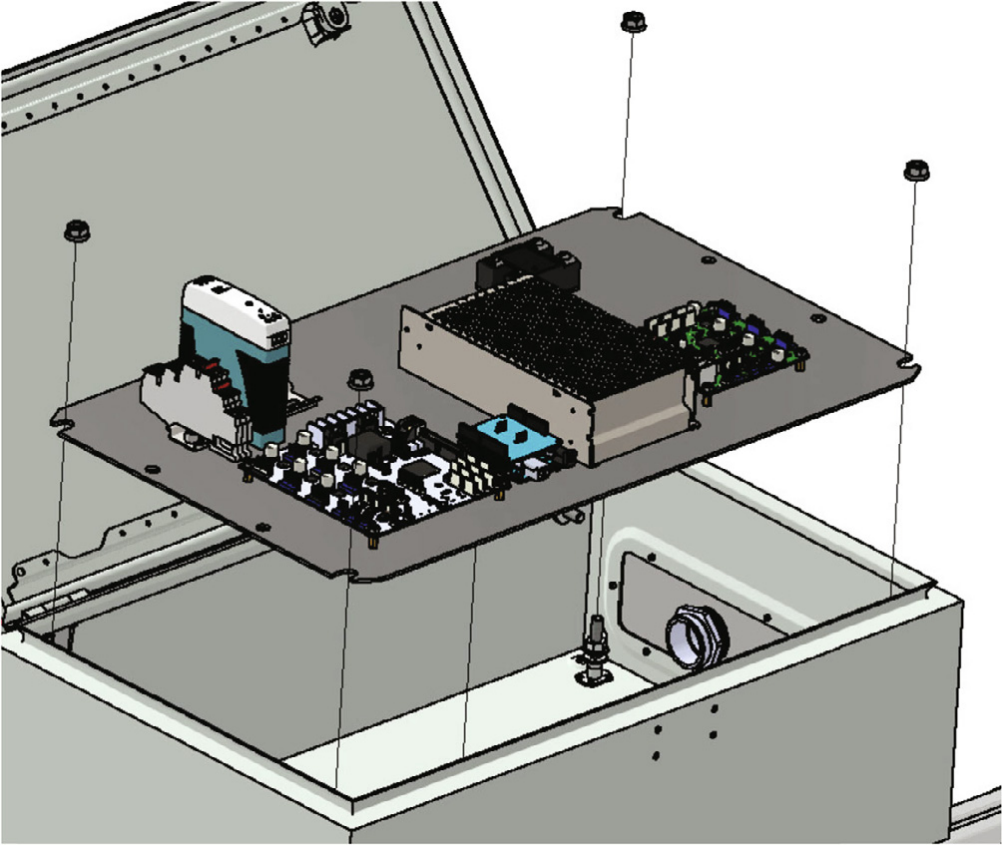
Mounting the electronics base plate.

Now the plate with the electronics can be placed into the control cabinet as shown in Fig. 44. The mounting material included in the scope of delivery of the control cabinet is used.

**Fig. 45:**
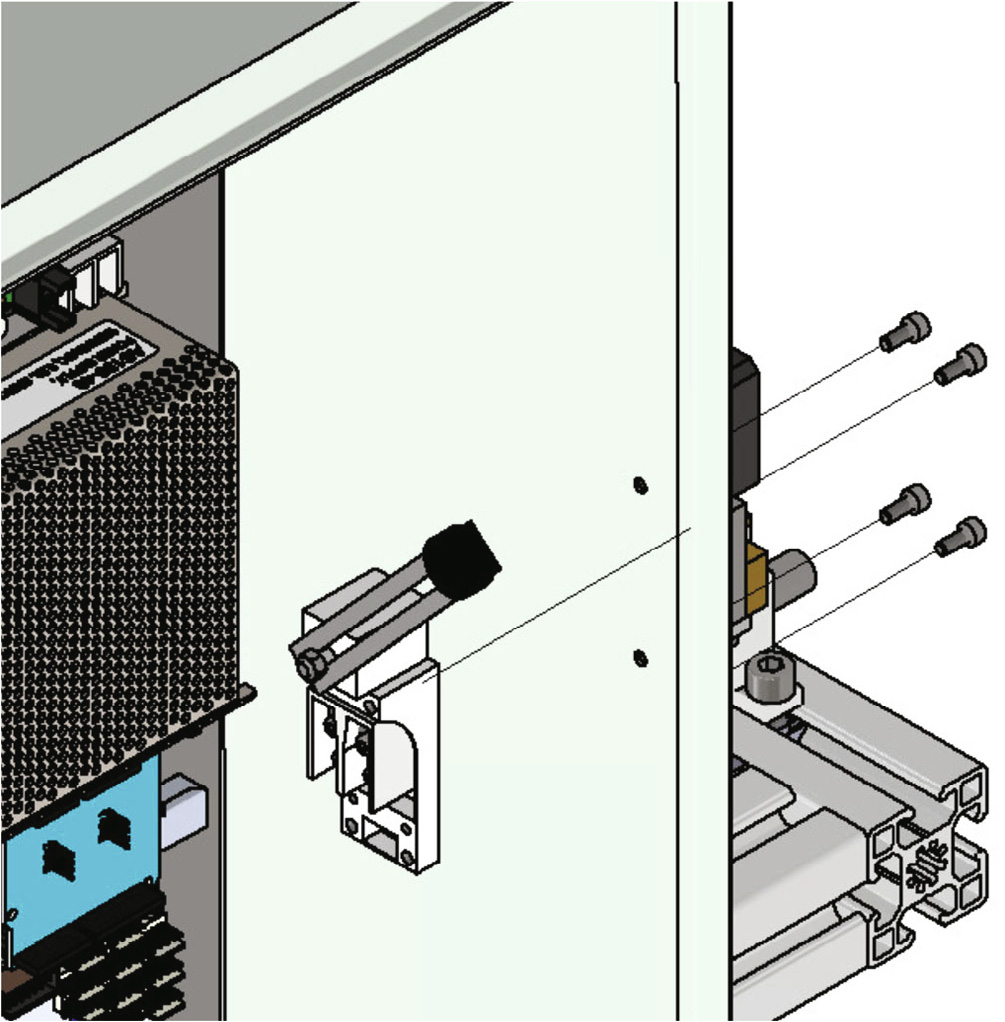
Mounting of the door switch.

After placing the electronics inside the control cabinet, the door switch can be attached with four screws M4 × 10 as shown in Fig. 45:

The next step is to wire the boards together. A circuit diagram of all wires can be found below. For this the following parts are necessary:

• Wire with 2.5 mm^2^ cross section in following colors: blue, black, yellow/green striped, red, white, brown, green
• one CAN-Bus cable
• two WAGO 221-415 connection clamps
• crimp connectors from the scope of delivery of the Duet boards

In Table 3 the assignment of the pins of the Duet 6HC mainboard and of the Duet 3 3HC expansion board are listed. The entire documentation with the pinout of the boards can be looked up under [19], [20]. For instructions on how to install or configure firmware, please also refer to [19]. The 12 V power supply unit has only one output for ground and one for + 12 V but several pins must be wired to them. A solution is the use of connection terminals like WAGO 221-415.

**Table 3:** Wiring of the Duet 3 6HC board and the Duet 3 3HC expansion board.

**Duet 6HC Mainboard**	
Pin Connected to	Pin Connected to
POWER IN GND	V- 12 V power supply unit
POWER IN VIN	V + 12 V power supply unit
OUT 0 POWER IN GND	V- 12 V power supply unit
OUT 0 POWER IN V OUT 0	V + 12 V power supply unit
OUT 0 V OUT0	extrusion head heating cartridge (polarity does not matter)
OUT0-	extrusion head heating cartridge (polarity does not matter)
OUT2 out2	- SSR-relay
OUT2 V FUSED	+ SSR-relay
TEMP 0 VSSA	thermistor cartridge extrusion head (polarity does not matter)
TEMP 0 temp0	thermistor cartridge extrusion head (polarity does not matter)
TEMP1 VSSA	thermistor heated build platform (polarity does not matter)
TEMP1 temp1	thermistor heated build platform (polarity does not matter)
IO 6 3.3 V	V mechanical endstop
IO 6 io6.in	S mechanical endstop
IO 6 GND	G mechanical endstop
IO 7 io7.in	BL-Touch D2 (white wire)
IO 7 GND	BL-Touch GND (black wire)
IO 7 io7.out	BL-Touch D11 (orange wire)
IO 7 5 V EXT	BL-Touch + 5 V (red wire)
IO 5 GND	BL-Touch GND (brown wire)
Driver 0 all 4 pins	MEX-System x-axis motor
Driver 1 all 4 pins	MEX-System y-axis motor
Driver 2 all 4 pins	MEX-System first z-axis motor
Driver 3 all 4 pins	MEX-System second z-axis motor
Driver 4 all 4 pins	MEX-System extruder motor
CAN -FD × 2	Duet 3 expansion board 3HC CAN-FD in
**Duet 3HC Expansion board**	
CAN -FD × 2	Duet 3 mainboard board 3HC CAN-FD in
POWER IN GND	V- 24 power supply unit
POWER IN VIN	V + 24 power supply unit
DRIVER 0 all 4 pins	first NEMA 23 motor from measuring unit
DRIVER 1 all 4 pins	second NEMA 23 motor from measuring unit

**Table 4:** Explanation of all options in the config.json-file of the measurement software.

**key-word**	**example**	**hints**
”COM”:	”COM3″	Defines the COM-Port for the communication with the Arduino Uno. It must be written in quotation marks.
”baud rate”:	115,200	The baud rate for the communication with the Arduino Uno. This must be 115,200 without quotation marks.
”location”:	”C:/measurement data”	The path where the .csv file with the measured data is saved. It must be written in quotation marks.
”IP address”:	”192.168.178.94″	The IP-address of the Duet 3 board. It must be written in quotation marks.
”password”:	”WYdfsVdbh32″	The password of the Duet 3. It must be written in quotation marks.
”y axis section”:	314.185	The y-axis section of the line equation from the calibration of the load cell. The value must be written without quotation marks.
”slope”:	−0.951	The slope of the line equation from the calibration of the load cell. The value must be written without quotation marks.

**Fig. 46:**
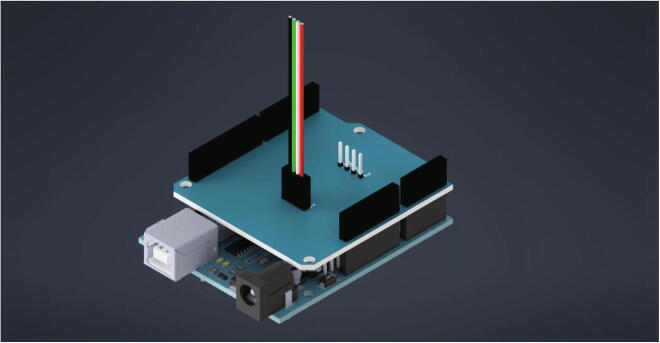
Correct wiring of the load cell.

The load cell is wired to the strain1-input of the load cell shield as shown in Fig. 46. The order of the colors is important.

Hereafter will be explained how the 230 V lines are wired. This must be done by appropriately qualified personnel only. The three cores of the 230 V cable are wired to the distributor on the top hat rail. The outer conductor is routed via the door contact switch in this case. All power supply units must be wired with the distributor. The heated build platform is also connected to the distributor but one cable is routed through the SSR- relay. Also, the grounding of the control cabinet is wired to the distributor as shown in the wiring diagram below.

**Figure:**
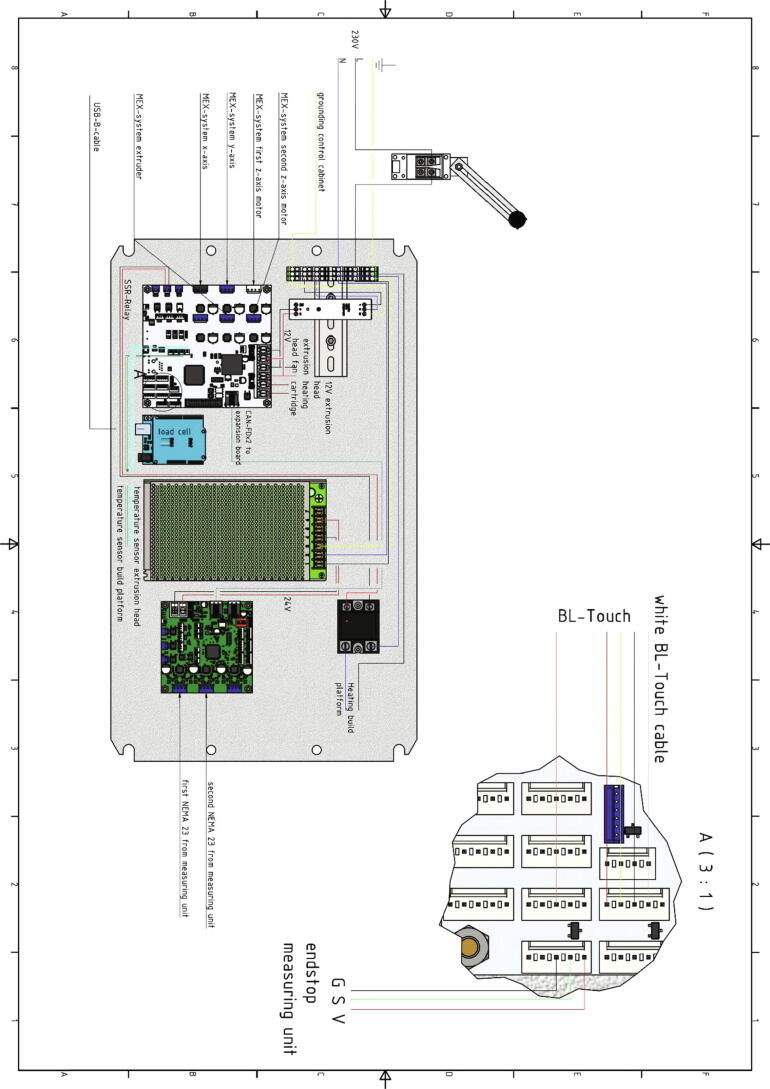

The next step is the commissioning of the Duet 3 as explained under [19]. All files can be found in Table 1. Before uploading the config.g file to the Duet the network configuration must be done. It is highly recommended to use a static IP-address, netmask and gateway. The python script for taking measurements needs this for communication with the test device. Due to a static IP-address the python-script must be adapted only once. Further, HTTP, FTP and Telnet should be activated. This means that the testing device can be controlled via internet. Therefore, a password should be set.

**Fig. 47:**
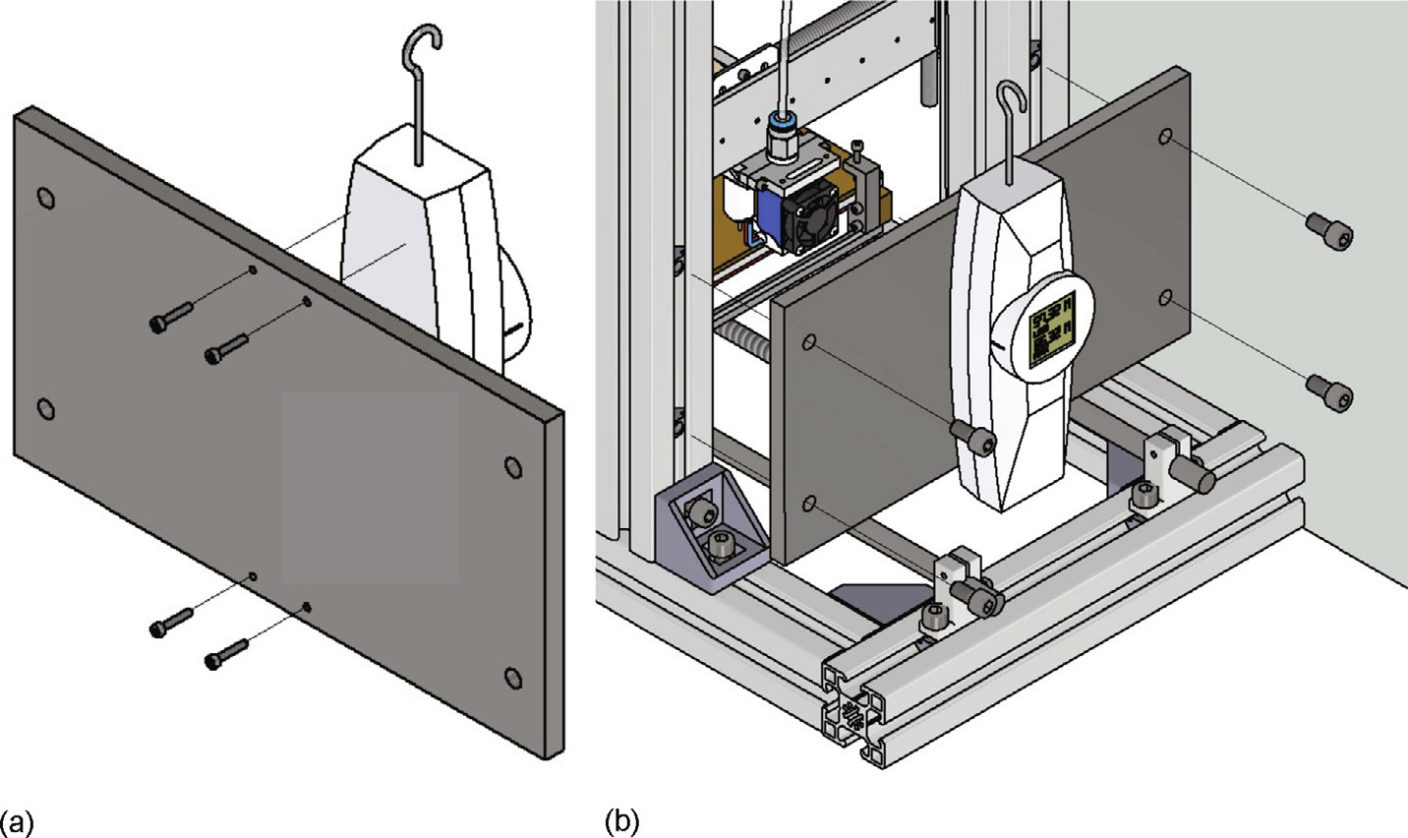
Mounting a commercial force measurement device to the frame.

After the Duet 3 Board the Arduino Uno is commissioned. A manual can be found under [21] and the necessary .ino-file is attached to the design file repository (see Table 1). This .ino-file must be uploaded to the Arduino Uno. Next, the z-probe must be calibrated as explained under [22], [23]. The load cell must be calibrated before it can be used. This is done by loading a known force to the load cell and reading the sensor values. For this a commercial force sensor like the beslandstool NK-500 was attached to a steel plate (see Fig. 47a)) which was screwed to the frame as shown in Fig. 47b). After finishing the calibration, the commercial force sensor with its mount must be removed from the frame.

**Fig. 48:**
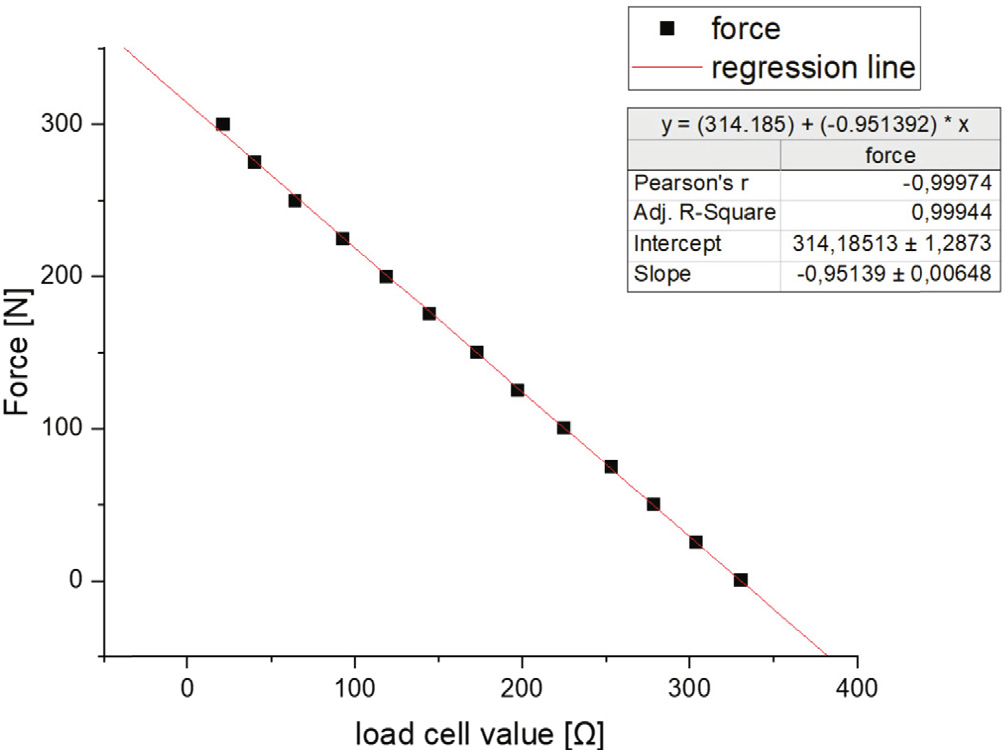
Measurement series with a regression line for calibrating the load cell. On the x-axis the measured values of the load cell and on the y-axis the force measured by the commercial force sensor are plotted.

To calibrate the load cell with this setup the hook must be hooked in the eyelet of the commercial sensor. If the measurement axis moves upwards a tensile force is loaded both to the load cell and the commercial sensor. Now the sensor output of the load cell can be compared to the force measured by the commercial force measurement system. The values measured by the load cell can be read by the serial monitor of the Arduino IDE. For the whole measurement range of the load cell forces should be applied and the corresponding load cell values noticed. With these pairs of values, a diagram as shown in Fig. 48 can be drawn. Through these points a regression line can be fitted and a linear equation set up. The measurement software in the appendix uses such a linear equation to convert the load cell values into the corresponding forces. Due to the fact that each load cell has a different characteristic curve, the calibration must be performed again after each change of the load cell.

**Fig. 49:**
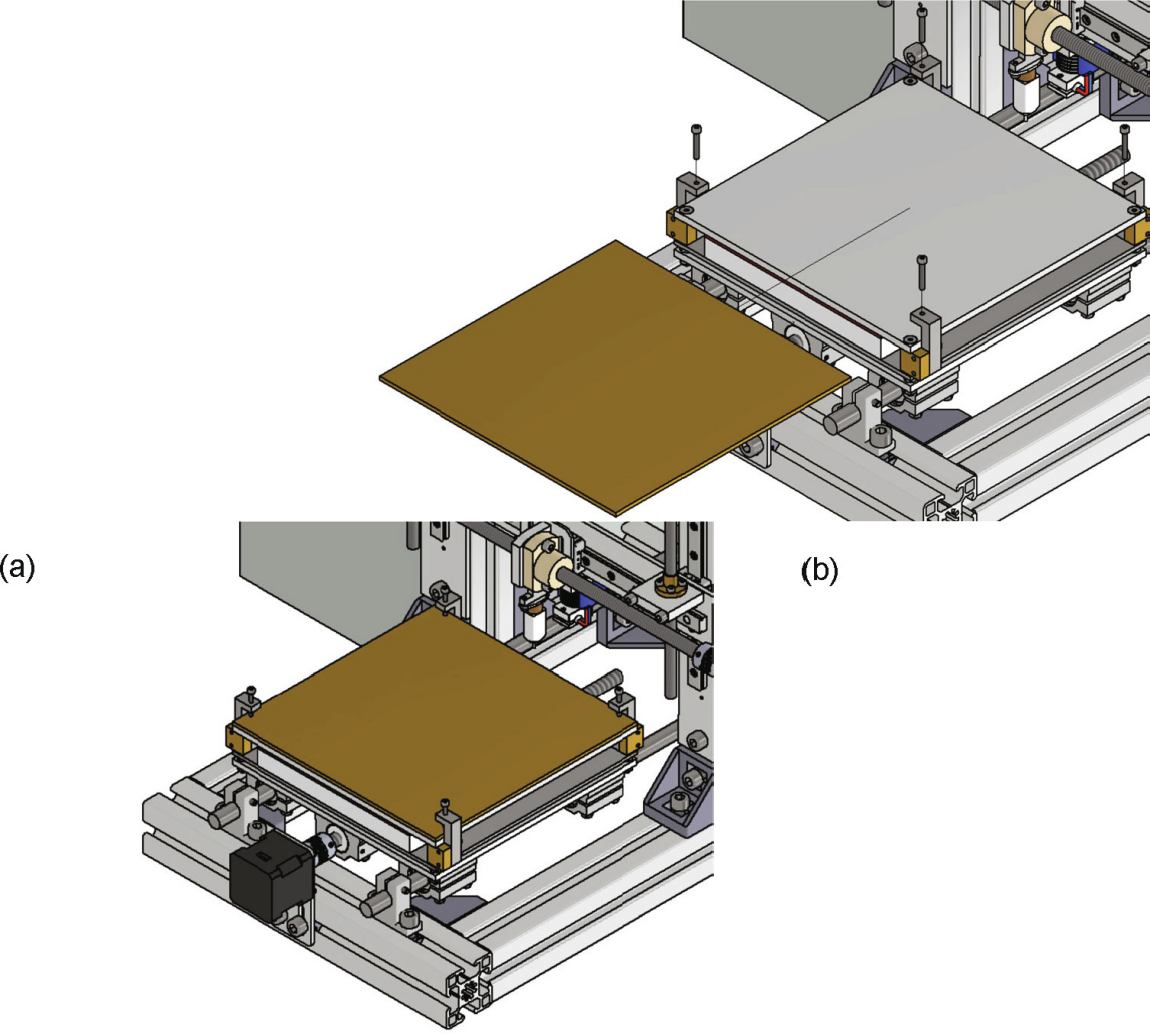
Insertion of a build surface.

After calibrating the load cell, the measurement software must be configured. This is done by editing the config.json file. The first column contains the key-words for the software and must not be changed. In the second one are the values which must be adapted. In Table 4 all options are explained.

The USB-Voltage which supplies the Arduino Uno and the load cell was measured. The voltage varies between 5.06 V and 4.8 V, dependent if the multimeter was directly connected to the notebook or via a USB-Hub. Due to this the value which the load cell measures without any force moves in an interval between 333 and 338. This results in an offset up to −2,6N. To correct this the measurement program measures the offset before each measurement cycle. For this, twenty values are read from the load cell, the average value is determined and the corresponding force is calculated. This calculated force is subtracted as an offset from every force measured in the complete measurement cycle.

## Operation instructions

The first step of a measurement is the insertion of a build surface. It must have the dimensions 200 mm × 200 mm and a thickness of up to 5 mm. The build surface is put on top of the build platform and secured with four screws M3 × 20 screwed into the clamps in each corner as shown in Fig. 49a). The correct attached build surface is shown in Fig. 49b).

**Fig. 50:**
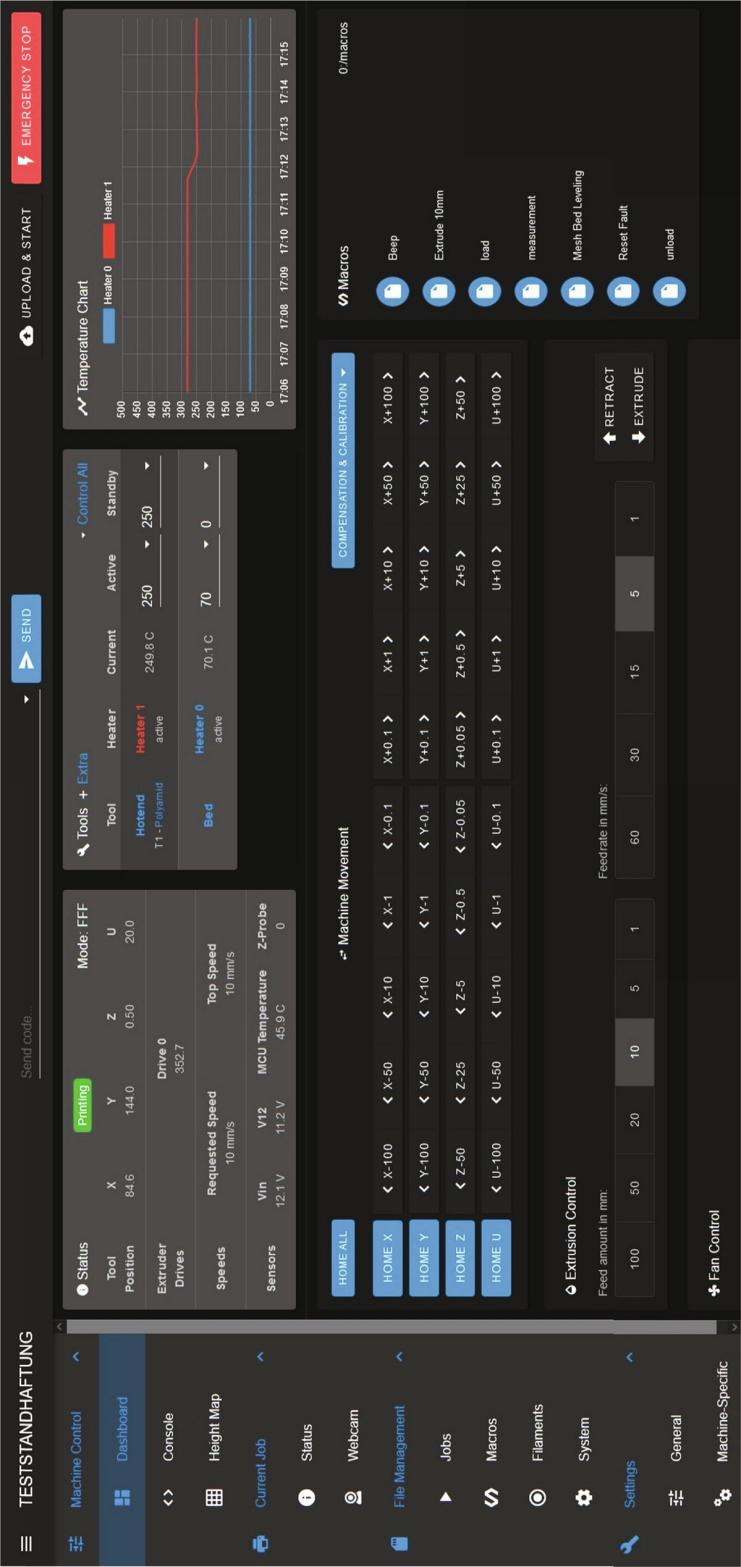
Dashboard in Duet Web control with different control elements and sensor values.

The measurement device is controlled via Duet Web Control (full documentation: [24] and github-repository: [25]), a website hosted from the Duet 3 board with a GUI. In Fig. 50 the dashboard can be seen. On the left side different menus are listed: Machine control to move all motors, to set processing temperatures or to send commands. Current Job gives an overview over the actual printed part like the already used filament or the remaining time. Also, a webcam can be implemented. Under File management the machine configuration can be seen or changed, macros can be defined or all uploaded printing files are listed and can be modified. In the menu ”Settings” are options to customize duet web control itself with color themes, preset temperatures or the language.

**Fig. 51:**
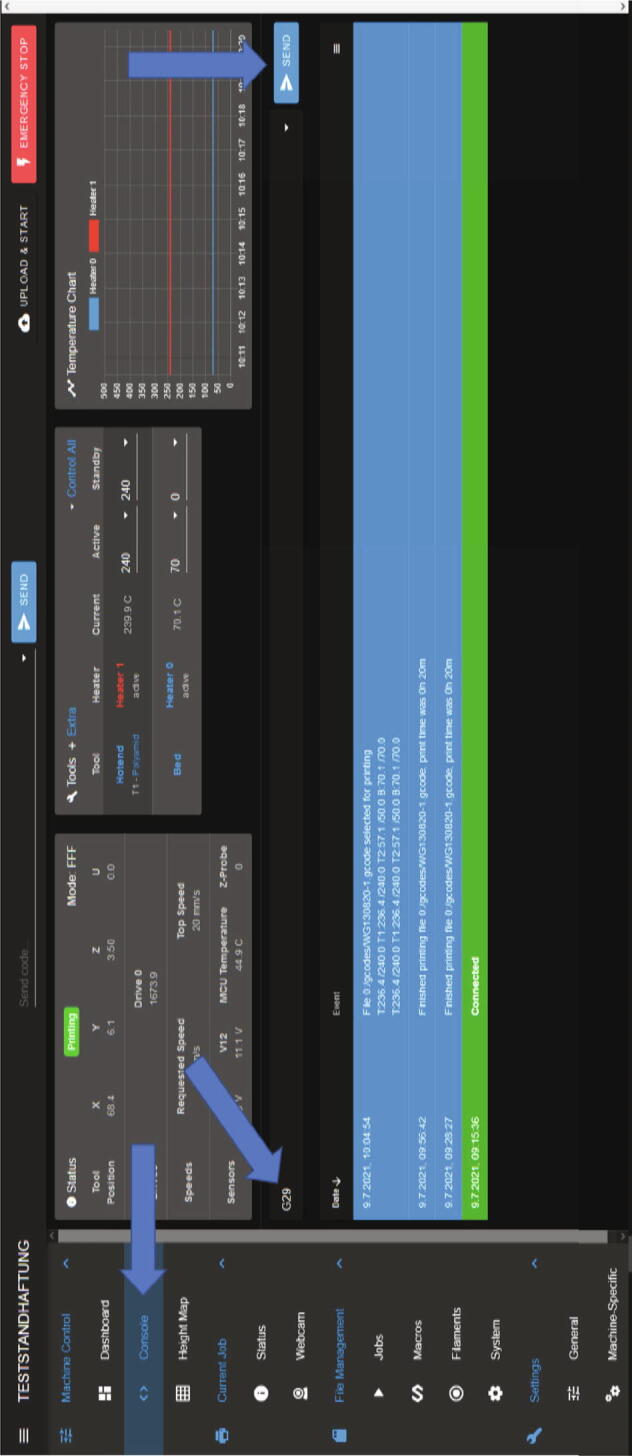
Sending commands via the console.

The next step is to create a height map of the build surface. For this a G29 command is sent via an entry for gcodes. Here the desired command is written and send to the board with the ”send”-button (see Fig. 51). The best results can be achieved if the MEX-system is already heated to the chosen process temperatures because a potential elongation due to the heat will be included in the measurement.

**Fig. 52:**
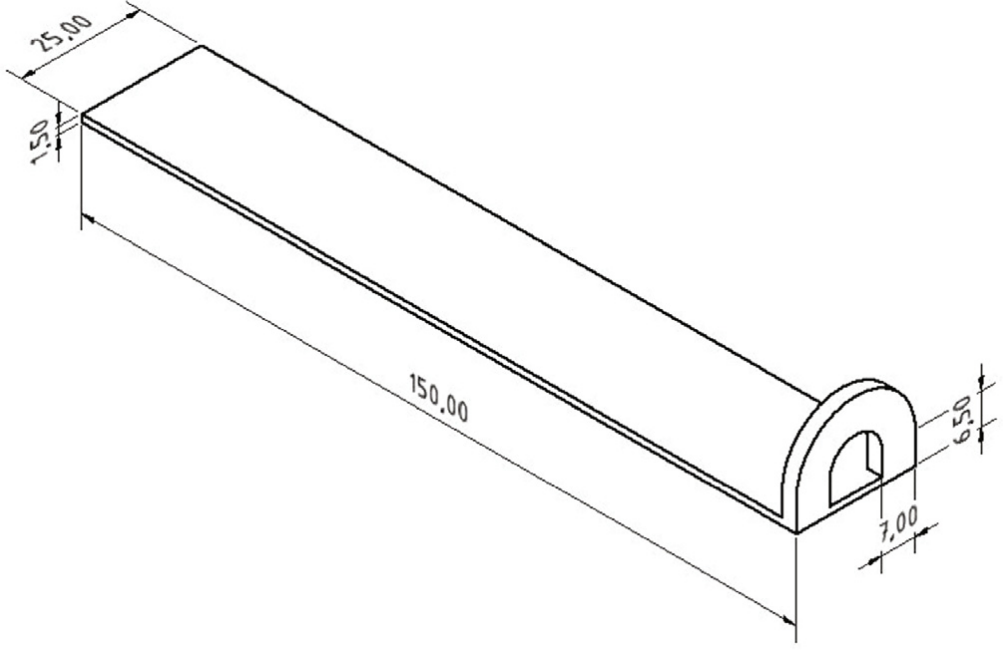
Specimen based on DIN EN 28510-1 with an eyelet added at a small side for applying peeling forces. The dimensions are given in millimeters.

**Fig. 53:**
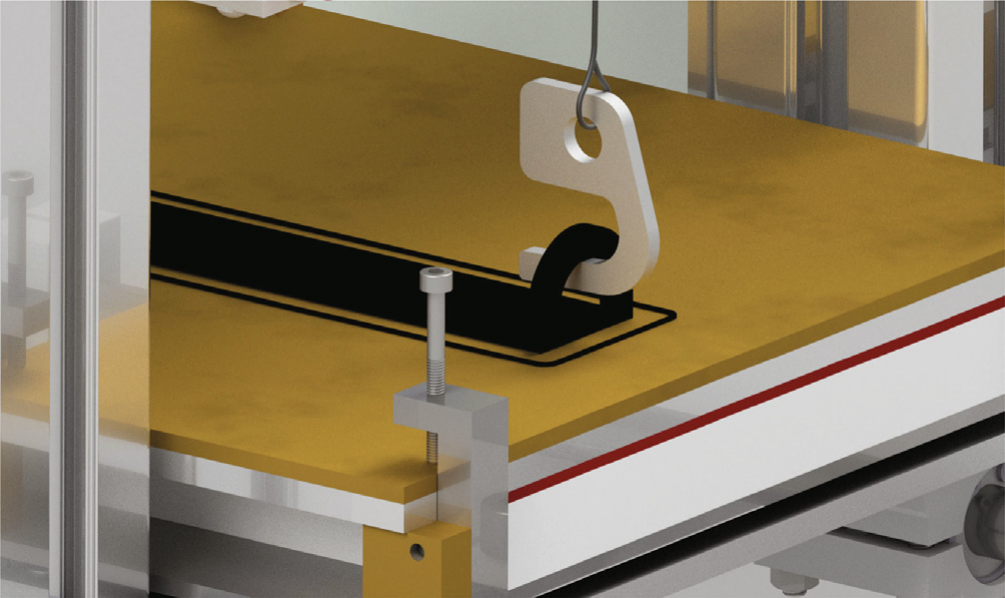
Insertion of the hook into the eyelet of the printed specimen.

After sending the G-29 command the MEX-system probes at defined points and generates a height map. The firmware fits a compensation plane through the measured points which is used to compensate tilt or uneven surfaces. The next step is printing the specimen. As described in section 1, its geometry is based on DIN EN 28510-1. An eyelet was added to take the hook of the measurement unit (see Fig. 53). The geometry of the specimen is shown in Fig. 52.

Starting a printing process can be done by starting the presliced gcode attached to the appendix (only for exact the same extrusion head, nozzle and extruder as listed above).

Another way is using the attached STL-file by slicing it via an external slicer and thus preparing the gcode.

After finishing the print, the measurement software (see Table 1) needs to be started. A name for the csv-file in which the measured forces will be saved must be entered and confirmed with the button. Next the ”start measurement” button must be pressed and the MEX-system moves to a parking position and the measurement unit starts moving its axis upwards. The hook of the measurement unit must be inserted into the eyelet of the specimen as shown in Fig. 53.

The result of a measurement cycle is a .csv-file as a list of the measured and recorded forces.

## Validation and characterization

In the following section the accuracy and precision of the single sensors and the measurement device in general, is discussed.

### Process temperatures

**Fig. 54:**
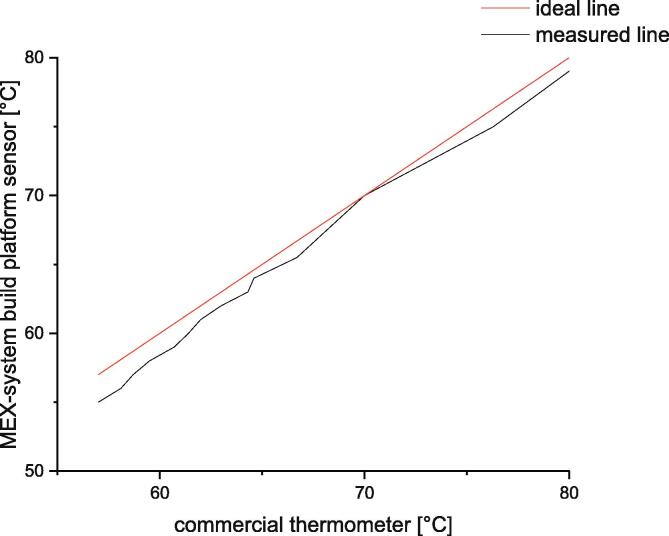
Comparison between the measured temperatures of the commercial thermometer Testo 735-1 and the sensor of the build platform. The red line shows an ideal case in which both sensor measure the same temperature. For each temperature was a single measurement taken. The accuracy of both sensors is ± 0.2 °C. (For interpretation of the references to color in this figure legend, the reader is referred to the web version of this article.)

The measurements of the temperature sensor which is mounted in the build platform are verified by comparing the measured temperatures to the values of a commercial temperature sensor. For this purpose, a thermometer type Testo 735-1 with a sensor type K 0602 0693 was used to measure the temperature on the build surface. These values are compared to the values measured by the sensor of the MEX-system (see Fig. 54)

**Fig. 55:**
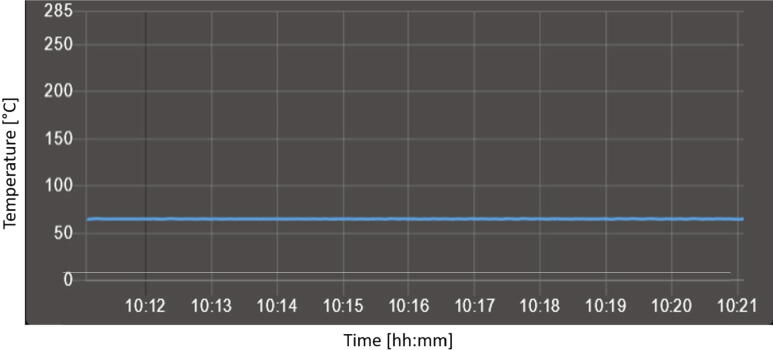
Timeline shown by the dashboard of Duet Web Control of the build platform temperature sensor with a target temperature of 60 °C. The minimum measured temperature was 59.7 °C and the maximum measured temperature was 60.4 °C.

The greatest offset between the commercial thermometer and the built plate temperature sensor is −2.1 °C with an average offset of −1,3 °C. Another important point to consider is the quality of the control. For this purpose, a target temperature of 60 °C was set and the timeline of the temperature is recorded (see Fig. 55) over ten minutes. Then the occurring maximum and minimum values are determined.

**Fig. 56:**
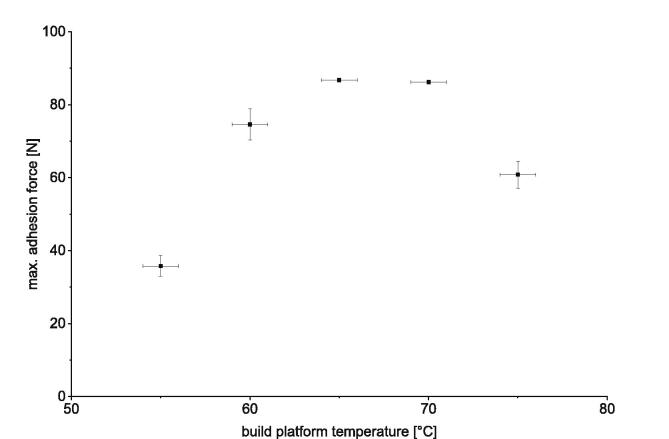
Correlation of the determined adhesion force and the set build platform temperature. For this series PLA was printed to a brass sheet. Following printing parameters are used: print speed 10 mm/s; first layer height 0.5 mm; layer height 0.5 mm; nozzle temperature 230 °C; nozzle diameter 0.8 mm. The error bars show the standard error of the mean calculated out of 15 measurements for each data point.

To determine if the offset and variation are critical the correlation of adhesion force and the build platform temperature must be known. Therefore, a series of adhesion tests with different build platform temperatures should be done. An example is shown in Fig. 56. For this measurement series polylactide (PLA) was printed with build platform temperatures from 55 °C to 75 °C. M. Spoerk [10], [12] and M. Kujawa [11] published several articles describing the same issue. The correlation between build platform temperature and adhesion force measured here is in line with the results of M. Spoerk and M. Kujawa.

Whether a deviation of 2 °C in the build platform temperature also means a significant deviation in the measured adhesion force depends on how close one is to the optimum. In this example the maximum adhesion force could be measured for a build platform temperature of 65 °C to 70 °C. A deviation of 2 °C in the build platform temperature means a deviating adhesion force of around one Newton. At the edge of the tested temperature interval, a deviation of 2 °C is clearly more critical. Here, the measured adhesive forces can deviate by up to 5 N. Therefore, it is recommended to determine the optimal platform temperature for the subjected combination of print material and build surface at first. The optimal build platform temperature in this context is the temperature with the highest adhesion between part and build surface.

**Fig. 57:**
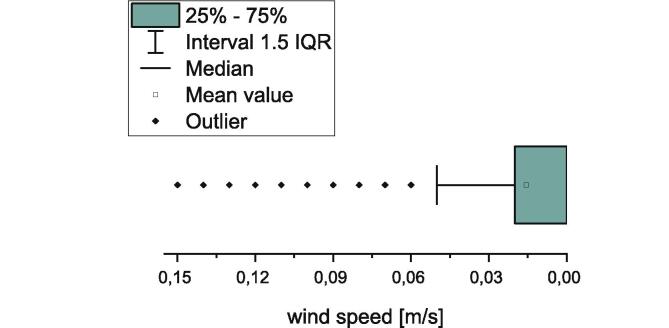
Boxplot of the measured wind speeds at the installation site of the measurement device.

The process temperatures can be influenced by air movement. For this reason, a measurement of the air movements at the installation site of the adhesion measurement device was done with a thermal anemometer type Testo 405 i. The wind speeds were measured every-two seconds over an interval of 40 min. All measured values were displayed in a boxplot (see Fig. 57).

At the installation site, wind speeds between 0 m/s and 0.15 m/s were measured. The average value is 0.015 m/s with a standard deviation of 0.02 m/s. Therefore, all of the measured values are in the order of magnitude of the accuracy of the thermal anemometer which is 0.1 m/s. Due to this, an influence of air movement onto the results measured by the adhesion measurement device is not to expect. If the measurement device gets installed in an air-conditioned room with heavy air movement, an enclosure is recommended

### Axis accuracy

The measurement device has no dedicated sensor which measures the displacement or its speed. The Duet 3 6HC has no feedback from the motors and sends only impulses to the motor according to the required speed and distance. In the following section the error of the displacement and speed is measured.

**Fig. 58:**
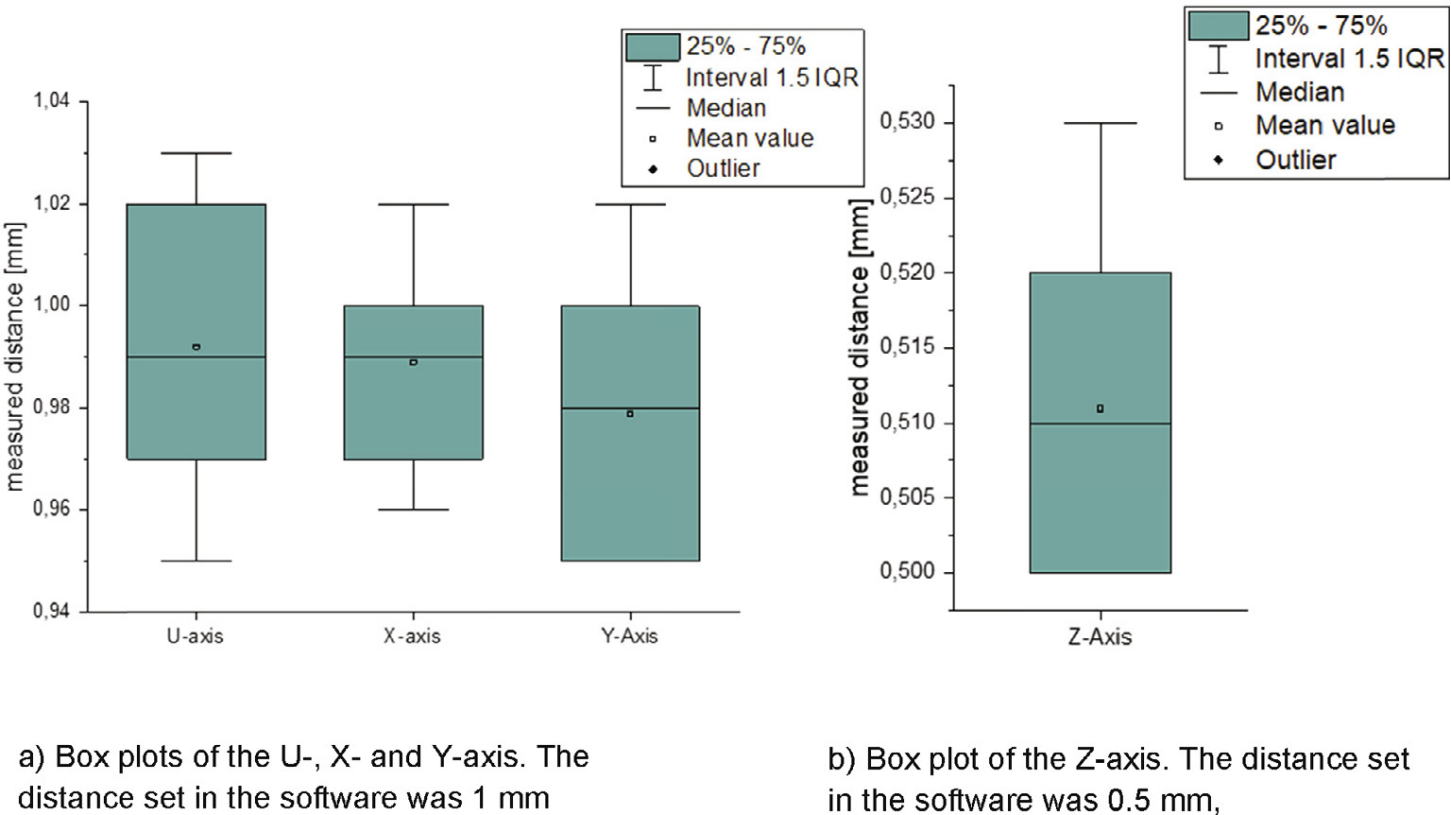
Box plot of the measured distances for every axis.

To determine the error of the positioning, an axis is moved via Duet Web Control a certain distance. The distance actual driven is measured using a dial gauge and compared to the one chosen in Duet Web Control. For each axis of the MEX-system and the one of the measurement unit (called U-axis) ten measurements were done. The U-, X- and the Y-axis were moved 1 mm in Duet Web Control and the Z-axis was moved 0.5 mm which is the layer height of all following printing tests. The results are shown in Fig. 58.

The mean value is 0.99 mm for the X- and U-axis and 0.98 mm for the Y-axis. This difference between measured distance and the one set in the software is nearly as big as the measurement error of the dial gauge which is 0.01 mm. This also applies to the Z-axis. Due to an error in the order of magnitude of 0.01 mm or below the positioning is considered as precise enough.

**Fig. 59:**
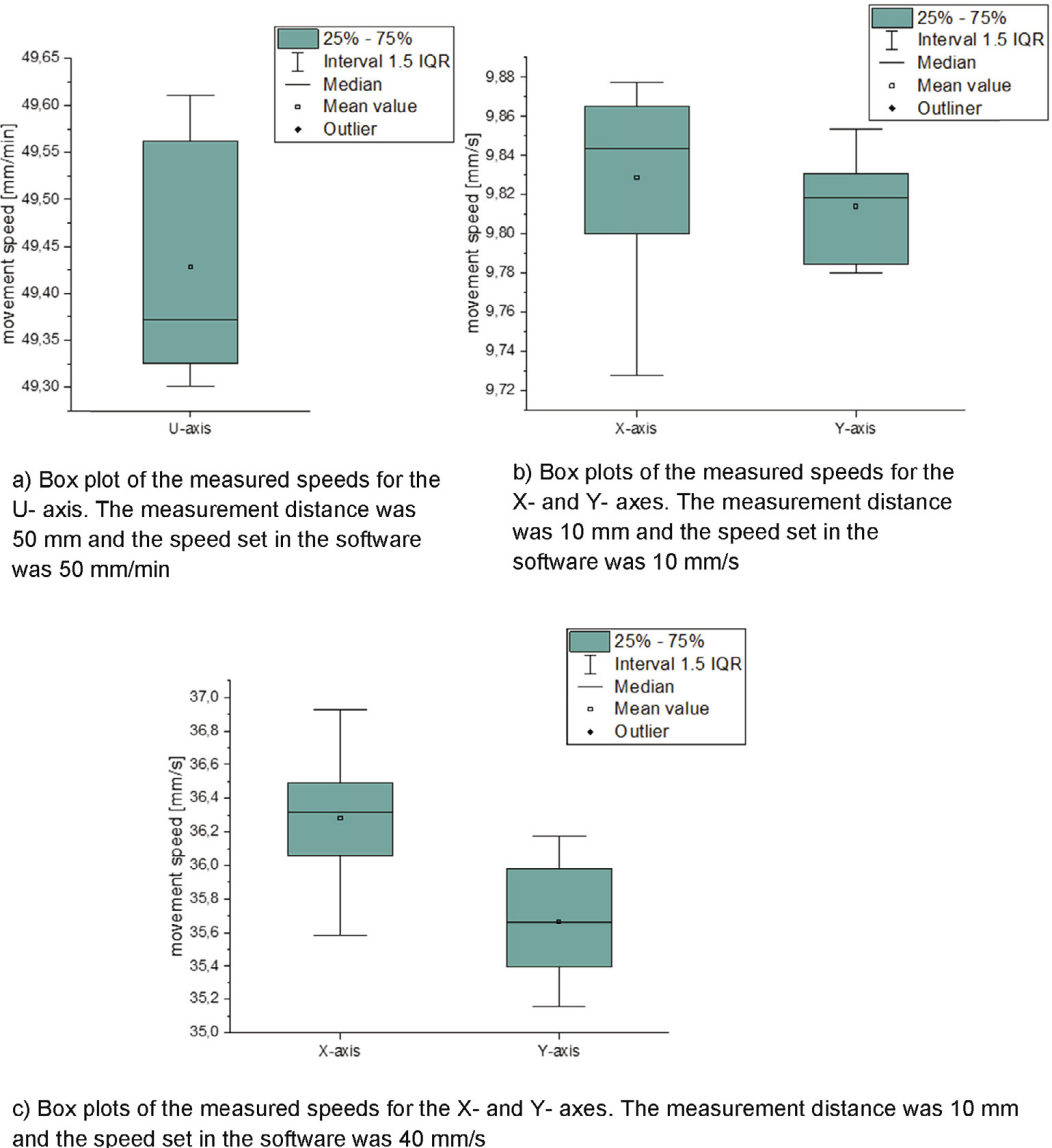
Box plots of the measured movement speed for the U-, X- and Y- axes.

In the next step the error of the axis movement speed was determined for the U-, X- and Y-axis (see Fig. 59). The error of the Z-axis speed is not controlled, because the speed of this axis is not relevant for the measurement procedure or the printing process.

To determine the error, the axes were moved with a software command. This command sets the distance and the speed for the movement. The time for the movement speed was measured, a speed was calculated and compared to the speed set in the software. For the time measurement a python script was used. Due to a HTTP connection the script is able to send the movement commands to the MEX-system and receive the confirmation that the movement has been executed.

Due to the test speed prescribed by DIN EN 28510–1 the error of the U-axis was only determined for a speed of 50 mm/min. The movement speed error of the X- and Y- axes were measured for 10 mm/s and 40 mm/s which is the interval used in the following tests.

According to DIN EN 28510–1 the speed of the measurement unit must be 50 mm/min ± 5 mm/min. As shown in Fig. 59a) the mean value is 49.43 mm/min with a standard deviation of 0.12 mm/min. Therefore, the requirements of DIN EN 28510–1 are fulfilled.

For a speed of 10 mm/s set in the software a mean value of 9.83 mm/s for the X-axis and 10.12 mm/s for the Y-axis was measured. The standard deviation for the X-axis is 0.05 mm/s and 0.03 mm/s for the Y-axis.

At a speed of 40 mm/s the difference between the measured speed and the one set in the software gets bigger. For the X-axis a value of 36.28 mm/s with a standard deviation of 0.38 mm/s and for the Y-axis a value of 35.67 mm/s with a standard deviation of 0.34 mm/s was measured. These differences are caused by the acceleration of the axis. This effect takes stronger into account the higher the printing speed is.

### Z-probe

The BL-Touch z-probe is used to make sure that the thickness of the first layer is reproducible. To determine the precision of the sensor 225 printing tests consisting of a single plastic strand were printed and the thickness measured with a micrometer screw. The targeted thickness was chosen in the slicer with a value of 0.5 mm. For each test the distance between the nozzle and the build platform was set with the z-probe.

**Fig. 60:**
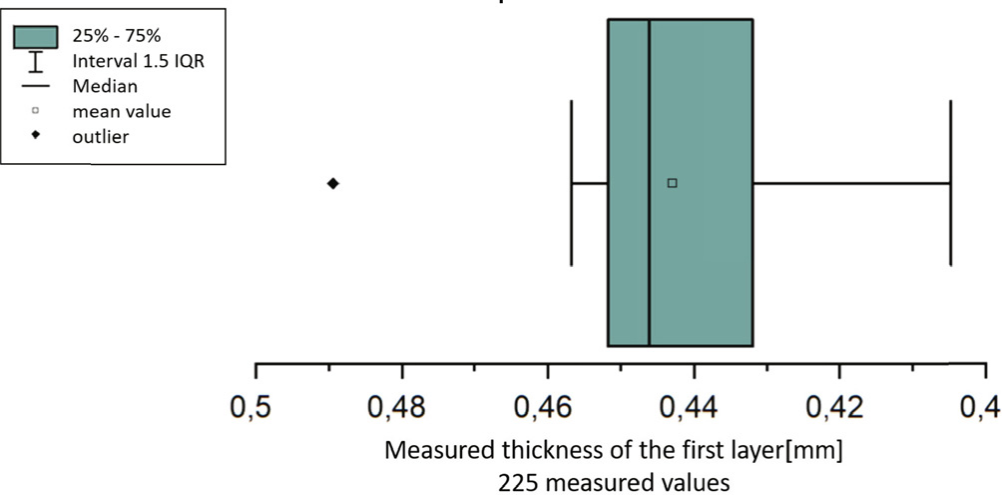
Box plot of the measured first layer thickness of 225 printing tests.

**Fig. 61:**
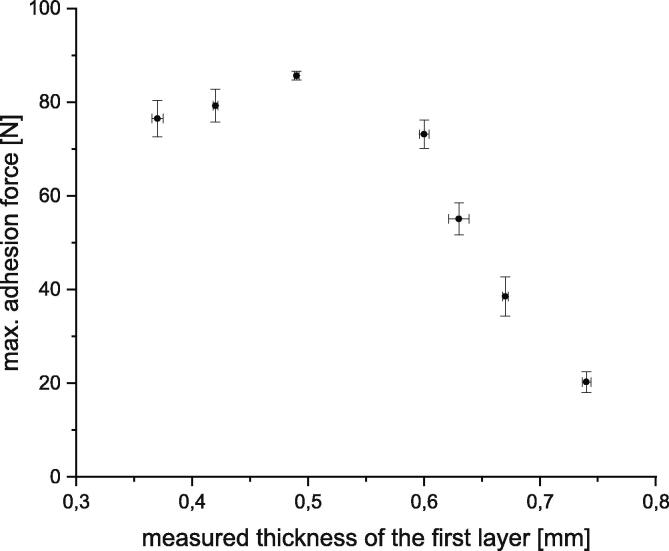
The correlation between the measured first layer thickness and the measured adhesion force (each data point is the average of 15 single measurements). For this series Polylactide was printed to a brass sheet. Following printing parameters are used: print speed 10 mm/s; first layer height 0.5 mm; layer height 0.5 mm; nozzle temperature 230 °C; nozzle diameter 0.8 mm. The error bars show the standard error of the mean calculated of 15 measurements for each data point.

The results are shown in a box-plot in Fig. 60. It can be seen that the measured thickness has a variation of around 60 µm. To determine if this variation is acceptable a series of adhesion tests was done with measured first layer thicknesses from about 0.35 mm up to 0.75 mm. Such series are also shown in [12], [11]. It should be noticed that in both series only the value chosen in the slicer-software was viewed. The real printed thickness was, in contrast to the series shown in Fig. 61, not measured. In Fig. 61 can be seen that the effect of a variation of 60 µm depends on how close the measured thickness to the value set in the slicer is. The bigger the difference the bigger is the impact onto the measured adhesion force. The variation of the adhesion in the above measured interval between 0.4 mm and 0.46 mm is ca. 5 N.

**Fig. 62:**
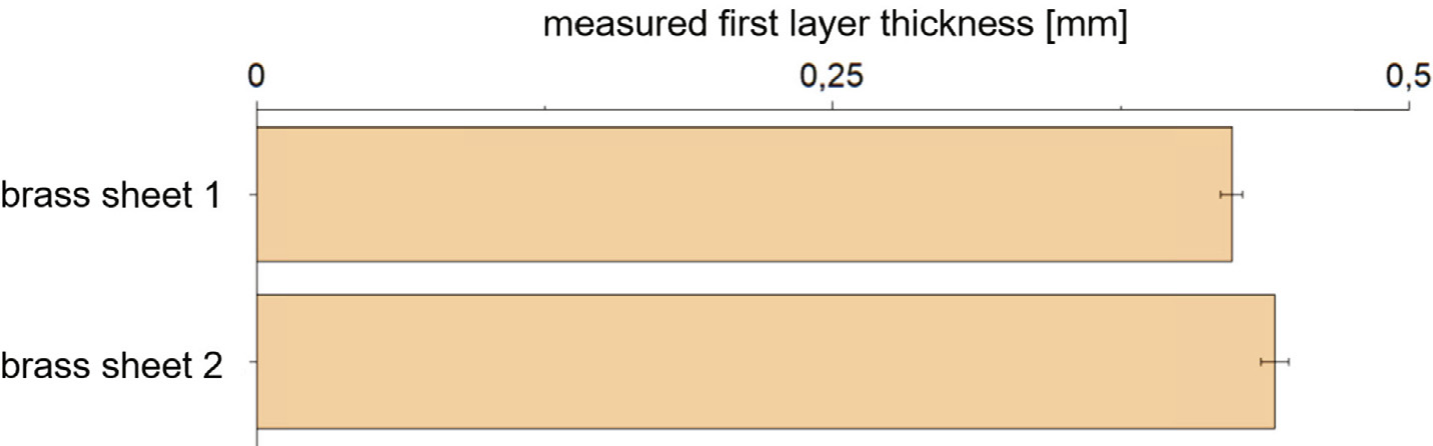
Measured first layer thicknesses printed on two different brass sheets. For those measurements PLA was printed on CuSn37 sheets with following print parameters: 0.5 mm first layer height, 0.5 mm layer height, 0.8 mm nozzle diameter, 60 °C build platform temperature, 230 °C nozzle temperature, 10 mm/s print speed. The error bars show the standard error of the mean calculated of 15 measurements each.

To determine how good the z-probe sensor can handle a change of the build surface, two brass sheets with the same geometry were tested. 15 specimens consisting of a single strand with rectangular shape were printed on top of each brass sheet and their thickness was measured. The average values (see Fig. 62) are 0.43 mm on brass sheet number one and 0.46 mm on brass sheet two. Thus, shows that the z-probe sensor works properly and allows to print repeatable first layers. According to Fig. 62 it can be seen that such small variations do not have a significant influence to the measured adhesion force.

### Force sensor

**Fig. 63:**
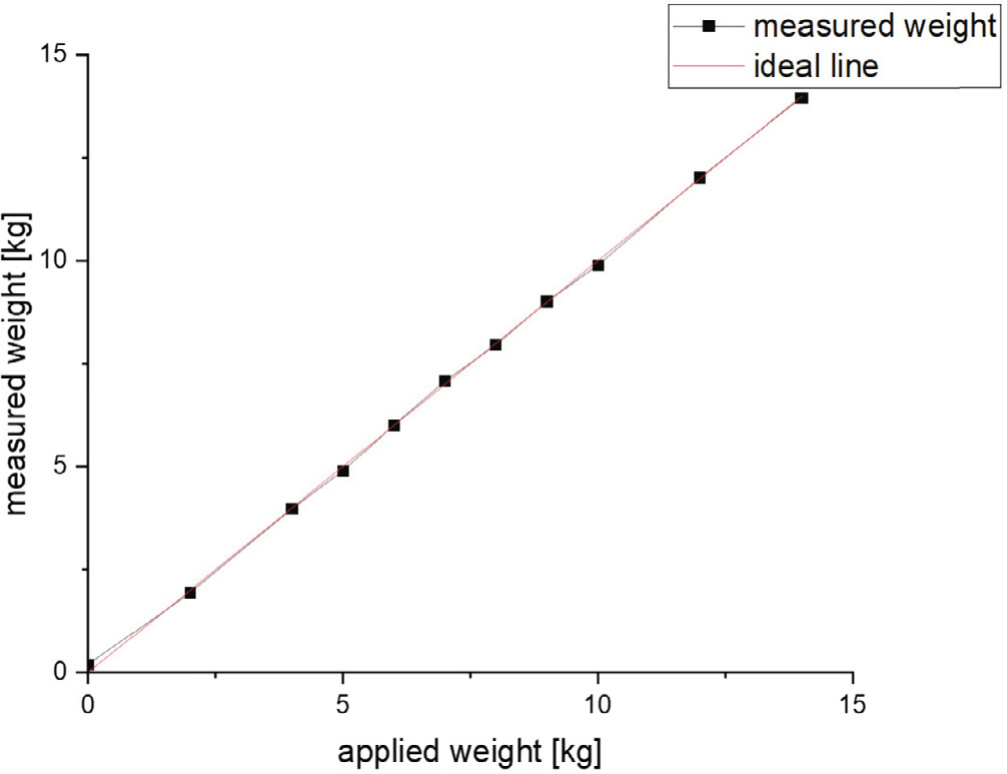
The applied weight plotted versus the measured weight. The red line shows the ideal. (For interpretation of the references to color in this figure legend, the reader is referred to the web version of this article.)

To determine the quality of the force measurement, weights were attached to the load cell and measurement cycles were run with the measurement program. The measured force is divided by 9.81 N/kg to convert it to a corresponding weight. Fig. 63 shows the measured weights in comparison to the applied weights. It can be seen that they match except for ca. ± 0.2 kg by an average difference of 0.58 percent. As mentioned before, the measurement device is based on DIN EN 28510–1 which demands an accuracy of at least 5 percent of the force measurement. Therefore, the determined quality is acceptable.

### Influence of time

**Fig. 64:**
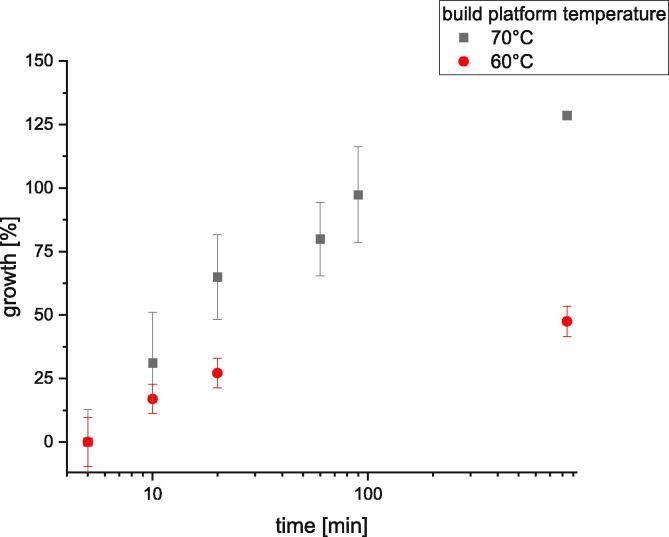
The influence of time between finish of the print of the specimen and the peeling test. The specimens were printed out of PLA with a nozzle temperature of 230 °C, a printing speed of 10 mm/s, a layer height of 0.5 mm and a nozzle diameter of 1 mm. The error bars show the standard error of the mean calculated of 5 measurements for every data point.

To determine if the time between the completion of the print of a specimen and the start of the measurement cycle has an influence on the adhesion force the following series were measured. For each data point shown in Fig. 64 15 specimens were printed out of PLA onto a brass sheet and after a defined time elapsed their adhesion was measured. A time of five minutes is set as standard. The percentage increase of the measured adhesion force with regard to the standard at 5 min is shown in Fig. 64. It can be seen that the time has a significant influence on the measured adhesion force. After a timespan of twenty minutes the adhesion force has already increased by 60 % at 70 °C. In order to ensure comparable measurement results, there should always be an equal period of time between the end of the printing process and the start of the measurement.

### Influence of printing position

**Fig. 65:**
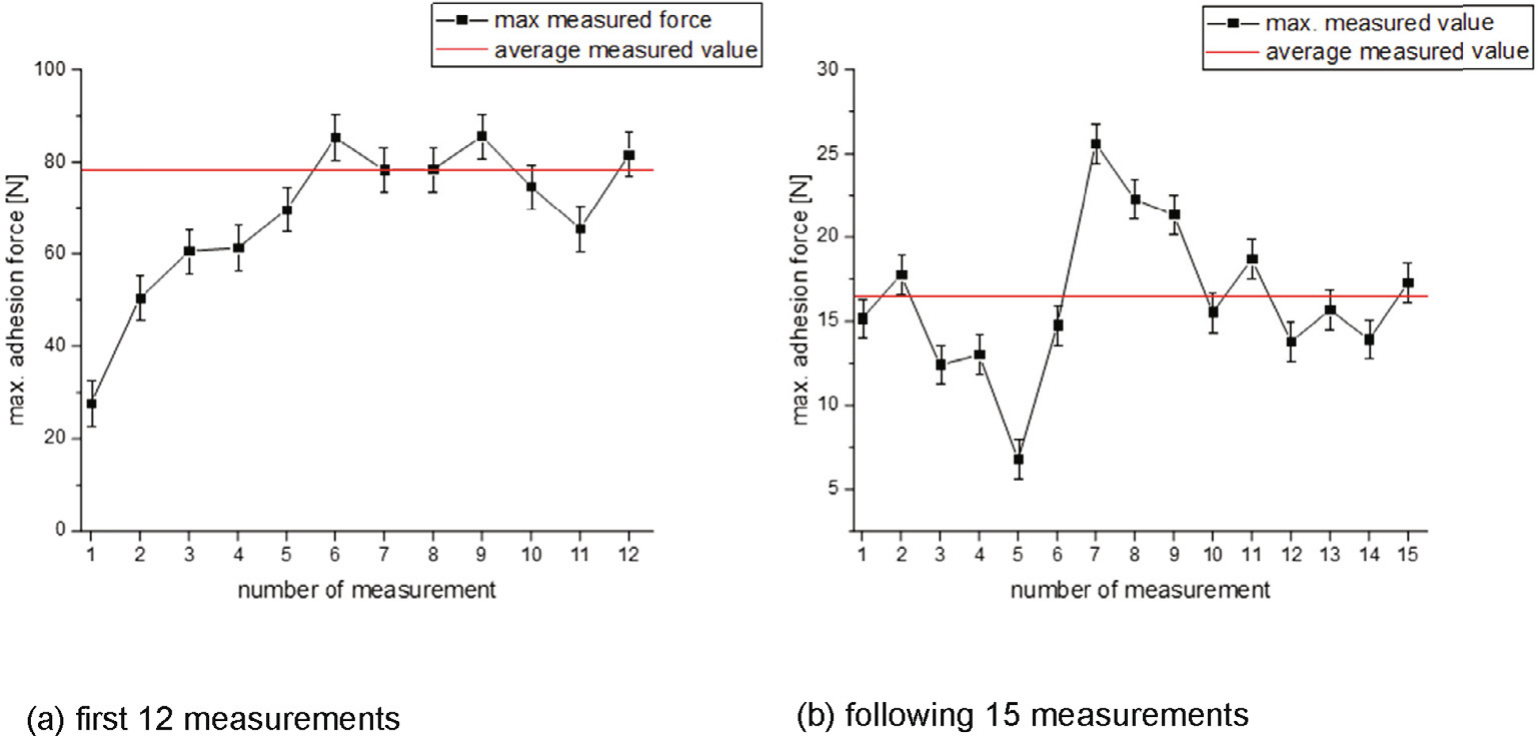
Influence of the frequency with which printing has already been made to a position. For those measurements Colorfabb XT Transparent filament was printed on CuSn37 sheets with following print parameters: 0.5 mm first layer height, 0.5 mm layer height, 0.8 mm nozzle diameter, 250 °C nozzle temperature, 10 mm/s print speed build platform temperature: a) 100 °C and b) 80 °C. The error bars show the standard error of the mean. The mean was calculated out of all 12 respective 15 measurements.

To determine if residues remain on the build platform after a measurement and if applicable they have an influence on the measurement 12 specimen were printed on the same position on top of a new brass sheet. The results are shown in Fig. 65.

**Fig. 66:**
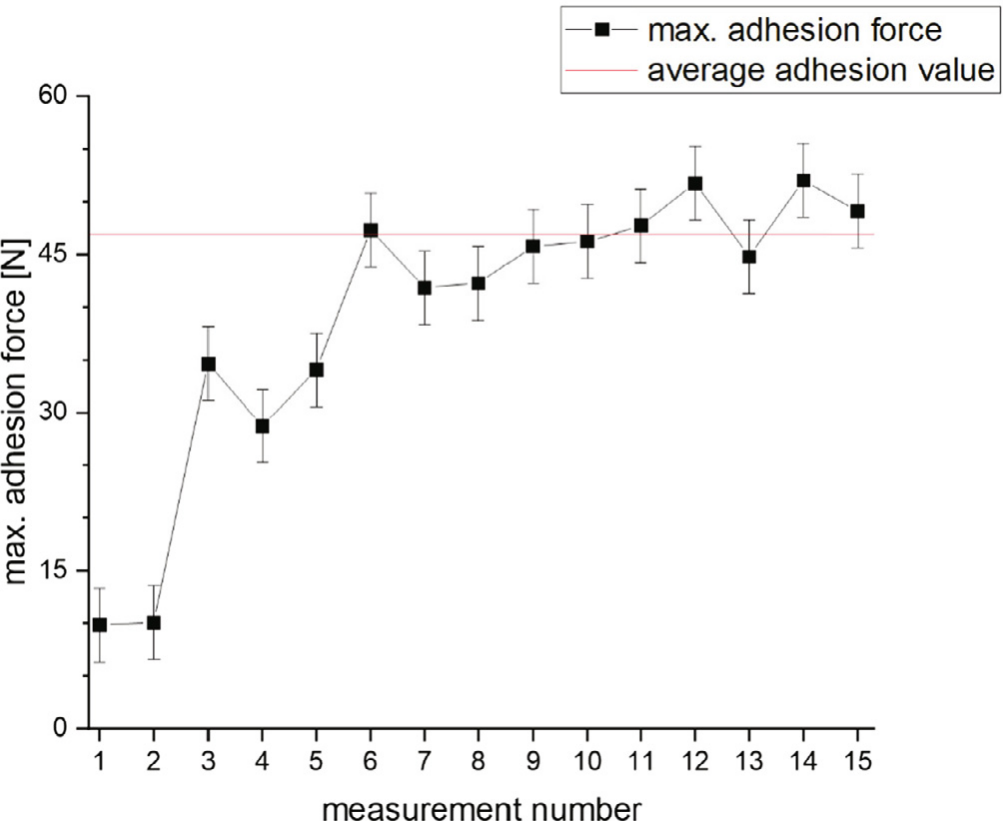
Influence of the frequency with which printing has already been made to a position. For those measurements Colorfabb XT Transparent filament was printed on CuSn37 sheets with following print parameters: 0.5 mm first layer height, 0.5 mm layer height, 0.8 mm nozzle diameter, 250 °C nozzle temperature, 10 mm/s print speed build platform temperature: 90 °C. The error bars show the standard error of the mean calculated out of all 15 measurements.

In Fig. 65a) one can see that the measured adhesion forces increase within the first six measurements and the following values scatter around an average. The measurements shown in b) are from a new measurement series taken after the one shown in a) but on the same position. In this series the values scatter around an average right from the beginning. The build platform was then turned 180° to print on a new position and the increase in the first few measured values was seen again (see Fig. 66). For a repeatable and comparable measurement, the first ten printed specimen should not be included into a measurement series.

### Repeatability and precision

In the following section the repeatability and precision of the adhesion measurement method and the corresponding device is discussed. At first was determined how a build platform change affects the measured adhesion forces. Second the dependency of the precision of the combination of build platform material and printing material was examined. A required precision is not yet defined in the current version of DIN EN 28510-1. In the following section always the maximum measured adhesion force of a single measurement is used to calculate averages or standard deviations.

**Fig. 67:**
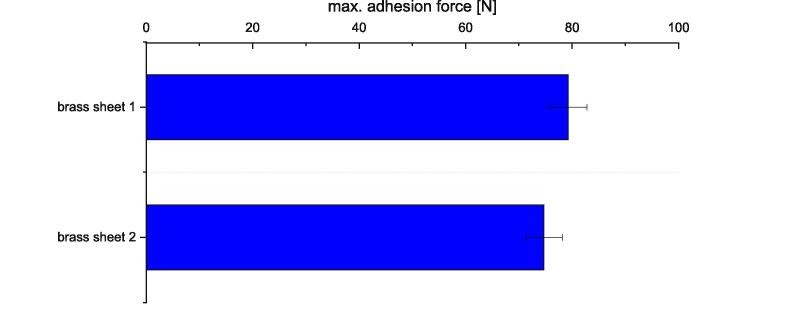
Comparison of the measuring results taken on two brass sheets. The specimens were printed out of PLA with a printing speed of 10 mm/s, a nozzle temperature of 230 °C, a build platform temperature of 60 °C, a nozzle diameter of 1 mm and a layer height of 0.5 mm. The error bars show the standard error of the mean calculated out of 15 measurements taken on each sheet.

To determine the repeatability of the adhesion measurement method, 25 specimens were printed onto a brass sheet (see Fig. 67), their adhesion was measured and as described above the first ten measurements were discarded. From the remaining 15 measurements the average of the maximum adhesion force was calculated and plotted in Fig. 67. The measurements series was repeated onto a second brass sheet.

The average maximum adhesion determined for brass sheet one is about 79.5 N and for the second brass sheet around 75 N. The variation is ca. 6 percent.

**Fig. 68:**
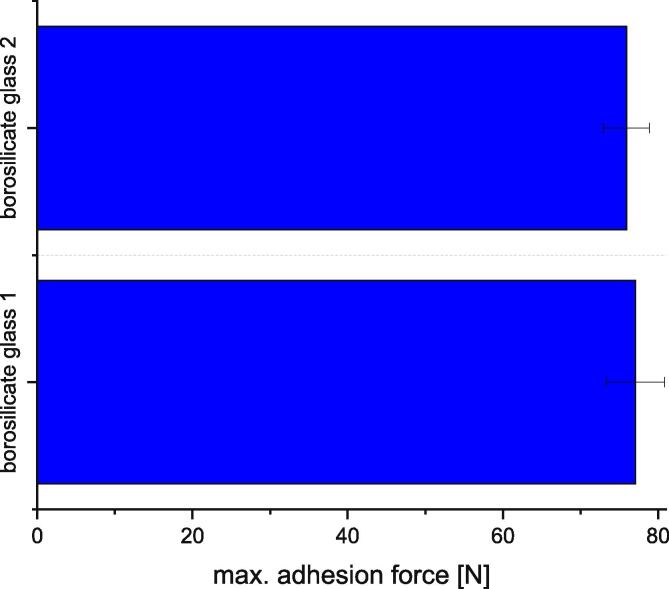
Comparison of the measuring results taken on two borosilicate glass sheets. The specimens were printed out of PLA with a printing speed of 10 mm/s, a nozzle temperature of 230 °C, a build platform temperature of 80 °C, a nozzle diameter of 1 mm and a layer height of 0.5 mm. The error bars show the standard error of the mean calculated out of 5 measurements taken on each sheet.

Also, for two borosilicate glass plates the variation was measured. Fifteen measurements were done and the first ten measurements discarded. The printing parameters are listed in Fig. 68.

For borosilicate glass 1 an average adhesion force of 77 N was calculated for five measurements. The measurement series for borosilicate glass 2 shows an average adhesion of 76 N which means that the variation is about 1.3 %. This shows that the variation of 6 percent does not apply to all combinations of printing material and build surface material. Therefore, the dependence of the variation from the used material combination should be determined.

Following is described how the precision is affected by changing the combination of build surface material and printing material. Nine different polymers were printed onto build surfaces made of borosilicate glass, Pertinax and brass. All specimens were printed with a layer height of 0.5 mm, a nozzle diameter of 1 mm and a print speed of 10 mms/s. The used nozzle temperatures and build platform temperatures are listed in Table 5.

**Table 5:** Printing parameters used for the measurement series to determine the dependency of the precision onto the combination of printing material and build surface material.

Plastic	Nozzle temperature [°C]			Build platform temperature [°C]		
	Brass	Pertinax	Borosilicate glass	Brass	Pertinax	Borosilicate glass
PMMA	250	230	250	130	102	102
PLA	230	220	230	60	70	50
PP	230	230	230	63	63	63
PETG	250	230	230	100	96	96
ABS	270	230	230	130	125	125
ASA	250	230	230	100	125	125
HIPS	230	230	230	100	107	107
PC	270	230	230	132	132	132
CPE-HG100	not tested	260	260	Not tested	105	105

To evaluate the precision of the adhesion testing device the standard deviation and the relative standard deviation were calculated for all tested material combinations. The relative standard deviation is related to the average of the maximum measured forces. Onto every build surface ten specimens with PLA were printed before the measurements with different material combinations and discarded as described above. This procedure was not repeated after changing the printing material but only after changing the build surface.

**Fig. 69:**
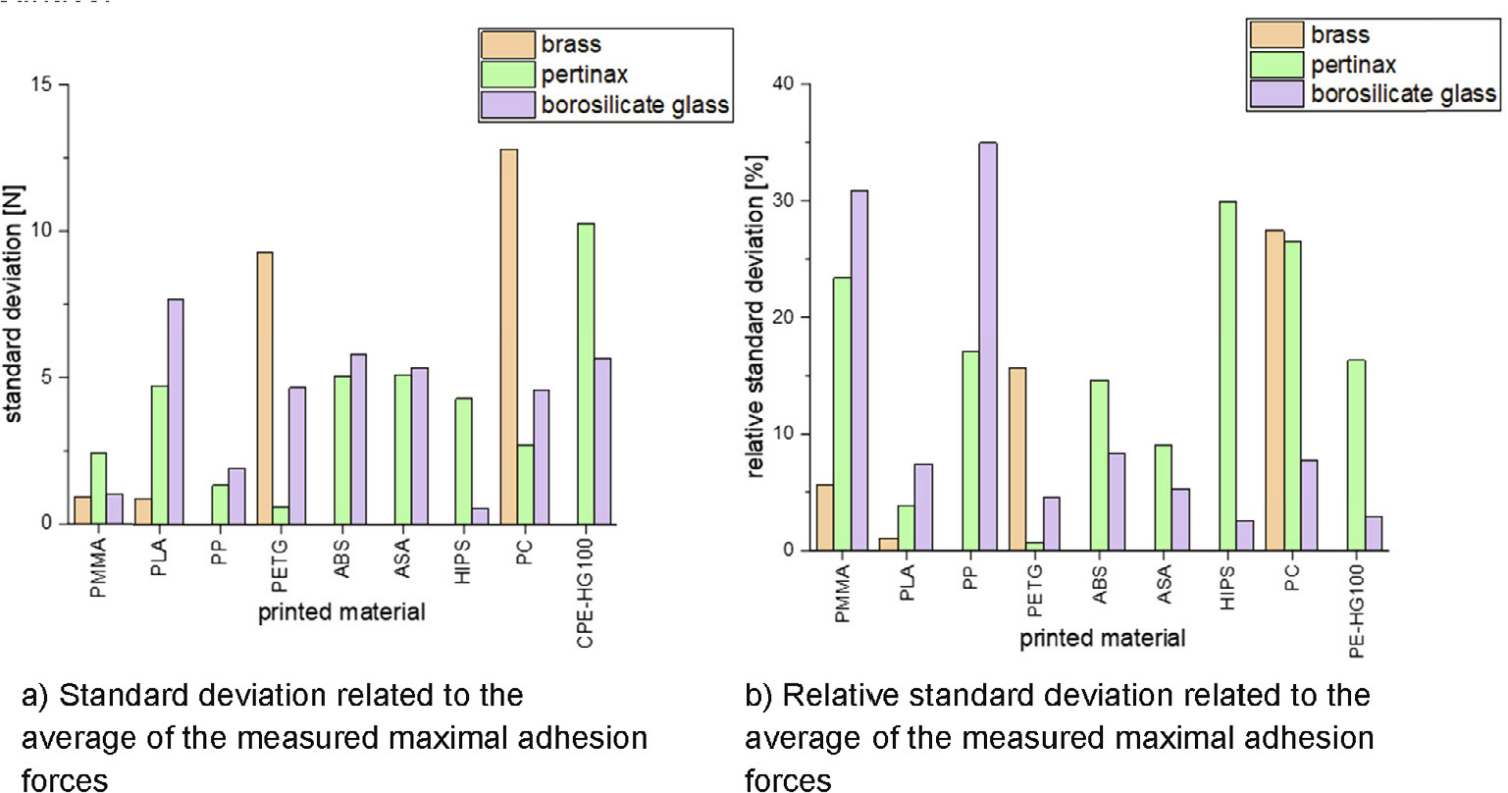
Standard deviation and relative standard deviation of all tested material combinations.

The standard deviations of the most material combinations are about 5 N (see Fig. 69). The highest standard deviation is calculated for polycarbonate printed onto brass with a value of 12.81 N while the lowest one is determined for HIPS printed onto borosilicate glass with 0.6 N. These standard deviations lead to high relative standard deviations when very small adhesion forces were measured. Such small forces occur on material pairings like PP on borosilicate glass or HIPS on Pertinax. The adhesion forces for the material pairing PP – borosilicate glass are about 4 N. Therefore, a deviation in the same order of magnitude lead to a high relative standard deviation of around 35 %. On the other hand, a standard deviation of 7.7 N for the pairing PLA – borosilicate glass with measured forces up to about 230 N leads to a comparatively small relative standard deviation of ca. 3 %.

In the last section there will be examined how the precision is affected by changing the process parameters. This is done for three different build surfaces: borosilicate glass, Pertinax and brass. All specimens of the following measurement series were printed out of PLA with a nozzle diameter of 1 mm and a layer height of 0.5 mm. The nozzle temperature was varied between 190 °C and 280 °C and the build platform temperature was set between 60 °C and 80 °C. The printing speed was chosen between 10 mm/s and 40 mm/s. The calculated standard deviation and relative standard deviation is related to the average formed out of the maximum measured adhesion forces of five measurements.

**Fig. 70:**
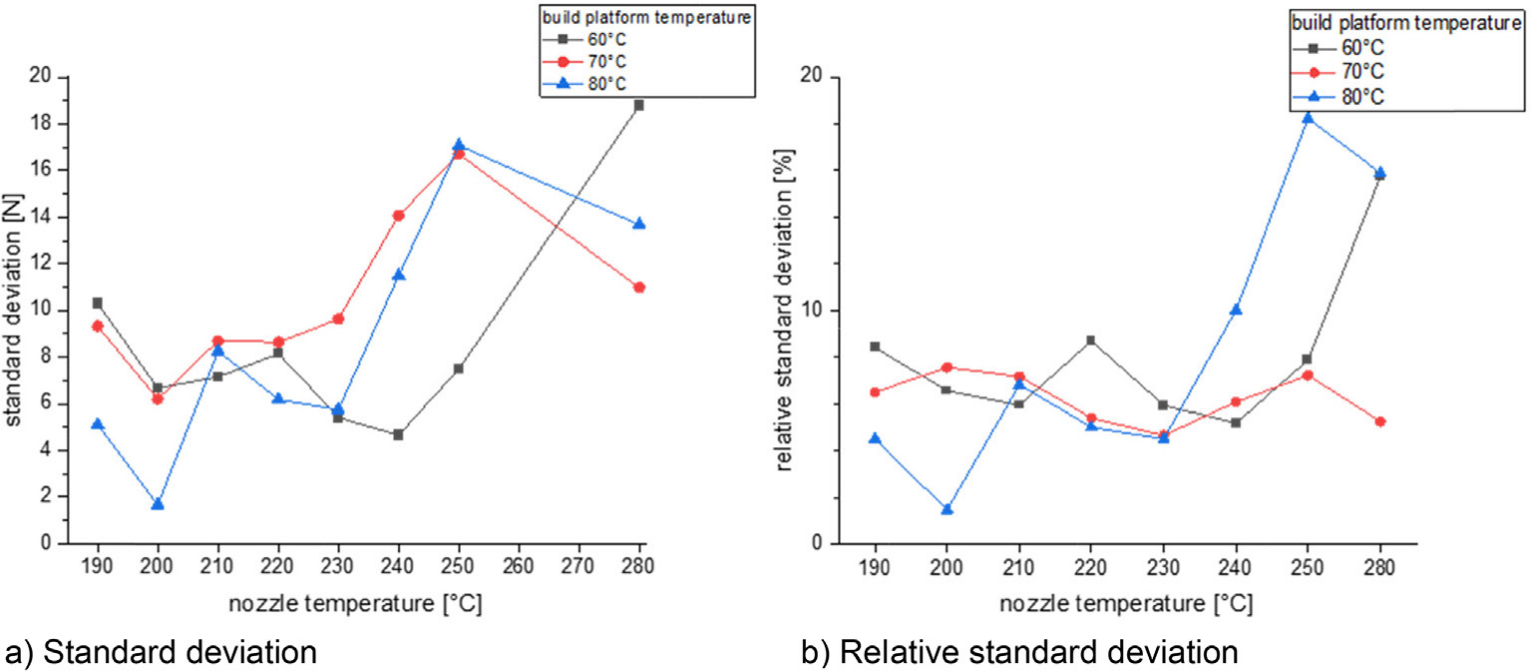
Standard deviation and relative standard deviation of specimens printed out of PLA onto borosilicate glass. The printing speed was set to 10 mm/s.

At first the influence of varying nozzle temperatures and build platform temperatures was determined for borosilicate glass as build surface material. The results are shown in Fig. 70.

It can be seen that higher nozzle temperatures tend to higher standard deviations and to higher relative standard deviations. For a build platform temperature of 80 °C and a nozzle temperature of 200 °C the standard deviation is 1.68 N with a relative standard deviation of 1.47 %. After increasing the nozzle temperature to 280 °C the standard deviation rises to 13.67 N with a relative standard deviation of 15.9 %. This trend is the same for the other tested build platform temperatures of 60 °C and 70 °C.

The span of calculated standard deviations reaches from 1.67 N to a maximum of 18.79 N. For the relative standard deviations, a minimum of 1.47 % and a maximum of 18.24 % was calculated.

**Fig. 71:**
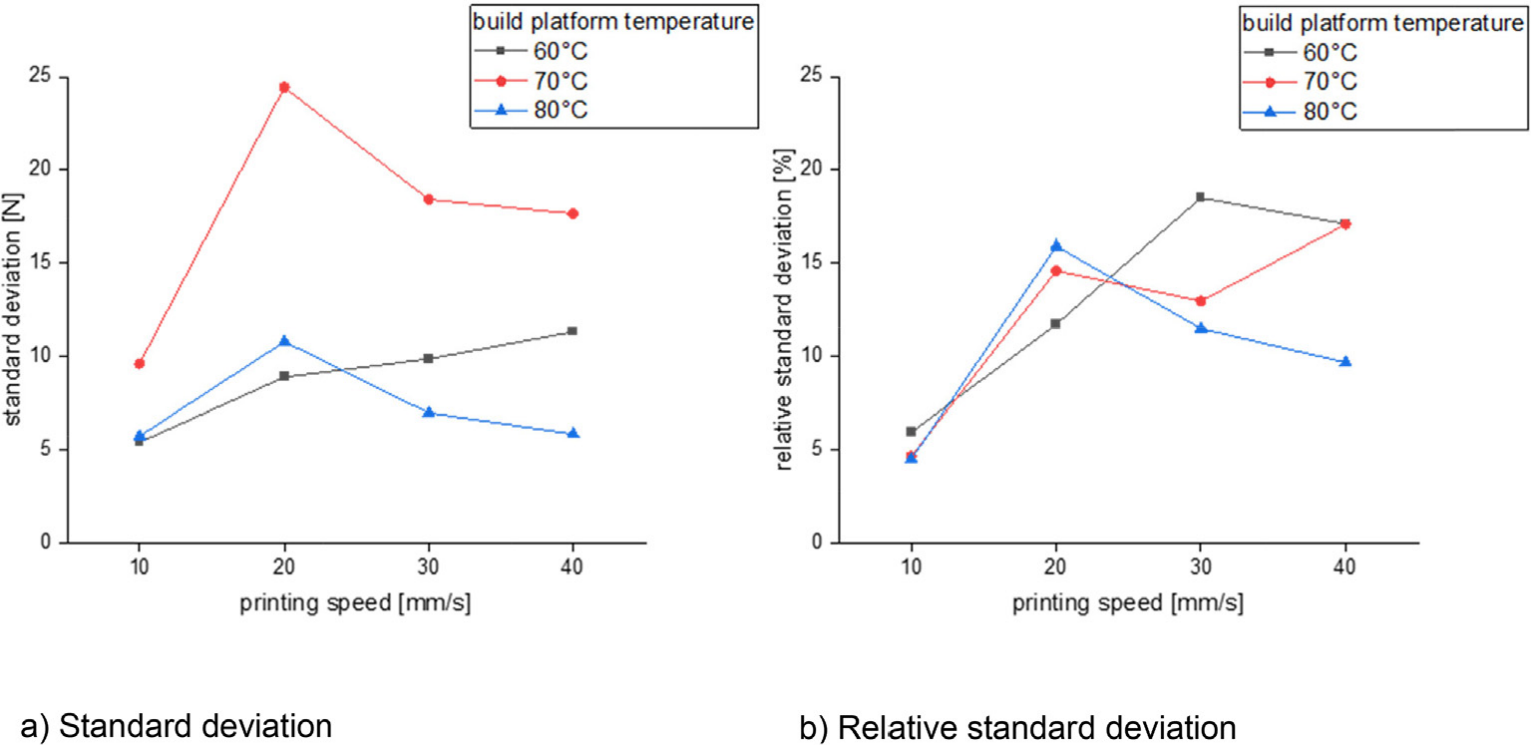
Standard deviation and relative standard deviation of specimens printed out of PLA onto borosilicate glass. The nozzle temperature was set to 230 °C.

Also, the influence of the printing speed onto the precision of the measurement was determined. The results are shown in Fig. 71.

It can be seen that the print speed has no influence onto the precision of the measurement. The standard deviation varies for a build platform temperature of 60 °C and 80 °C between 5.85 N and 13.31 N which is a difference of roughly 7 N. At a build platform temperature of 70 °C the difference between the highest and the lowest value is around 15 N. The maximum value is ca. 24.4 N and the minimum is ca. 10 N. For all three tested build platform temperatures the relative standard deviation moves in an interval between 5 % and 18.6 %.

**Fig. 72:**
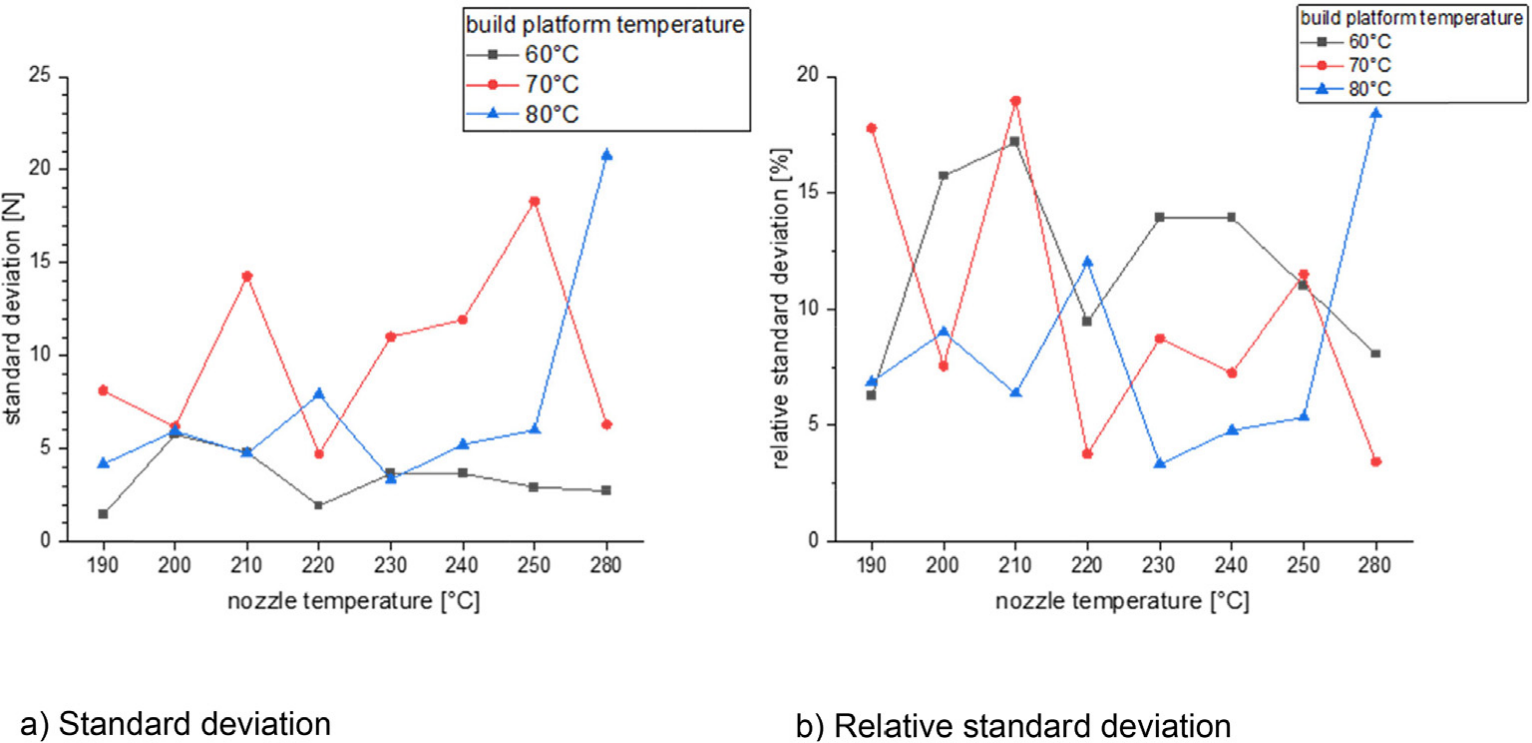
Standard deviation and relative standard deviation of specimens printed out of PLA onto Pertinax. The printing speed was set to 10 mm/s.

Following a measurement series was done to determine the correlation between the precision and the build platform temperature as well as the nozzle temperature onto Pertinax as build surface material. Fig. 72 shows the results.

In contrast to borosilicate glass no clear correlation between the nozzle temperature or the build platform temperature and the precision was found. For a build platform temperature of 60 °C the standard deviation shows no abrupt jumps and moves in an interval between 1.45 N and 6.47 N over the tested nozzle temperatures. A different behavior was observed for build platform temperature of 80 °C: The standard deviation moves in an interval between 3.34 N and 7.91 N for nozzle temperatures between 190 °C and 250 °C. A further increase of the nozzle temperature leads to a strong increase of the standard deviation to 20.76 N.

Also, no clear correlation between the nozzle temperature or the build platform temperature on the one hand and the relative standard deviation on the other hand was found. The relative standard deviation moves between a minimum of 3.43 % and a maximum of 18.4 %.

In the next measurement series, it was investigated if a correlation between the standard deviation or the relative standard deviation and the printing speed exists.

The standard deviation varies between a minimum of ca. 3 N and a maximum of 10.5 N. The difference between minimum and maximum is ca. 7.5 N which is in the same order of magnitude as to the one determined for borosilicate glass. As already described for borosilicate glass no correlation between the printing speed and the standard deviation can be found.

**Fig. 73:**
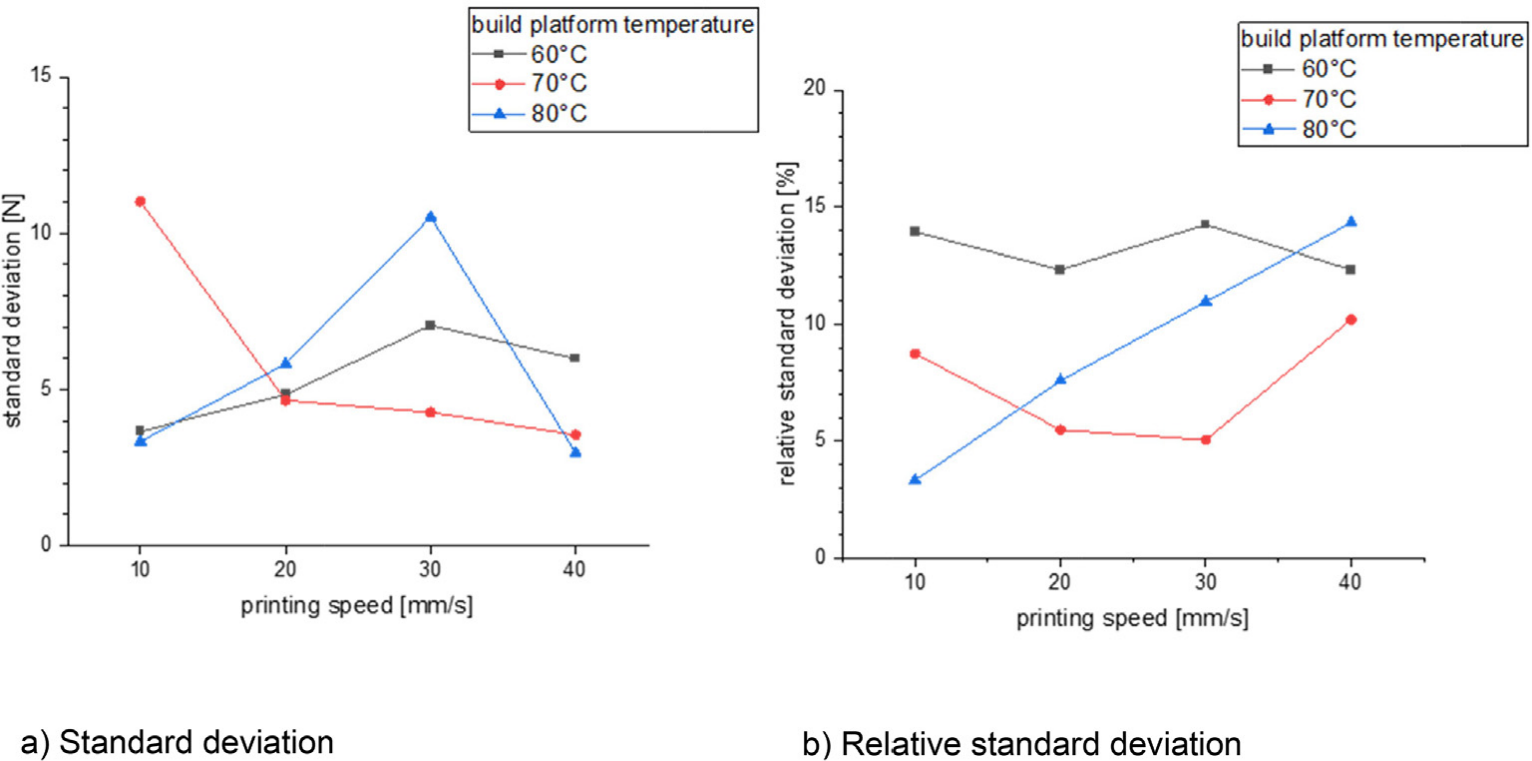
Standard deviation and relative standard deviation of specimens printed out of PLA onto Pertinax. The nozzle temperature was set to 230 °C.

Also, the relative standard deviation moves in a similar interval as calculated for borosilicate glass as build surface material. For Pertinax a minimum of 3.33 % and a maximum of 14.32 %. The difference between minimum and maximum is ca. 11 % and is nearly as big as the one calculated for borosilicate glass (ca. 9 %). In contrast to borosilicate glass, the minimum and maximum are 6 % respectively 4 % lower. A general statement about the correlation between printing speed and relative standard deviation cannot be made. Each curve shown in Fig. 73b) shows another correlation between build platform temperature and relative standard deviation.

**Fig. 74:**
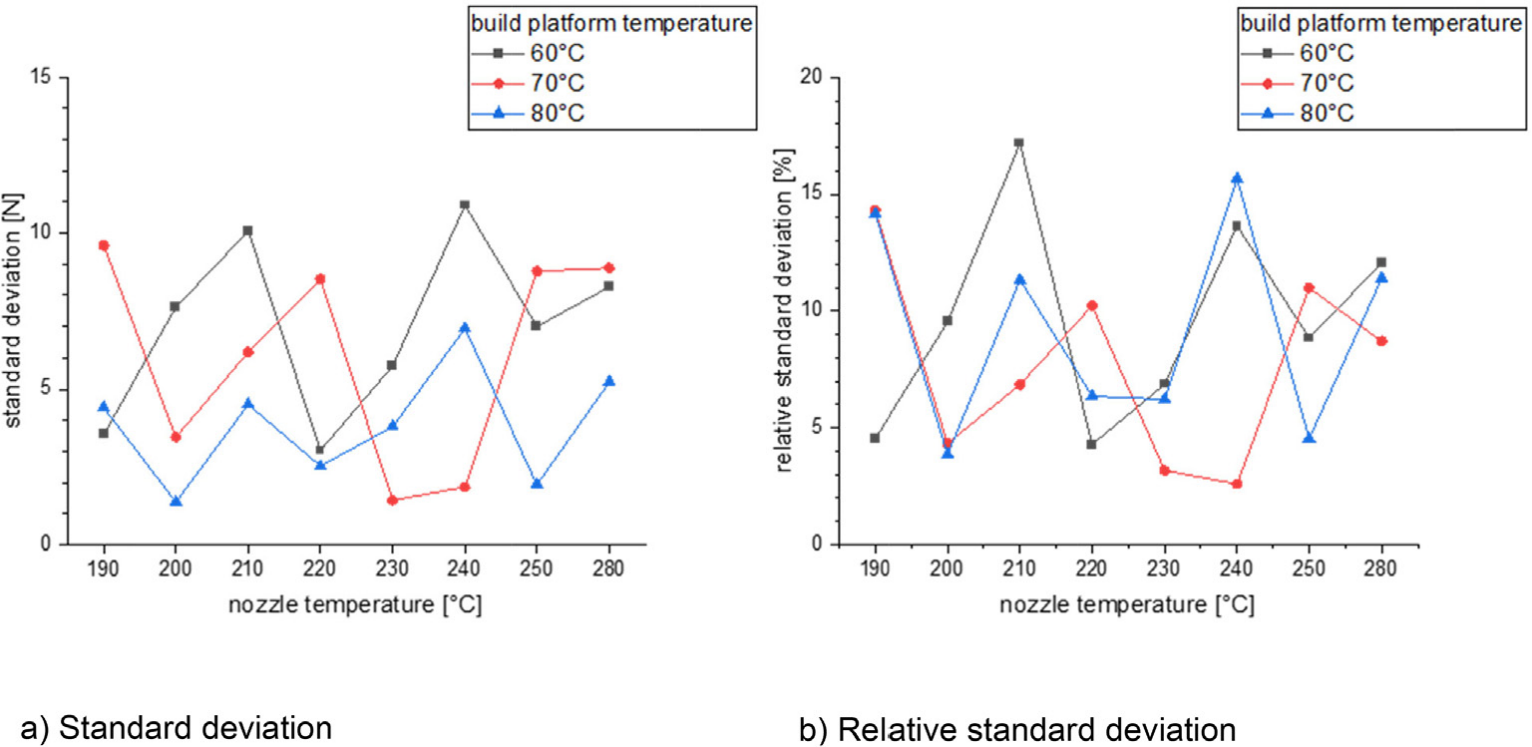
Standard deviation and relative standard deviation of specimens printed out of PLA onto brass. The printing speed was set to 10 mm/s.

In the following measurement series, it was determined which correlations between the nozzle temperature, the build platform temperature and the precision exist. The results are shown in Fig. 74.

No correlation between the nozzle temperature, the build platform temperature and the standard deviation can be found. The standard deviation moves in an interval between 1.37 N and 12 N. Also, for the relative standard deviation no correlation was found. The minimum relative standard deviation moves between a minimum value of 2.6 % and a maximum of 17.17 %.

**Fig. 75:**
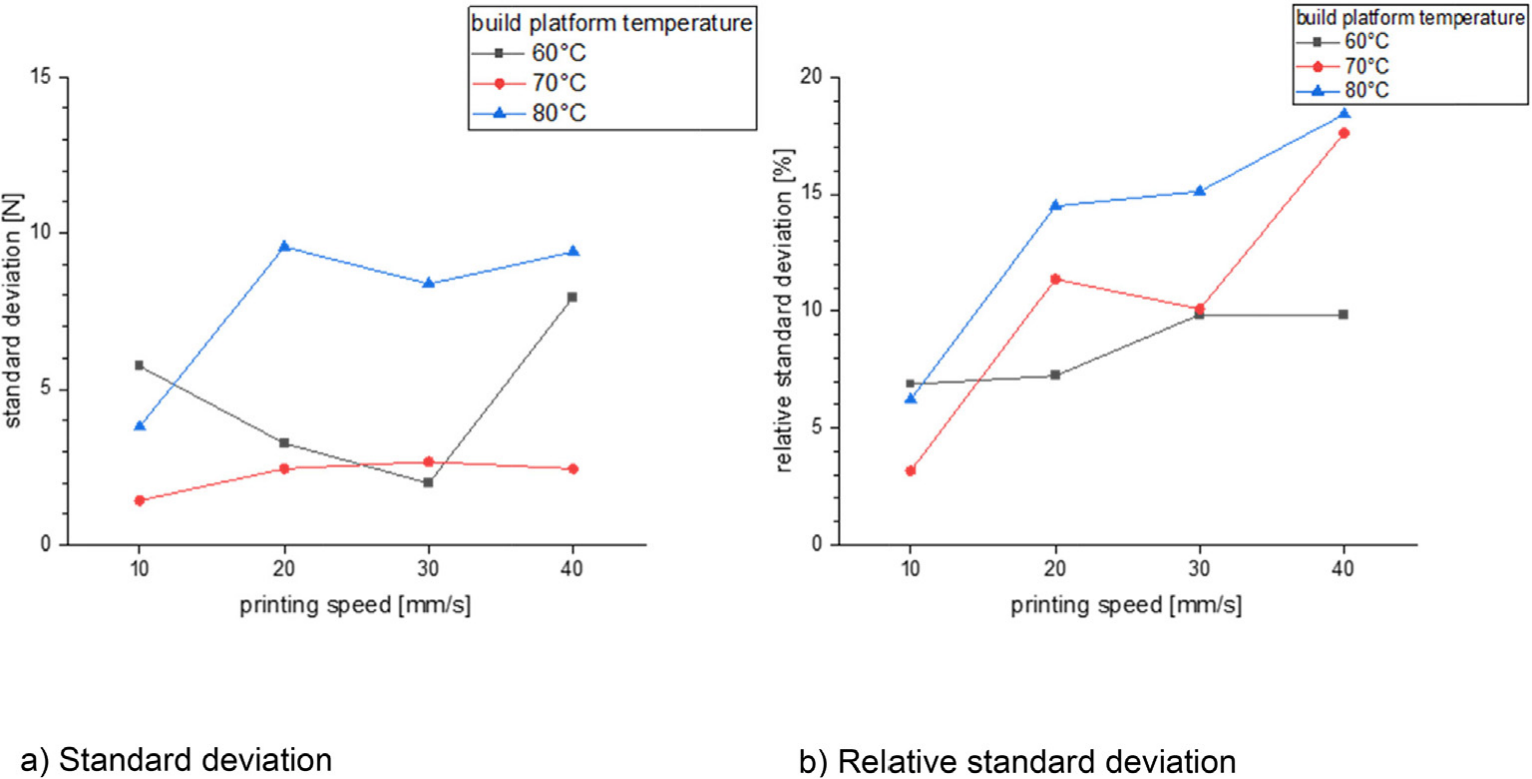
Standard deviation and relative standard deviation of specimens printed out of PLA onto brass. The nozzle temperature was set to 230 °C.

In the last measurement series, it was investigated if a correlation between the printing speed and the precision exists for brass. The results are shown in Fig. 75.

The standard deviation moves in an interval between a minimum of 1.43 N and a maximum of 9.56 N. The difference between minimum and maximum is ca. 8 N and is similar to the ones described for borosilicate glass with a value of about 15 N and Pertinax with a value of 7.5 N. A correlation between printing speed and the calculated standard deviation was not found.

In contrast to that, a correlation between printing speed and relative standard deviation was found. The higher the printing speed is the higher is the relative standard deviation. This can be explained due to the fact that the adhesion forces are reduced by an increasing printing speed. For a build platform temperature of 60 °C and printing speed of 10 mm/s an average adhesion of about 74 N was measured which decreases to 20.61 N for a printing speed of 30 mm/s. The standard deviation is not affected by the increasing printing speed and is therefore the relative standard deviation rises.

In the section below, possible factors are listed which could cause the majority of the variance.

The filament was first dried (storing for 4 h at 40 °C) and then stored in a Polymaker PolyBox Edition II. This should ensure a constant moisture of the print material. Despite this procedure a variation in the moisture could occur which may result in a measurement error.

Another possible source for measurement errors are the changing environmental conditions. The measurement device was not built in a laboratory with controlled humidity and temperature. If a room with standardized climatic conditions is available, the device should be preferably positioned there.

A contamination of the build surfaces cannot be excluded. To avoid this, the handling was done wearing gloves. Cleaning was only done with oil- and water-free pressured air due to the fact that the influence of solvents like water, acetone or isopropanol is yet unknown.

Also, a time dependent behavior of the build surface is possible, for example due to oxidation of the brass sheets.

### Use case scenario and outlook

**Fig. 76:**
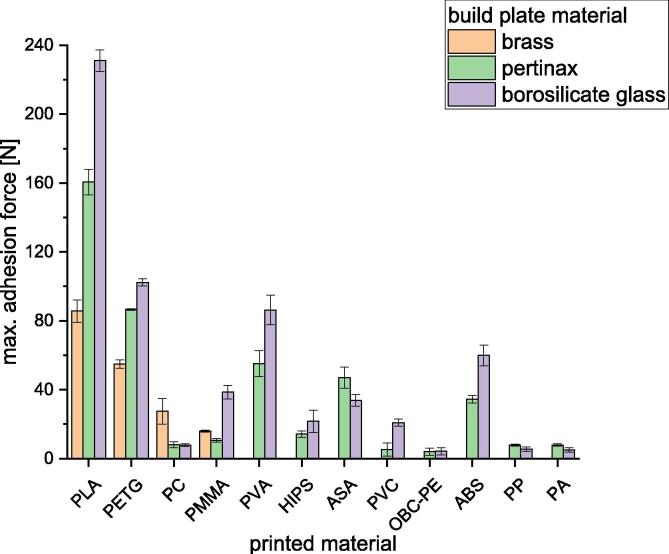
Correlation between the printed plastic and the material of the build surface. No value means that no adhesion between filament and build surfaces occurred and therefore no specimen could be printed. The error bars show the standard error of the mean calculated out of 5 measurements for each material.

A use case for the adhesion testing device is to find the best build surface for a filament. As mentioned in the introduction the adhesion of the part during the fabrication plays a major role and must be secured over the whole process. One of the easiest ways to achieve a sufficient adhesion is to choose a suitable build surface for the respective filament. In this case the process parameters do not need to be highly optimized and despite a good adhesion can be maintained. To show how the testing device can help to choose a suitable build surface for different materials, several test series were done. A variety of filaments were printed on top of three different build surfaces after ten prints with PLA were done and discarded. Five minutes after finishing the print of the specimen a peel test was done and the occurring forces were recorded. Each filament was printed at the parameters obtained by the corresponding manufacturer. In Fig. 76 the observed maximum forces for each build surface and filament are plotted.

The build surfaces can be compared according to the number of polymers which can be printed on or the maximum adhesion force that can be achieved.

The most polymers are compatible with Pertinax. Adhesion occurs with every tested polymer although the strength of the bonding varies very strong. The maximum adhesion force was measured for PLA with a value of 160 N while polypropylene (PP) only adheres with a maximum of 7.8 N. Due to the low adhesion, borosilicate glass allows only unstable printing processes with polymers like HIPS or PP but reaches higher adhesion forces with the most other polymers. The maximum was measured for PLA with a value of 231 N.

Brass is not compatible with the most tested polymers. Only PLA, PETG, PC and PMMA adhered to the used brass sheet. The measured maximum value was also observed for PLA as printing material and had a value of about 85 N.

Another possible use case is the comparison between the same kind of polymer provided by different manufacturers according to the adhesion. A first measurement series was done to compare PETG from the company Filamentworld [26] and a modified PETG called CPE-HG100 from the company fillamentum [27]. For both materials a differential scanning calorimetry was done to determine the glass transition point (T_g_). PETG from Filamentworld has a glass transition temperature of 78 °C and CPE-HG100 one of 84 °C. To ensure comparable process parameters the nozzle temperature was for both materials chosen as 1.4 times T_g_ and the build platform temperature was set to 1.05 times T_g_ (see Table 6).

**Table 6:** Printing parameters of the two tested PETG-filaments.

Printing parameter	PETG	CPE-HG100
Layer height	0.5 mm	
Nozzle diameter	1 mm	
Printing speed	10 mm/s	
Nozzle temperature	230 °C	260 °C
Build platform temperature	96 °C	105 °C

Both polymers were printed onto borosilicate glass with the printing parameters listed in Table 6.

**Fig. 77:**
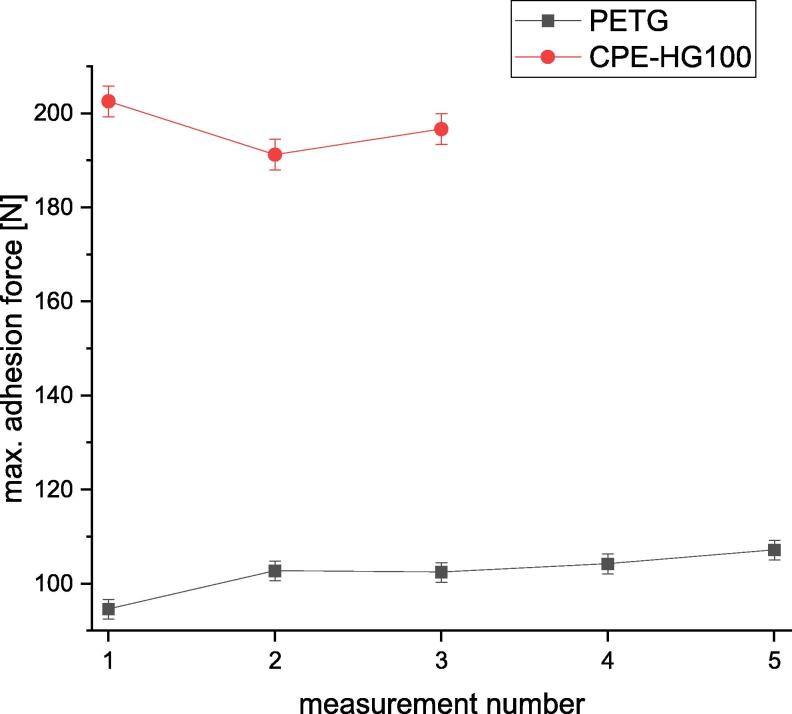
Comparison between the maximum measured adhesion force of PETG from Filamentworld and CPE-HG100 from Fillamentum. The used build surface was borosilicate glass. The error bars show the standard error of the mean calculated from the three respective five measurements.

Fig. 77 shows clearly that CPE-HG100 has a better adhesion than PETG. For CPE-HG100 an adhesion force between 191 N and 202 N with an average of 196.7 N was measured. PETG has an adhesion force between 94 N and 107 N with an average of 102 N. This is about 49 % less in average. Due to the high adhesion between CPE-HG100 and borosilicate glass only three measurements could be done. At the fourth measurement the borosilicate glass broke and the measurement series was not continued.

The here described method and the corresponding measurement device allows now a targeted improvement of the adhesion between printed parts and the build surface. With the possibility to quantify the adhesion it is possible to select a build surface by objective criteria like the maximum reached adhesion force.

Filaments are often available from several manufacturers. This method gives the user a possibility to find the best filament specifically.

The most important is the possibility to targeted improve the process parameters like build platform temperature, nozzle temperature, layer height, etc. In future, further investigations should be done to determine how the adhesion between printed parts and build surface is affected by these parameters. With this knowledge it is possible to choose the optimal parameters regarding adhesion without the need of large parameter studies to be done.

## Declaration of Competing Interest

The authors declare that they have no known competing financial interests or personal relationships that could have appeared to influence the work reported in this paper.
